# Generation and Characterization of a Foxtail Millet (*Setaria italica*) Mutant Library

**DOI:** 10.3389/fpls.2019.00369

**Published:** 2019-03-29

**Authors:** Jing Sun, Ngoc Sinh Luu, Zhenhua Chen, Bing Chen, Xuean Cui, Jinxia Wu, Zhiguo Zhang, Tiegang Lu

**Affiliations:** Biotechnology Research Institute, National Key Facility for Genetic Resources and Gene Improvement, the Chinese Academy of Agricultural Sciences, Beijing, China

**Keywords:** foxtail millet, EMS, mutant, mutation frequency, white panicle

## Abstract

Foxtail millet (*Setaria italica*) is attractive to plant scientists as a model plant because of several distinct characteristics, such as its short stature, rapid life cycle, sufficient seed production per plant, self-compatibility, true diploid nature, high photosynthetic efficiency, small genome size, and tolerance to abiotic and biotic stress. However, the study on the genetic resources of foxtail millet largely lag behind those of the other model plants such as *Arabidopsis*, rice and maize. Mutagenized populations cannot only create new germplasm resources, but also provide materials for gene function research. In this manuscript, an ethyl methanesulfonate (EMS)-induced foxtail millet population comprising ∼15,000 individual M_1_ lines was established. Total 1353 independent lines with diverse abnormal phenotypes of leaf color, plant morphologies and panicle shapes were identified in M_2_. Resequencing of sixteen randomly selected M_2_ plants showed an average estimated mutation density of 1 loci/213 kb. Moreover, we provided an example for rapid cloning of the *WP1* gene by a map-based cloning method. A white panicle mutant, named as *wp1.a*, exhibited significantly reduced chlorophyll (Chl) and carotenoid contents in leaf and panicle. Map-based cloning results showed an eight-base pair deletion located at the sixth exon of *wp1.a* in LOC101786849, which caused the premature termination. *WP1* encoded phytoene synthase. Moreover, the sequencing analysis and cross test verified that a white panicle mutant *wp1.b* was an allelic mutant of *wp1.a*. The filed phenotypic observation and gene cloning example showed that our foxtail millet EMS-induced mutant population would be used as an important resource for functional genomics studies of foxtail millet.

## Introduction

Foxtail millet is a member of the Paniceae tribe (subfamily Panicoideae of the Poaceae) and came from green millet domestication in northern China about for 8000 years ago (Barton et al., 2009). Foxtail millet is extensively cultivated in the developing countries in semiarid and arid regions of Africa, Americas, Asia (Lata et al., 2013) because of its health benefits (a particular balance of nutrients, e.g., starch, protein, dietary fibers, fat, vitamins, and low-glycemic and hypolipidemic effects), good yield with minimal agricultural inputs, and adaptation to different biotic and abiotic stresses such as salinity (Lata et al., 2011), drought (Lata et al., 2011; Feldman et al., 2017; Tang et al., 2017), and fungal diseases (Xu et al., 2011). A healthy and environmentally friendly small crop of foxtail is an increasingly attractive alternative for crop production, and China have planted 70 M ha more foxtail millet in 2018 than in 2017, increasing to 130 M ha^1^.

In recent years, foxtail millet and its ancestor green foxtail (*Setaria viridis*) became more attractive to plant scientists as an alternative model plant because of several distinct characteristics, such as their short stature, rapid life cycle, sufficient seed production per plant, self-compatibility, true diploid nature (2n = 18), and small genome size (515 and 395 Mb, respectively) (Doust et al., 2009; Li and Brutnell, 2011; Huang et al., 2016; Pant et al., 2016). More importantly, foxtail millet and green foxtail are typical C_4_ plants, similar to maize, sorghum and sugarcane, and therefore can be a valuable model plant for studies of C_4_ photosynthesis (Brutnell et al., 2010; Lata et al., 2013; Pant et al., 2016; Huang et al., 2017; Yang et al., 2018). These plants can also be used as model systems for panicoid grasses such as switchgrass (*Panicum virgatum*), and napier grass (*Pennisetum purpureum*) for biofuel studies (Li and Brutnell, 2011; Lata et al., 2013; Pant et al., 2016). The distinct drought tolerant characteristic is very beneficial to dissect the molecular mechanisms for drought tolerance.

Genetic and genomic resources are necessary for studies of gene function. Reference genome sequences and resequencing of the core germplasm of foxtail millet are available to the public (Bennetzen et al., 2012; Zhang et al., 2012; Jia et al., 2013a) and several high density molecular marker maps and integrated databases were developed based on public data (Jia et al., 2013b; Pandey et al., 2013; Muthamilarasan et al., 2014; Yadav et al., 2015; Zhang et al., 2014; Muthamilarasan and Prasad, 2015). Genetic variation resources have been collected, and high-density genetic maps have been constructed (Wang et al., 2012, 2017; Jia et al., 2013b; Muthamilarasan and Prasad, 2015; Fang et al., 2016). Genes controlling agronomically important traits such as branching (Doust et al., 2004) and panicle shape (Xiang et al., 2017) have been cloned using those resources. However, compared with *Setaria viridis*, much less attention has been paid to *Setaria italica*; for example, mutant resources are plentiful for *Setaria viridis* (Brutnell et al., 2010; Huang et al., 2016), but only occasional reports are found for *Setaria italica* (Gupta and Yashvir, 1976). Thus, the construction of a *Setaria italica* mutant library is urgently needed to accelerate functional research. In this study, we described the generation and field phenotype characterization of an ethyl methyl sulfonate-induced foxtail millet mutant population. We also reported the mutation frequency of this population by resequencing and identified a gene that controls carotenoid biosynthesis, namely, *WP1*.

## Materials and Methods

### Mutagenesis and Plant Growth

Newly harvested mature seeds of an elite inbred line Yugu No. 1(the most influential variety in North China because of its strong disease resistance, drought and waterlogging resistance, insensitivity to light and temperature, and wide adaptability) were stored at 4°C for 30 days to break the dormancy, and seeds with at least a 90% germination rate were utilized for chemical mutagenesis. After immersing foxtail millet seeds for 8 h, the soaked seeds were then incubated in a hood with different concentrations of ethyl methyl sulfonate (Sigma-Aldrich, United States) of 0.5, 1.0, 1.5, or 2% in 0.1 M sodium phosphate buffer (pH 7.0) for different times (8, 16, or 24 h). After determining the appropriate concentration and time (1% EMS concentration and 16 h treatment time) based on the germination rate (20–25%), approximately one hundred thousand seeds were treated in EMS solution on a shaker at 80 rpm in dark conditions at room temperature. After incubation, seeds were washed with running water for 4 h, germinated on wet filter paper in a 25 × 30 × 10 cm plastic box covered with transparent cling film at 28°C for 3 days in darkness and then transferred to a greenhouse at 26–30°C with natural light conditions (14 h light/10 h dark). Germinated seedlings of approximately 0.3–0.5 cm in height were then transferred to a greenhouse and grown in soil. The spikelet of each plant was paper-bagged before flowering to prevent pollen contamination from other plants. Golden spikelets with mature seeds were harvested, air-dried completely and kept at 4°C.

### Mutant Characterization

Approximately 1000 seeds for each line were sown in an experimental field in June. All the seedlings were first characterized by the leaf color, i.e., albino or yellow, at 15 days after germination, and then excess seedlings were thinned to ensure the remaining seedlings were grown at 15 cm for a line and 30 cm to a row, with approximately 30 M_2_ plants for each line grown in the field. All plants were managed according to standard protocols for watering, fertilizing and other practices. The remaining M_2_ plants grown in the field were subjected to carefully recorded observation and characterization every 2 weeks.

### Chlorophyll and Carotenoid Contents Measurement

The mutant *wp1* and wild type chlorophyll contents were measured by using spectrophotometrical method (Arnon, 1949). In total, 400 mg leaves and panicles were ground into powder in liquid nitrogen and then shifted to a 10 ml tube. Five milliliters acetone (80% V/V) was put into the tube and mixed thoroughly, and the tube was kept overnight in darkness. Centrifugation was carried out for 15 min (∼750 g) at 4°C. The supernatant was washed three times with the same amount of hexane. The chlorophyll contents were determinated with spectrophotometer (Beckman Coulter DU 800 UV/Vis).

### Mutant Frequency Evaluation

Sixteen foxtail millet mutant samples were randomly selected and grown in an incubator with 10 h of light at 28°C per day for a month. To construct the library for resequencing, total genomic DNAs of 4th-leaf-stage leaf tissue were isolated by CTAB method. 1.5 μg DNA each sample was used for construct the sequencing libraries.

Sequencing libraries were created based on a TruSeq Nano DNA HT Sample Preparation Kit (Illumina, United States) as followed. Firstly, sonication treatment made the DNA sample fragment to a size of 350 bp, secondly, DNA fragments were end-blunted, A-tailed, and ligated with the full-length adapter for Illumina sequencing, thirdly, the PCR product was amplified and purified. The size was decided by an Agilent 2100 Bioanalyzer and quantity was analyzed using Q-PCR.

The libraries constructed of these sixteen lines were sequenced by an Illumina HiSeq platform, and 150 bp paired-end reads were set up with ∼ 350 bp size. Average 38x sequencing depth per sample was produced and 277.39 Gb of clean bases were produced in total. The alignment of reads was used to build a consensus genome sequence for foxtail millet^2^. The GATK tools package (McKenna et al., 2010) was used to detect SNP sites and small INDEL s (insertion and deletion), which included local realignment, and the duplicates were marked by Picard^3^ to ensure the accuracy. According to standard procedure, if less than 60% of the loci appear in samples, the loci is regarded as a SNP or INDEL site, otherwise or not.

### Map-Based Cloning and Candidate Gene Identification

To map WP1 gene, a *wp1.a* × “SSR41” F_2_ population was created. In F_2_ progeny, a total 246 white panicle individuals were collected. Their DNA samples were extracted and used to map. Ten samples with equal DNA concentration were mixed as a pool. Three pools were used for rough mapping. A set of high resolution SSR/ INDEL s molecular markers were designed based on the published primer sequences (Zhang et al., 2014; Supplementary Table S2) and 30× genome resequence result both Yugu No.1 and SSR41. Using this set of SSR/ INDEL markers, *WP1* gene was mapped on chromosome 4. By further screen the 246 F_2_ recessive individuals, we narrowed the interval to 80 kb using newly developed primers (Supplementary Table S1).

### Allelic Analysis

To determine whether *wp1.b* was allelic with *wp1.a*, a cross was conducted between white panicle mutant *wp1.a* and *wp1.b*. In F_1_ progeny, the phenotype was investigated. If *wp1.b* was not allelic with the *wp1.a*, the panicle phenotype would restore to the normal level in F_1_ progeny.

## Results

### EMS Induced Foxtail Millet Population Construction

**Figure 1:**
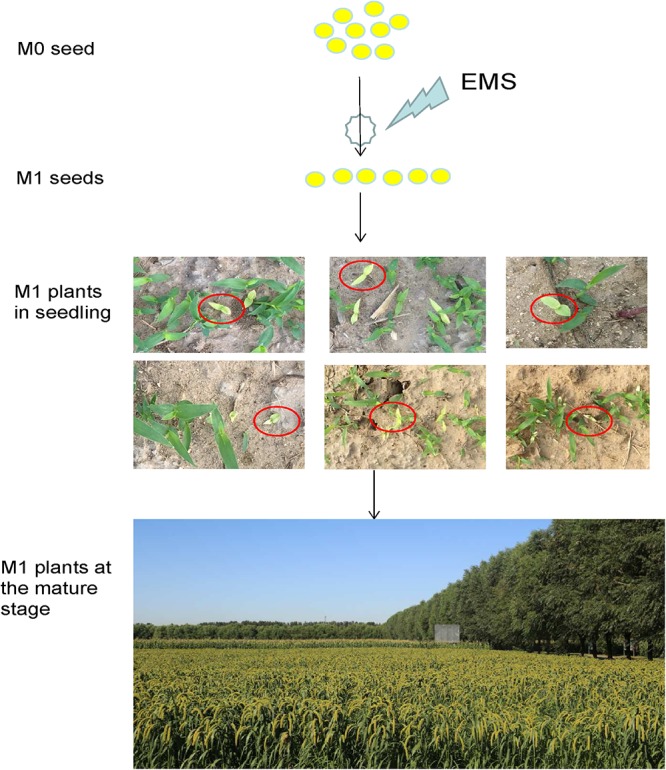
Development of a foxtail millet ethyl methanesulfonate (EMS)-induced mutant population. M0 seeds were mutated and propagated, and a single M2 seed was selected from each chimeric M1 plant.

To determine the appropriate concentration of EMS mutagenesis, four EMS solution concentrations (v/v) of 0.5, 1.0, 1.5, or 2% were used to treat seeds of Foxtail millet cv. Yugu No.1 overnight. At the 0.5% concentration, at least 50% of Yu1 seeds germinated, whereas at the 2% concentration, the seed germination rate was only 2.5%. We selected the 1% concentration as an appropriate concentration with a 25% seeds germination rate (Supplementary Figure S1A). The optimal treatment time was determined by testing three times: 8, 16, and 24 h. At the 1% concentration, the 16 h treatment resulted in a 20% seed germination rate. Thus, we selected the 1% EMS concentration and 16 h treatment time for further trials (Supplementary Figure S1B). Approximately 100,000 seeds of foxtail millet cv. Yugu No.1 were mutagenized with EMS. Of these seeds, 20,000 seeds normally germinated as M_0_ progeny. Approximately 5000 seeds resulted in M_0_ progeny plants that did not produce seeds owing to albino leaf, sterility or severe dwarfing (Figure 1).

### Phenotypic Screening in M_2_ Plants

**Figure 2:**
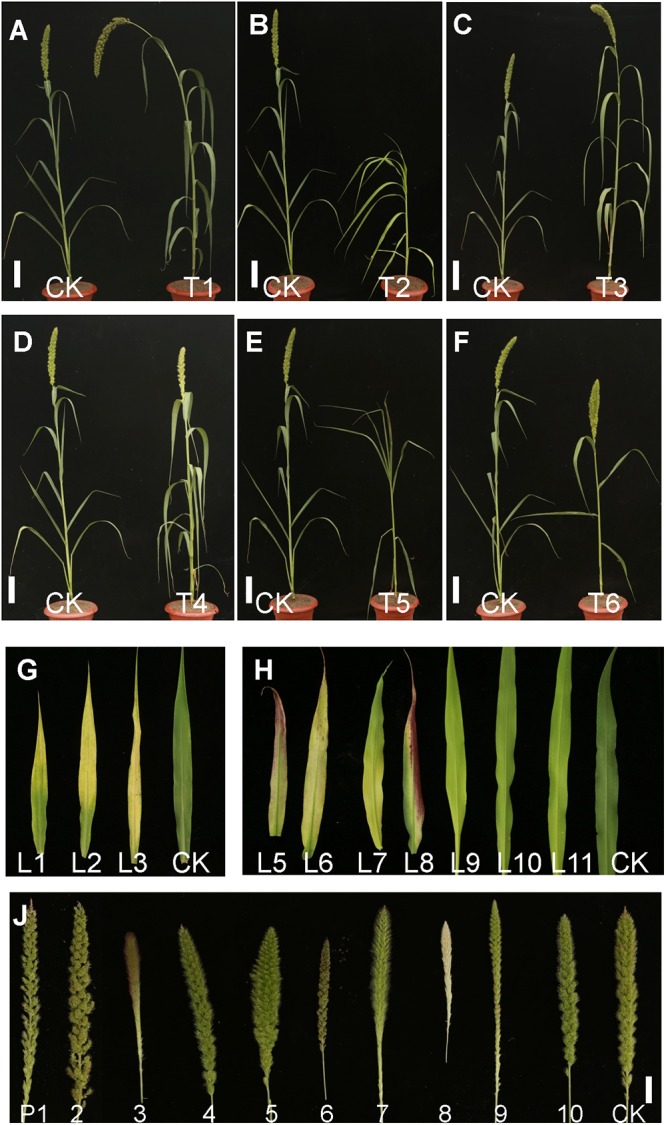
Pictures of mutants of foxtail millet in the M2 progeny from the EMS library. **(A)** T1, Height mutant. **(B)** T2, Dwarf mutant. **(C)** T3, Large panicle mutant. **(D)** T4, Yellow leaf mutant. **(E)** T5, Rolling leaf mutant. **(F)** T6, Large leaf angle mutant (Bar = 5 cm). **(G)** Different leaf phenotype mutants. L1–L11, Yellow leaf mutants with different chlorophyll contents. **(H)** Different panicle variation mutants. P1–P2, Loose panicle mutants; P3, Red awn mutant; P4, Small panicle mutant; P5, Large panicle mutant; P6, Red panicle mutant; P7, Long awn mutant; P8, White panicle mutant; P9, Degenerated panicle mutant; P10, Dense panicle mutant; CK, Yugu No. 1 (Bar = 0.5cm).

Seeds of approximately 15,000 M_1_ plants were harvested. All M_1_ seeds (minimum 20 plants per line) were planted to screen for plant morphological mutants. According to the phenotypic variation at the seedling and mature stages, mutants were classified into six types based on leaf color, heading date, plant height, senescence, leaf shape, and panicle development (Table 1 and Figure 2). A total of 1353 independent mutants with a phenotype visible to the naked eye were obtained. The mutation frequency reached 9.02% in the M_2_ plants (Table 1). The largest mutation type was that of leaf color mutant, wherein the albino mutant numbers reached 425, with the highest mutant frequency of 2.83%. Notably, panicle phenotypic variation was rich in our foxtail millet EMS mutant library, which reached 348 with the high mutant frequency of 2.32%. Morphological variation of various types of panicles included the dense panicle, loose panicle, degenerated panicle, white panicle, small panicle, large panicle, red panicle, and long awn (Figure 2), which indicated that the foxtail millet panicle morphological variation mutants could be a valuable resource for studying panicle development.

**Table 1:** Characterization of different mutant types of foxtail millet in the M2 plants [15,000 EMS lines (minimum 20 plants per line)] from the EMS library.

Classification of mutant		M2 plants	
		Mutant lines	Mutation frequency rate (%)
Leaf color	Albino leaf	425	2.83
	Yellow leaf	54	0.36
Heading date	Early flowering	25	0.17
	Late flowering	43	0.29
Plant Height	High	35	0.23
	Dwarf	152	1.01
Senescence	Lesion mimic	84	0.56
	Early senescence	102	0.68
Leaf shape	Rolled leaf	47	0.31
	Large leaf angle	38	0.25
Panicle	Dense panicle	84	0.56
	Loose panicle	45	0.30
	Degenerated panicle	58	0.39
	White panicle	3	0.02
	Small panicle	57	0.38
	Large panicle	24	0.16
	Long panicle	28	0.19
	Red panicle	14	0.09
	Long awn	35	0.23
Total		1353	9.02

### Mutation Density in the EMS Induced Population

**Figure 3:**
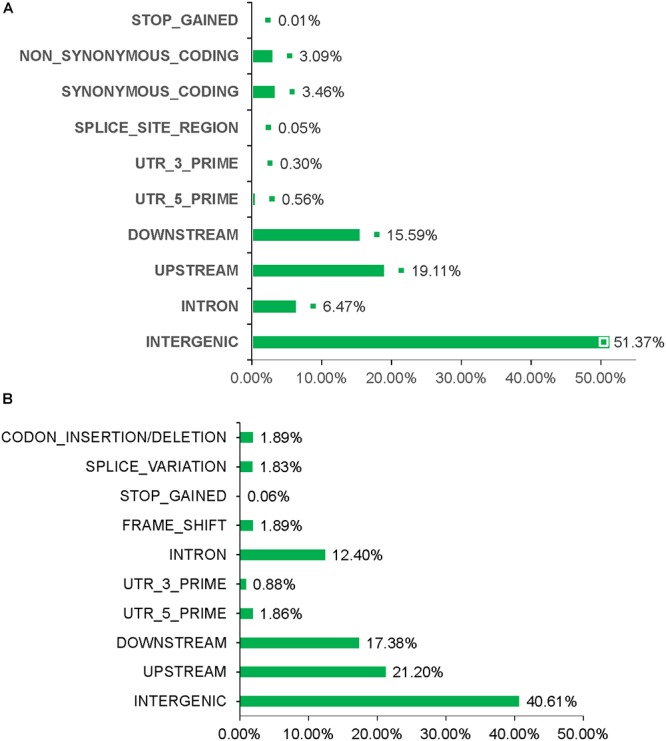
Overview of INDEL/SNP polymorphism data generated analyzing the sixteen resequenced mutant lines. **(A)** Different variation types for SNPs. **(B)** Different variation types for INDEL.

Total > 38 × coverage depth was generated and over 98% of the reads were uniquely mapped to the Yugu No.1 reference genome. Based on comparison on the Yugu No.1 reference genome, the EMS-induced SNP/INDEL results are shown in Table 2. A total number of INDEL/SNPs were 36711, with 2295.44 average were identified compared with Yugu No.1, corresponding to an average estimated mutation density of 1/213 kb. Among the 16 resequenced mutant lines, intergenic variations were the maximum type and occupied at least 45.75% of the total variation. The regulatory region variation including upstream region, downstream region, 5′-untranslated region (5′-UTR), and 3′-untranslated region (3′-UTR) summed to 885.13 loci, with the proportion 38.56% of the total variation, as shown in Table 2. The variation that occurred within open reading frames (ORFs) containing intron, stop gained, splice variation, frame shift, codon insertion/deletion, synonymous coding, and nonsynonymous coding reached 359.68 loci, with the proportion 15.66% of the total variation. Further, SNP or INDEL loci were analyzed. The SNP results are shown in Table 3, and the INDEL results are shown in Table 4. On average, each line had 1100.94 loci for SNP variation and 1194.50 loci for INDEL variation. Average intergenic variation had 565.56 loci for SNPs and 1050.63 loci for INDELs, with proportions of 51.37 and 45.77% of the total variation, respectively (Figure 3 and Tables 3, 4).

**Table 2:** Overview of all variation data containing INDELs/SNPs generated analyzing the 16 resequenced EMS induced mutant lines.

INDEL/SNP type	EMS-1	EMS-2	EMS-3	EMS-4	EMS-5	EMS-6	EMS-7	EMS-8	EMS-9	EMS-10	EMS-11	EMS-12	EMS-13	EMS-14	EMS-15	EMS-16	Average
INTERGENIC	932	1045	1029	980	1321	981	1054	963	1202	995	1012	1149	954	1149	911	1133	1050.63
UPSTREAM	416	416	446	436	739	438	451	410	554	386	426	482	416	489	367	547	463.69
DOWNSTREAM	367	361	380	352	519	351	355	367	438	380	336	391	352	388	319	411	379.19
UTR_5_PRIME	26	24	23	32	60	27	28	20	43	21	27	20	30	23	17	34	28.44
UTR_3_PRIME	12	14	13	14	17	15	14	14	18	7	12	16	12	16	10	17	13.81
INTRON	205	233	227	221	292	213	204	212	243	204	204	213	214	232	175	216	219.25
STOP_GAINED	1	1	1	1	0	1	0	0	1	1	0	1	1	1	1	2	0.81
SPLICE_VARIATION	21	19	29	22	26	28	22	21	22	22	21	28	26	22	22	6	22.31
FRAME_SHIFT	20	18	28	21	25	26	22	20	21	20	21	26	24	21	20	29	22.63
CODON_INSERTION/DELETION	22	20	30	23	25	28	22	20	23	22	21	28	26	23	22	6	22.56
SYNONYMOUS_CODING	33	25	37	25	66	39	41	28	46	26	39	34	43	31	39	57	38.06
NON_SYNONYMOUS_CODING	29	31	29	29	60	24	30	18	49	32	29	36	28	30	38	53	34.06
SUM	2084	2207	2272	2156	3150	2171	2243	2093	2660	2116	2148	2424	2126	2425	1941	2511	2295.44

**Table 3:** Overview of single nucleotide polymorphism (SNP) data generated analyzing the 16 resequenced EMS induced mutant lines.

SNP type	EMS-1	EMS-2	EMS-3	EMS-4	EMS-5	EMS-6	EMS-7	EMS-8	EMS-9	EMS-10	EMS-11	EMS-12	EMS-13	EMS-14	EMS-15	EMS-16	Average
INTERGENIC	477	542	528	517	772	523	574	513	678	521	544	609	493	598	486	674	565.56
INTRON	67	81	62	77	103	69	66	82	75	66	55	69	62	69	48	88	71.19
UPSTREAM	183	178	190	178	402	199	207	178	266	171	174	211	170	219	151	290	210.44
DOWNSTREAM	154	158	168	164	278	152	158	162	227	168	129	178	153	151	129	217	171.63
UTR_5_PRIME	6	5	4	6	14	5	7	5	13	5	7	3	5	5	2	7	6.19
UTR_3_PRIME	5	3	4	3	2	2	3	3	4	2	4	4	1	4	3	5	3.25
SPLICE_SITE_REGION	0	0	0	0	1	1	0	1	0	1	0	1	1	0	1	1	0.50
SYNONYMOUS_CODING	33	25	37	25	66	39	41	28	46	26	39	34	43	31	39	57	38.06
NON_SYNONYMOUS_CODING	29	31	29	29	60	24	30	18	49	32	29	36	28	30	38	53	34.06
STOP_GAINED	0	0	0	0	0	0	0	0	0	0	0	0	0	0	0	1	0.00
SUM	954	1023	1022	999	1698	1014	1086	990	1358	992	981	1145	956	1107	897	1393	1100.94

**Table 4:** Overview of insertion/deletion nucleotide polymorphism (INDEL) data generated analyzing the 16 resequenced EMS induced mutant lines.

INDEL type	EMS-1	EMS-2	EMS-3	EMS-4	EMS-5	EMS-6	EMS-7	EMS-8	EMS-9	EMS-10	EMS-11	EMS-12	EMS-13	EMS-14	EMS-15	EMS-16	Average
INTERGENIC	455	503	501	463	549	458	480	450	524	474	468	540	461	551	425	459	485.06
UPSTREAM	233	238	256	258	337	239	244	232	288	215	252	271	246	270	216	257	253.25
DOWNSTREAM	213	203	212	188	241	199	197	205	211	212	207	213	199	237	190	194	207.56
UTR_5_PRIME	20	19	19	26	46	22	21	15	30	16	20	17	25	18	15	27	22.25
UTR_3_PRIME	7	11	9	11	15	13	11	11	14	5	8	12	11	12	7	12	10.56
INTRON	138	152	165	144	189	144	138	130	168	138	149	144	152	163	127	128	148.06
FRAME_SHIFT	20	18	28	21	25	26	22	20	21	20	21	26	24	21	20	29	22.63
STOP_GAINED	1	1	1	1	0	1	0	0	1	1	0	1	1	1	1	1	0.75
SPLICE_VARIATION	21	19	29	22	25	27	22	20	22	21	21	27	25	22	21	5	21.81
CODON_INSERTION/DELETION	22	20	30	23	25	28	22	20	23	22	21	28	26	23	22	6	22.56
SUM	1130	1184	1250	1157	1452	1157	1157	1103	1302	1124	1167	1279	1170	1318	1044	1118	1194.50

### Map-Based Cloning Identified the Causal Gene WP1

**Figure 4:**
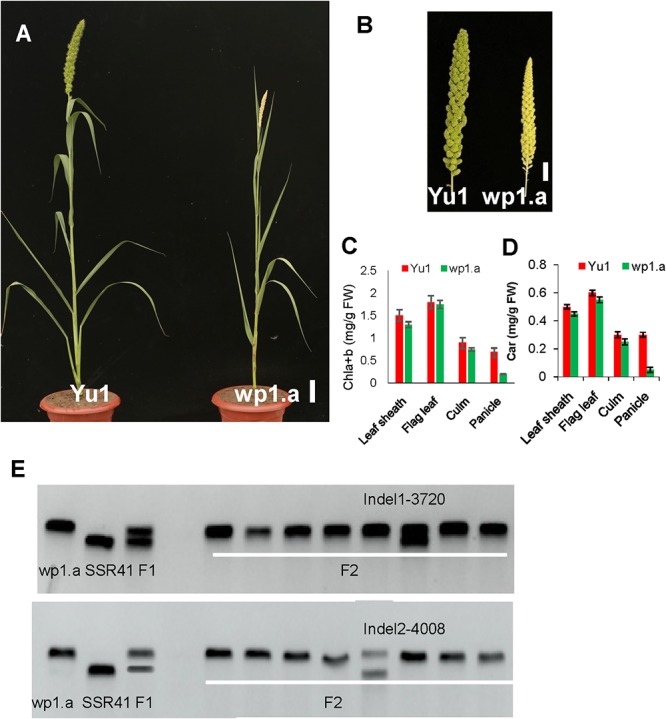
Phenotypic analysis of the white panicle mutant (wp1.a) and the wild type. **(A)** Phenotype comparison between a white panicle mutant (wp1.a) and the wild type (Yu1) at the mature stage (Bar = 5cm). **(B)** Panicle comparison between a white panicle mutant (wp1.a) and the wild type (Yu1) at the mature stage (Bar = 0.5cm). **(C,D)** Comparison of the pigment contents in different tissues between wp1.a mutants and the wild type; Chla+b, total chlorophyll; Car, carotenoid. Bars represent the sd of three measurements. Student’s *t*-test was performed on the raw data; asterisk indicates statistical significance at *P* < 0.01. **(E)** Rough mapping analysis for the WP1 gene using the F_2_ progeny. The rough mapping indicated that WP1 was located between Indel1 and Indel2 of chromosome 4.

To evaluate whether our foxtail millet EMS induced population was beneficial to clone a gene, a white panicle mutant (*wp1.a*) was selected to identify the causal gene (Figure 4A). The *wp1.a* plant was crossed with another landrace “SSR41.”

In F_1_ progeny, all the plants displayed the normal panicle phenotype. In F_2_ progeny, the panicle phenotype segregated in normal phenotype plants (156) and white panicle phenotype plants (45), which accord with 3:1 ratio (χ^2^ = 1.21 < χ^2^_0.05,1_), indicating that the *wp1.a* white phenotype was controlled by a single recessive mutation.

Notably, no great differences occurred except for panicle color and plant height between the *wp1.a* plant and the wild type (Figure 4A,B). The *wp1.a* plant had reduced height. Pigment assays showed that the chlorophyll (Chl) contents of leaf sheath, flag leaf and culm in *wp1.a* only were slightly lower than those of the wild type (Figure 4C). The content of chlorophyll in *wp1.a* panicle only reached 30% of that in wild type. (Figure 4D).

**Figure 5:**
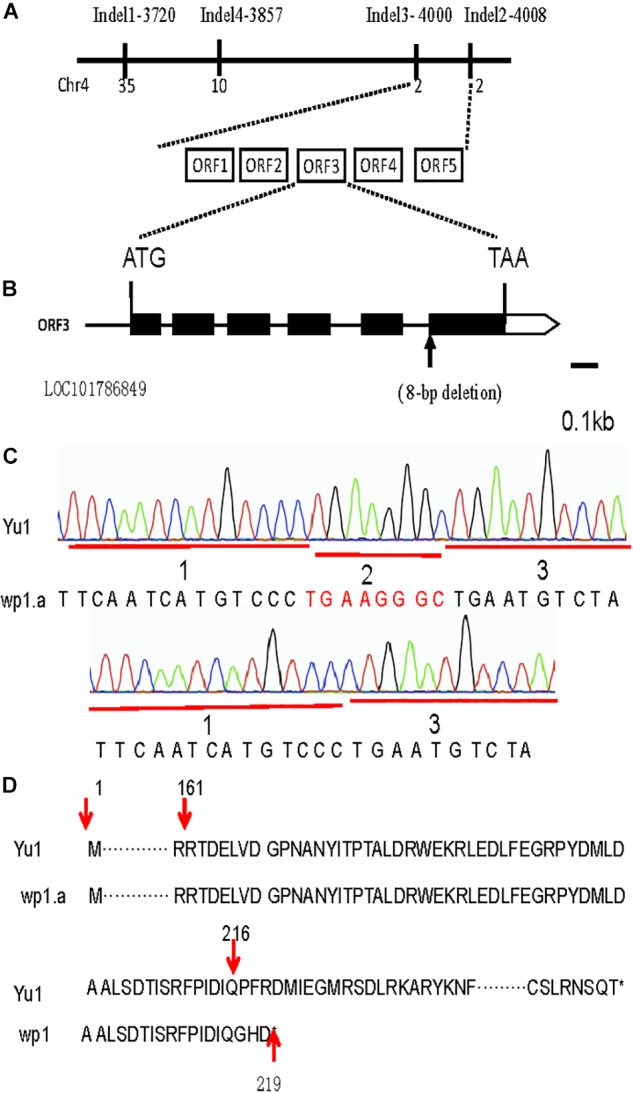
Map-based cloning of the WP1 gene. **(A)** The *WP1* locus was narrowed down to an 80 kb region between InDel markers 4000 and 4008 of chromosome 4 using 246 F_2_ homozygous white panicle plants. Five open reading frames (ORF1-5) were predicted in the mapped region. **(B)** An eight-base pair deletion located at the junction of the fifth intron and sixth exon of LOC101786849 in *wp1*. ATG and TAA were the start and stop codons, respectively. **(C)** The mutant site comparison of LOC101786849 between white panicle (wp1) and Yu1. **(D)** The eight-base pair deletion caused the LOC101786849 transcript to not be normally cut and led to premature termination.

For rough mapping cloning, molecular markers were selected equally distributed on the 9 chromosomes with an average physical interval of approximately 5 Mb. A bulked pool analysis revealed that the molecular markers Indel1 -3720 and Indel2-4008 closely linked to WP1 gene ranging from 37.25 to 40.08 cM (Figure 4E), implying that *WP1* is positioned at the end of chromosome 4. For fine mapping, a total 246 F_2_ homozygous recessive individuals were used, and INDEL markers (Indel3-4000, Indel4-3857) were developed (Figure 5A). Finally, *WP1* was mapped to an 80 kb region between markers lndel3-4000 and lndel2-4008 (Figure 5A), which contained five candidate genes. We sequenced the five open reading frames in the *wp1.a* plant and found only an eight-base pair deletion within LOC101786849 (Figure 5B). The eight-base pair deletion located at the sixth exon in *wp1.a* caused the LOC101786849 transcript led to premature termination (Figure 5C).

In our EMS library, there are five white panicle mutants. We sequenced the LOC101786849 for the five white panicle mutants. One of the white panicle mutants, named white panicle (*wp1.b)*, was found to have a base pair change located at the junction of fifth intron and sixth exon of LOC101786849. WP1 transcript was checked between the *wp1.b* and Yu1. The PCR product was amplified by forward primer fp1 and reverse primer fp2 approaching the splicing site. The result showed that PCR product in *wp1.b* had a higher molecular weight (∼820bp) than in Yu1 (∼ 750 bp), which indicated that mutant splicing site (G-A) affected the splicing of LOC101786849 transcript products in *wp1.b* (Supplementary Figures S2A–D).

Cross between *wp1.a* and *wp1.b* was proceeded. The 21 seeds were gained successfully in F_0_. All the 21 plants showed the white panicle phenotype in F_1_ progeny. The analysis strongly verified that a white panicle mutant *wp1.b* was an allelic mutant of *wp1.a*. This result demonstrated that the methods could be used to quickly clone a foxtail millet gene using our EMS induced mutant library.

## Discussion

Foxtail millet, as the oldest cultivated millet crops, needs warm weather and minimal water to ripen rapidly in dry and hot months of each year. In 2012, two departments both the Joint Genome Institute (JGI) of the Department of Energy, United States, and BGI (Beijing Genome Initiative), China, announced and published the foxtail millet genome sequence (Zhang et al., 2012). The foxtail millet genome sequence is closely related to several bioenergy crops such as pearl millet, napier grass (*Pennisetum purpureum*), switchgrass (*Panicum virgatum*). Compared with several bioenergy crops, foxtail millet has the advantage of being selected as a model system due to low amount of repetitive DNA, its small genome, short life cycle, more seeds, rich genetic diversity and inbreeding nature (Doust et al., 2009). At present, foxtail millet has been selected as a C_4_ model crop to explore crop architectural, evolutionary genomics from C_3_ to C_4_, and physiological characteristics of the C_4_ grass crops (Doust et al., 2009; Li and Brutnell, 2011). *S. viridis* is ancestor of *S. italica. S. viridis* widely distributed in the earth, whereas *S. italica* is one of the most earliest domesticated crops in China. *S. viridis* and *S. italica* have rich natural diversity, which provided a rich resource to unearth novel gene/allele for the important agronomical traits.

Jia went on the population genetics analysis of *S. italica.* He resequenced the 916 diverse accessions of *S. italica* and associated analysis using GWAS in *S. italica*. 47 agronomically important traits were associated with 512 loci including flowering time, plant height and inflorescence architecture (Jia et al., 2013a). China’s foxtail millet resources account for more than 80% of the world’s stock, but for a long time, due to the lack of reliable and efficient molecular marker information, researchers still lack of understanding of the population structure of these genetic resources, thus limiting the efficient exploration and deep utilization of millet genetic resources. Jia’s work provided a large amount of basic data information for genetic improvement and gene discovery of foxtail millet, greatly enrich the research of comparative genetics and functional genomics of cereal crops, and would have a profound impact on the improvement of cereal crops and genetic analysis of energy crops. In addition, *S. viridis* diversity samples have also been collected across the United States (Huang et al., 2016). Together, these collections provide an unprecedented opportunity for evolutionary research and domestication studies. However, compared with other staple cereal crops, not much research has been conducted on *S. italica* mutant resources for the development of genetic and functional resources. At present, the *S. viridis* mutant populations (NMU) and *S. italica* mutant populations (EMS) have also been constructed in the Brutnell and Diao labs (DDPSC and Chinese Academy of Agricultural Sciences, respectively), offering a lot of useful mutants resources for genetic studies in *Setaria*. In this study, we constructed a large capacity foxtail millet cv. Yugu No. 1 mutant library. Various mutant phenotypes including those for leaf color, plant stature, and panicle shape were obtained at a high (9%) mutation percentage. The mutation frequency was similar as those of other previous rice (Suzuki et al., 2008), soybean (Li et al., 2017), and maize mutant libraries (Till et al., 2004). Notably, panicle phenotypic variation was rich in our foxtail millet EMS mutant library, which reached up to 2.32% mutant frequency. Morphological variation of various panicle phenotypes indicated the foxtail millet mutant library would provide a valuable resource for studying panicle development. This foxtail millet mutant library enriched the foxtail millet germplasm and will accelerate functional genomics research.

EMS at a high concentration induced a high frequency of loci variation. Since multiple mutations may mask the mutant phenotype of interest, functional mutations need to be identified by cross analysis experiments with wild-type plants. By selecting an optimal EMS concentration, we avoided higher frequency variation, which was a result verified by random sequencing for sixteen M_2_ mutant materials. Based on random sequencing, the mutation density in our mutant population reached 1 mutation per 213 kb. EMS is an alkylating agent causing mainly G/C to A/T transitions. SNP variations accord with expectations, but there are many indel variation in each line after EMS treatment. We analysis the reason of so many indels. From the InDels length distribution (Figure S3 in the new version), we found that a large number of INDELs mainly center on insertion and deletion only 1 bp in length. Frankly speaking, there must be a lot of ineffective INDELs results that lead to overnumber. Other reason is, our SNP/INDELs analysis procedure adopted is looser standard (that less than 60% of the loci appears in samples), If the strict standards was adopted such as less than 40% of the loci appears in samples, the loci (SNP or INDELs) would become more few. LOC10178684 mutation in wp1.b happens from G to A, which conforms to this mutagenesis rule. Compared with the rice mutation frequency (1 mutant site/265 kb) (Suzuki et al., 2008), Arabidopsis mutation frequency (1 mutant site/89 kb) (Martín et al., 2009), tomato mutation frequency (1 mutant site/737 kb) (Okabe et al., 2011), sorghum mutation frequency (1 mutant site/526 kb) (Xin et al., 2008), maize (1 mutant site/485 kb) (Till et al., 2004), the mutation frequency in our foxtail mutant library was basically similar as the these species, sometimes even lower, which indicated that one test cross or two could reduce the background and reduce the complexity of confirmation of a functional gene.

Protocols for map-based cloning and bulked segregantanalysis (BSA) are now widely used for the discovery of causal genes in a time-efficient and cost-effective manner. Using these methods, AUX1which controlled the inflorescence architecture, was cloned in *S. viridis* (Huang et al., 2017). Similarly, Xiang (Chinese Academy of Agricultural Sciences, China) reported using this method to clone genes in *S. italica* that control grain size (Xiang et al., 2017). A transposon tagging population in *S. viridis* had been constructed in the Brutnell lab at DDPSC (Personal communication), strengthening resources for both forward and reverse genetic studies. Using the BSA cloning method, we provided an example for rapid cloning with the *WP1* gene using F_2_ cross progeny. An eight-base pair deletion located at the sixth exon of *wp1.a* in LOC101786849 caused the premature termination in *wp1*. An allele *wp1.b* verified that WP1 was responsible for white panicle phenotype. The WP1 gene encoded a member of the phytoene synthase family. Phytoene synthase (PSY) is the first step in the synthesis of carotenoids. It makes the C_20_-geranyl diphosphate (GGPP) molecule head-to-head condensation to produce a C40 carotenoid phytoene molecule. Subsequently, phytoene undergoes four desaturation reactions and produce lycopene (Cunningham and Gantt, 1998). Due to the lack of carotenoids, mutants with deficiencies in carotenoid precursor synthesis exhibit a variety of phenotypes, such as albino or pale seedlings that are not viable in light, which support the conclusion that defects in a carotenoid precursor are related with chloroplast development/synthesis (Fang et al., 2008).

Because the *wp1.a* mutant had a white panicle phenotype; the indication was that the chloroplast development/synthesis was indirectly influenced in the w*p1.a* mutant. This example verified that our EMS mutant library was beneficial for cloning a gene quickly by map-based cloning methods. In the future, our foxtail millet mutants could be primarily abiotic stress tolerant, particularly to drought and salinity, with high photosynthetic efficiency; therefore, manipulating these agronomic traits can improve its water use efficiency in genetic engineering for abiotic stress tolerance and breeding for high photosynthetic rate.

## Author Contributions

ZZ constructed the foxtail millet mutant library. ZZ, NL, JW, and XC conceived the original screening and cross experiments. ZZ, ZC, JS, NL, and BC analyzed the phenotypes and cloned WP1. ZZ and TL supervised and contributed to the writing.

## Conflict of Interest Statement

The authors declare that the research was conducted in the absence of any commercial or financial relationships that could be construed as a potential conflict of interest.

## Footnotes

1 http://www.fao.org/faostat/zh/#data/QC

2 https://www.ncbi.nlm.nih.gov/genome/10982

3 http://sourceforge.net/projects/picard/
