# Supplementary material for: Generation and Characterization of a Foxtail Millet (Setaria italica) Mutant Library
Source: Front Plant Sci. 2019 Mar 29;10:369. doi: 10.3389/fpls.2019.00369 (PMC6455083; doi:10.3389/fpls.2019.00369)
Supplement: Supplementary file 1 [file Data_Sheet_1.docx]

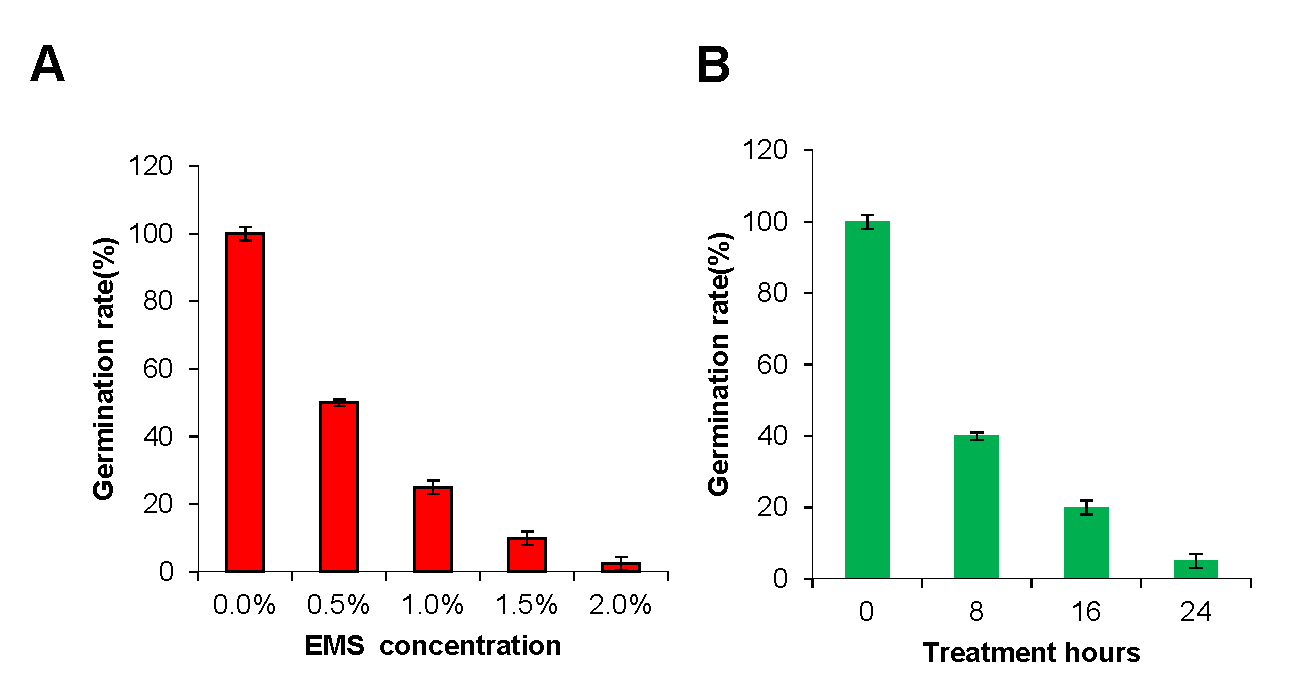


**Figure.S1. Selection of the appropriate EMS concentration and treatment time.**

A. Four EMS solution concentrations (v/v) of 0.5, 1.0, 1.5 or 2% were used to treat seeds of foxtail millet cv. Yugu1 (Yu1) overnight.

B. The treatment times of 8, 16, and 24 h for 1%EMS were used to test the seeds.


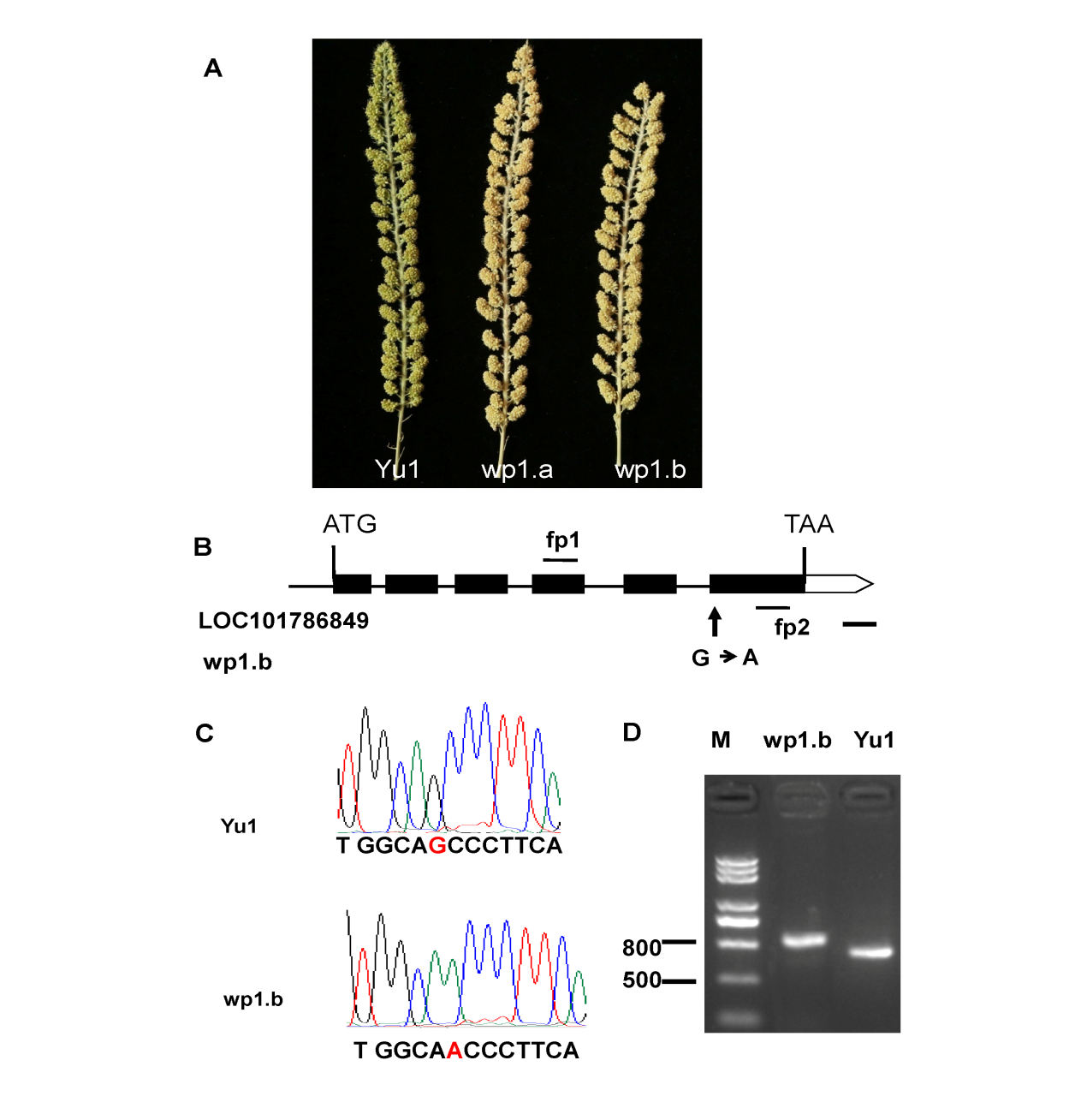


**Figure S2. The allele analysis of the white panicle mutant.**

1. Panicle comparison of Yu No.1(Yu1), wp1.a and wp1.b.

B. A base pairs change located junction of fifth intron and sixth exon of LOC101786849 in *wp1.b*. ATG and TAA were the start and stop codons, respectively.

C. The mutant site sequencce comparison of LOC101786849 between white panicle (*wp1.b*) and Yu1.

D. WP1 transcript was checked between *wp1.b* and Yu1. The PCR product was amplified by foward primer fp1 and reverse primer fp2 approaching the splicing site.The result showed that PCR product in *wp1.b* had a higher molecular weight(~800bp) than in Yu1(~ 750 bp),which indicated that mutant splicing site (G-A)affected the splicing of LOC101786849 transcript products in *wp1.b.*

**
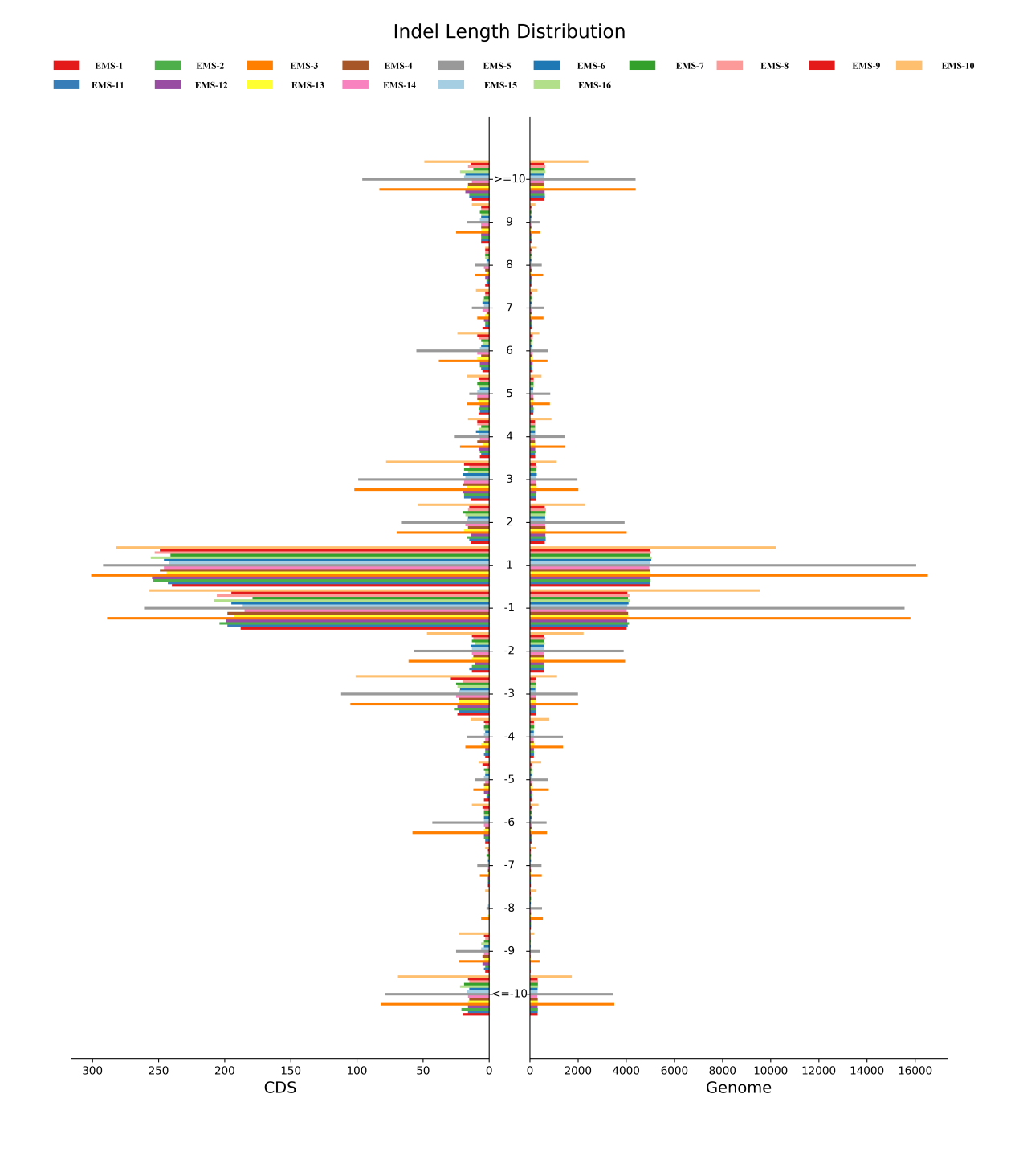
**

**Figure S3. The analysis of the INDELs length distribution.**

Longitudinal coordinate is the length of InDel (< 10 bp), greater than 0 (>0) is Insertion, less than 0 (<0) is Deletion. It is obvious that most of them are concentrated in insertion and Deletion. It is obvious that a large number of InDels mainly center on insertion and deletion only 1 bp in length. Directly speaking, there must be a lot of ineffective indel results that lead to overnumber.

**Table S1.** Primers used for fine mapping WP1 and sequencing.

| Mapping primers | | |
| --- | --- | --- |
| Marker | Forward sequence (5’-3’) | Reverse sequence (5’-3’) |
| Indel1-3720 | TTACACAGTCGGTGGGCAGT | AACATCCCACAACAGAGTGC |
| Indel2-4008 | AGCGGGAGGAATCTTGAGGA | TGAAGGCAGCCCAATACAGC |
| Indel3-3857 | TTTCCATCCAAACAAGCCCTC | CCCATCTTCCAGTGTTCCAATC |
| Indel4-4000 | TGCCAAGGAACAGAAACCATC | GACGGAATCAAAGAGCGACTT |
| Primers for sequencing WP1 gene | | |
| WP1-se1-F/R | GGAGCCCATGCATCACTAAG | CCGCTCTTTCTGATACCATCTC |
| WP1-se2-F/R | TCCAAGGCTGTCGGCGTAAT | TCATCCACCCATCCCAGACT |
| WP1-se3-F/R | GTCTTGGCATACTCCTCGCA | CCACACATTGCTTGATGGCTA |
| Primers for checking WP1 transcript | | |
| FP1/FP2 | CTTCAGGGACATGATTGAAG | CTGGCTATTTCTCAGTGAG |

Table S2. Primers Sequences and polymorphism information confirmed in Setaria accessions (from BMC Genomics. 2014, 15:78).

| molecualr marker | Primer sequence(5’-3’) | Tm (℃) | Expected size (bp) | Chromosome (Location, Kb) |
| --- | --- | --- | --- | --- |
| SICAAS1001 | F-AGCAGCAGTGCTATCATGGCGT | 57 | 112 | Chr.1 (102.6) |
|  | R-ACAGTGCCGTCGTCTGCTCGAT |  |  |  |
| SICAAS1067 | F-GCATACAAGATCCTCGCAGAC | 58 | 191 | Chr.1(120.6) |
|  | R-TGCAATCACCAGAGAGAAGCTA |  |  |  |
| SICAAS1068 | F-CAAGCGAAACCGACCTCT | 57 | 113 | Chr.1(192.6) |
|  | R-GGAGGGAGCTCTTCCTGATT |  |  |  |
| SICAAS1014 | F-GTTAATTAGTGGCAATCCATGC | 51 | 211 | Chr.1 (230.2) |
|  | R-CCTCTTGAGAGGTGGAATTATTATG |  |  |  |
| SICAAS1002 | F-CGAACTACGGCAGGTCGGCA | 59 | 121 | Chr.1 (622.7) |
|  | R-GGTTGGGGCTCTATGTGGCTGT |  |  |  |
| SICAAS1003 | F-CATGACAGATTTGATTACCATGAGC | 53 | 137 | Chr.1 (1,567.8) |
|  | R-TTAGGCAAGTACTCCAGAACTCA |  |  |  |
| SICAAS1069 | F-CCAACCATTCTGTAATGAACTGTG | 58 | 223 | Chr.1(1,688.8) |
|  | R-GGTACAAGAAGCCTGACAAAGAAT |  |  |  |
| SICAAS1034 | F-TCAGCGAGCAGTAGTTCAACCAGC | 59 | 137 | Chr.1 (2,404.1) |
|  | R-CCCGGCGACGTCCTTCACGA |  |  |  |
| SICAAS1004 | F-TCGATCTGTAAGCAGGTTTCG | 51 | 113 | Chr.1 (2,417.9) |
|  | R-AGTTGCCATATTGCATGTGCT |  |  |  |
| SICAAS1035 | F-TCAGGAGAGCTCGCTTTGACCTGT | 59 | 223 | Chr.1 (2,727.0) |
|  | R-GCCTGCTTCTCTCTTGGTAGTTGGGT |  |  |  |
| SICAAS1039 | F-TTTCCGCTCCCAAACCAAGTCGT | 57 | 229 | Chr.1 (4,314.1) |
|  | R-ATCGGGGGATCCCGGACTCGT |  |  |  |
| SICAAS1037 | F-CCCTGAACTCTTTCTTCGGGTGGGA | 59 | 178 | Chr.1 (4,540.5) |
|  | R-ACCGTAGAGCAGAACGCCCTGA |  |  |  |
| SICAAS1038 | F-TACCATGGTGTCAAGCATGATGAAGGC | 59 | 194 | Chr.1 (4,613.8) |
|  | R-GCATTGTTTCGTACAGGCTGGAGAACT |  |  |  |
| SICAAS1005 | F-CCACTAGCTACCATCAATTCTTTTC | 51 | 147 | Chr.1 (4,725.2) |
|  | R-TGAGCAAACCATAACAACTCCA |  |  |  |
| SICAAS1042 | F-TCCTCCCGAAGTGTCAACCTCGT | 59 | 166 | Chr.1 (5,357.0) |
|  | R-CAGCAGCTCTGCATTTGCATCGTC |  |  |  |
| SICAAS1043 | F-GCTAGGCATGACTCACCTGCTACTGA | 60 | 164 | Chr.1 (5,452.1) |
|  | R-GGGGGTTGGAGCCTGGAGGA |  |  |  |
| SICAAS1045 | F-TGTGGTACACGATCAACGGCGAC | 59 | 161 | Chr.1 (5,555.5) |
|  | R-TCCCGCTCACGACCGAGAACAT |  |  |  |
| SICAAS1006 | F-ACAGTCTGCACAAAGCAAGAAT | 51 | 142 | Chr.1 (6,158.8) |
|  | R-GAAGTCCAAAGAATGCAATGAG |  |  |  |
| SICAAS1065 | F-ATGCATGCTCTGGTTGTGAC | 57 | 186 | Chr.1(6,714.7) |
|  | R-CTGCGAGCAGGTGACAGAT |  |  |  |
| SICAAS1007 | F-GCTAAGAAAGTGGGGAGCACT | 53 | 189 | Chr.1 (7,131.5) |
|  | R-CCAAACACACCCTAAATGACCT |  |  |  |
| SICAAS1059 | F-AAAAATCGGGCAAGACCAC | 55 | 233 | Chr.1(7,177.8) |
|  | R-GGGTTCTTGCTTCTGTCACC |  |  |  |
| SICAAS1046 | F-TGTGGCTCTACAGCTCTGCATGGT | 59 | 185 | Chr.1 (7,189.0) |
|  | R-TGATGGTTGGTAGTACTTCCCGCATGT |  |  |  |
| SICAAS1008 | F-GAGACGAAGAATGAAAGGACGA | 53 | 145 | Chr.1 (8,163.3) |
|  | R-CGGGGCACCGTTAAAACTA |  |  |  |
| SICAAS1009 | F-ACACCCTTCCCGAATATACAGA | 51 | 125 | Chr.1 (9,007.7) |
|  | R-TGCTGTATCATCTTCCTTACCA |  |  |  |
| SICAAS1066 | F-ACACGTATGCACGAGGTTTG | 57 | 215 | Chr.1(9,015.2) |
|  | R-GCTGCTGGGAACTTCTCTGA |  |  |  |
| SICAAS1010 | F-TGTTTATTTTCCCAGCCTCA | 49 | 185 | Chr.1 (10,619.2) |
|  | R-AAAAGTCAATGGCATCATCG |  |  |  |
| SICAAS1011 | F-CCCTTCCCTCCCTTTCTTCT | 55 | 198 | Chr.1 (10,619.7) |
|  | R-CGCCTACGTAGCTGTTTCCT |  |  |  |
| SICAAS1012 | F-ATCGAGGGAAGAAGCTGCTAAT | 53 | 142 | Chr.1 (10,691.6) |
|  | R-CAGACAACCCCCTGTACAAAGT |  |  |  |
| SICAAS1013 | F-CATCTAGCACGATATCAGGTCCA | 53 | 196 | Chr.1 (10,748.3) |
|  | R-ACCCTATGCATGGAATAGAAGGT |  |  |  |
| SICAAS1015 | F-CATGACATGTGTCAGACTAAACACT | 51 | 196 | Chr.1 (11,772.5) |
|  | R-TGCAGCATTCTCTGTTCTTTC |  |  |  |
| SICAAS1016 | F-GCGCCTTTCTCAGGAACA | 52 | 180 | Chr.1 (12,411.4) |
|  | R-CCAGGTCCAATTTGTGATAGC |  |  |  |
| SICAAS1055 | F-AAGAGGGTCTGACGAAGCTG | 59 | 191 | Chr.1(12,968.7) |
|  | R-CTGTTCCACGGATAGGGAAG |  |  |  |
| SICAAS1070 | F-TAACACTGCACCGGAAGATG | 57 | 255 | Chr.1(13,329.3) |
|  | R-GGCGGACAACCAAGCTACTA |  |  |  |
| SICAAS1047 | F-GTGGCAATCAAGTGAACCTCGGGA | 59 | 248 | Chr.1 (14,532.3) |
|  | R-AGCAGCAGATATGGCGCTACCAC |  |  |  |
| SICAAS1017 | F-GCCTTGGCTGGTGATCAATTA | 53 | 111 | Chr.1 (15,201.7) |
|  | R-TAAAACCACACCCCAGAGAAAC |  |  |  |
| SICAAS1018 | F-CGAGCATATATGTGAGGATGTG | 53 | 134 | Chr.1 (16,119.8) |
|  | R-AGGGGATTGCCGCCTAAG |  |  |  |
| SICAAS1019 | F-GCGCTTAATGCCCGTAAATA | 51 | 164 | Chr.1 (19,718.7) |
|  | R-TGCTGGGTCAAATGAGAAGA |  |  |  |
| SICAAS1020 | F-AGGGGTGAGTGGATCTACGTG | 57 | 118 | Chr.1 (19,866.1) |
|  | R-ACTTCTCCCTCTCCCTCAGAC |  |  |  |
| SICAAS1021 | F-CCTTAAGCCATGTCGTGATGT | 53 | 172 | Chr.1 (21,236.4) |
|  | R-GAGTCGAACCGTGCAAGC |  |  |  |
| SICAAS1049 | F-TTGAGGCCACCAAATTTATGTGCAAGC | 57 | 166 | Chr.1 (26,457.3) |
|  | R-GGCGACTAGAAGCCATATGGGCTAAAC |  |  |  |
| SICAAS1022 | F-GGCACGGACGGCATGTGAATCT | 59 | 142 | Chr.1 (26,702.9) |
|  | R-AGGTCATGTCCGGCATCCAGCA |  |  |  |
| SICAAS1023 | F-CCCCCTTTTCCTCTTCGAGCCTTG | 60 | 144 | Chr.1 (28,410.5) |
|  | R-GGGGCCTCACACCCTCCTCTTA |  |  |  |
| SICAAS1058 | F-GATGCACCTTCTTCTTGACGAG | 60 | 136 | Chr.1(28,503.0) |
|  | R-GCCGAGGAGAGGAAGAGAAAG |  |  |  |
| SICAAS1050 | F-GATCCGACGTGGAGCGAGCG | 59 | 112 | Chr.1 (28,661.1) |
|  | R-TGCCTCCCACGAGTCAGCCT |  |  |  |
| SICAAS1024 | F-TCGCACACGGCACATCCACATC | 59 | 148 | Chr.1 (29,600.1) |
|  | R-GCACACGTAACCCTCTGCTCGC |  |  |  |
| SICAAS1071 | F-TTGCGATGGGATACAGATGA | 55 | 190 | Chr.1(31,968.0) |
|  | R-CAAAAGCCCTTGAACTGGTG |  |  |  |
| SICAAS1061 | F-CAGAGCCGAAGGAAGAACAG | 57 | 167 | Chr.1(32,041,6) |
|  | R-TCGTGCTTTGTGGTAGGATG |  |  |  |
| SICAAS1025 | F-AGCCATTCTGTTGGACGGTGTCAG | 59 | 240 | Chr.1 (32,194.9) |
|  | R-GCCTGACTCAACGGAACATGAGCA |  |  |  |
| SICAAS1060 | F-AGAAGGGGAGGACAAGGGTA | 57 | 158 | Chr.1(33,693,7) |
|  | R-ATCGGTCAATACGGGTGAGA |  |  |  |
| SICAAS1062 | F-CCCAGCCCATAATCTGAAAC | 57 | 223 | Chr.1(35,085.9) |
|  | R-TGCACTGCTGAAGGAAACTC |  |  |  |
| SICAAS1056 | F-TGTTTGTCTGCATCGGGATA | 55 | 153 | Chr.1(35,353.1) |
|  | R-GCAGTGACGGCTTCCTCTAC |  |  |  |
| SICAAS1026 | F-ACCGGGTCTTGTACCGTGTGTCT | 59 | 187 | Chr.1 (36,262.3) |
|  | R-GCCGGAATAATGCTTGCTAGTGGGC |  |  |  |
| SICAAS1027 | F-TGGGTTCCTTGTCGGCCTCGT | 59 | 197 | Chr.1 (36,973.9) |
|  | R-AGCCCAGCTGTCTGACAAGAAGGT |  |  |  |
| SICAAS1063 | F-GGAATGAAGGTTGAGCAGATG | 55 | 202 | Chr.1(37,314.9) |
|  | R-ATCTTTGGGGAGGGTTTTTG |  |  |  |
| SICAAS1064 | F-GACCATCCCTTGTTTGCTTG | 57 | 180 | Chr.1(37,810.8) |
|  | R-TAGCCCTCACATCCTGCTCT |  |  |  |
| SICAAS1028 | F-AGCCGTCTCTTTCCTCGCTCCT | 57 | 195 | Chr.1 (38,010.1) |
|  | R-TTGCTTTGCGTTGCGTCCACCA |  |  |  |
| SICAAS1072 | F-AACTAATCAAGCTCTCTTTGAGCAC | 58 | 151 | Chr.1(38,892.8) |
|  | R-GACGGTGAGATGACTGGTAAGAC |  |  |  |
| SICAAS1029 | F-CCTCTGCGCCCTGAATGGATCG | 59 | 108 | Chr.1 (39,481.7) |
|  | R-ACTACCCCTCTCGCACGTTGACT |  |  |  |
| SICAAS1057 | F-CTGGAACACCAAGCTGAAGA | 57 | 278 | Chr.1(40,006.1) |
|  | R-CGTTGTTGTCGTTGTTGCTG |  |  |  |
| SICAAS1051 | F-GGCATAGCAATAGCATGCAGGGATGT | 59 | 177 | Chr.1 (40,494.2) |
|  | R-AAGCAGTACTCGTCAACACAGGGC |  |  |  |
| SICAAS1030 | F-GGCCATGTCTAGGCTGCTAAGGTCTA | 59 | 150 | Chr.1 (40,749.9) |
|  | R-GCAGAAAGCATGCCGTAGTCAAACG |  |  |  |
| SICAAS1031 | F-TGGTCTCACCCGGTGGTTGGAT | 59 | 148 | Chr.1 (40,870.6) |
|  | R-GGACGGACAGAGCCATGTGTGC |  |  |  |
| SICAAS1032 | F-CGACGTTGCCGTTGCCCATCAT | 59 | 146 | Chr.1 (40,962.8) |
|  | R-CCTGCTGCCAATCCAGCCCAG |  |  |  |
| SICAAS1052 | F-CCTCCCACCCTTGCCTCCCA | 59 | 112 | Chr.1 (41,800.8) |
|  | R-AGGGAGAGATTGCCGGAAGAGCAT |  |  |  |
| SICAAS1053 | F-ACGCCGCGGTGATGTGGTAAAT | 57 | 140 | Chr.1 (41,802.1) |
|  | R-GGTAGCAGCTGTGCTGGCCTTG |  |  |  |
| SICAAS1054 | F-CCCCCACTCAAACACCATTACAGCA | 59 | 146 | Chr.1 (41,871.5) |
|  | R-GGTTGGTGTCACCGCCTTTAATCATCA |  |  |  |
| SICAAS1033 | F-ACATGGCCACGATCTTGCTTGCT | 57 | 169 | Chr.1 (41,897.7) |
|  | R-CTCCCCTCCCCTCCCTCTCCAC |  |  |  |
|  |  |  |  |  |
| SICAAS2082 | F-ACAGGGTCCAGTCAACTGCT | 56 | 234 | Chr.2(81.9) |
|  | R-TTTTTGTGAACGCAGCAATG |  |  |  |
| SICAAS2085 | F-GTATCATCGGGCTACGTGAGA | 58 | 278 | Chr.2(124.9) |
|  | R-ACACGTAGTCGGAGCAACAAT |  |  |  |
| SICAAS2057 | F-TCGCGAGCTCGTTCCACAATCCGCA | 60 | 256 | Chr.2 (312.0) |
|  | R-ATTGCTCACCCACGCTGGCATCCT |  |  |  |
| SICAAS2034 | F-TCGCGTCCATGGCATGCGGCTCAAAGA | 62 | 191 | Chr.2 (585.6) |
|  | R-TTGGTGCGACGAGAGCAGGAACCCT |  |  |  |
| SICAAS2026 | F-ACATGGTCCGCACCGCACCTAGAGA | 61 | 199 | Chr.2 (918.7) |
|  | R-TCCGATGCTCGCTCACTCCACCTTGA |  |  |  |
| SICAAS2027 | F-TCATCCGCTCCGCCTCCCTCATGTT | 61 | 111 | Chr.2 (1,605.8) |
|  | R-GCTTGCCGGTGAGAGCACAAGGAGCTA |  |  |  |
| SICAAS2001 | F-AGAATGCCCGGCGTTTCGCGTCAT | 60 | 208 | Chr.2 (1,738.5) |
|  | R-TGGCCCCCTTAGACCTGCTTCTGCT |  |  |  |
| SICAAS2028 | F-TTTGGTACGTGACACCCGGCGCAT | 60 | 111 | Chr.2 (1,739.7) |
|  | R-ATCATGTTGGAGGGCTGCGTGCGTGCT |  |  |  |
| SICAAS2029 | F-TCGGCCAATGAAGTCGCCAACCGCCAA | 60 | 135 | Chr.2 (1,835.8) |
|  | R-TGGGCGTGTAATTTTGAGCGCGGCA |  |  |  |
| SICAAS2002 | F-TTGGCCAGCACGTACGACCCACACT | 62 | 232 | Chr.2 (1,936.1) |
|  | R-TGGATGGGTTGGGTGGCGCACAAGA |  |  |  |
| SICAAS2075 | F-GTGAGAAAGGGAGGAAAAGGAC | 58 | 235 | Chr.2(2,107.8) |
|  | R-GACAAAAACTGGGACCGTTAGA |  |  |  |
| SICAAS2003 | F-TTGCGAATGCACGGGTCAGCGACT | 60 | 224 | Chr.2 (2,422.3) |
|  | R-TGTATGCCACTCGCACGGCTACCACCT |  |  |  |
| SICAAS2004 | F-TGTGTTGCGCTTGATGGTTACCAGCGT | 60 | 252 | Chr.2 (2,504.3) |
|  | R-ATCGGGGAGGGCCCAAAACAGAGGA |  |  |  |
| SICAAS2076 | F-TTGACCACATTCATGGCCTA | 55 | 173 | Chr.2(2,729.3) |
|  | R-TGCGATTTTCGTGTCTTGTG |  |  |  |
| SICAAS2073 | F-TTCTCTGGGATGTGCTATCG | 59 | 207 | Chr.2(2,952.6) |
|  | R-ATGCTCCAGACAGGCTCAGT |  |  |  |
| SICAAS2035 | F-TGCTCCTTGCTGATCATTGCTGGGGC | 61 | 145 | Chr.2 (3,114.7) |
|  | R-TGCTGGCTCACCTTGCTTGCTCCTCCT |  |  |  |
| SICAAS2005 | F-AGGGCACCGTGCGTTTATCAGGGGA | 62 | 146 | Chr.2 (3,488.6) |
|  | R-ACGCGACGAAAAACAGAGGGAGGGAGG |  |  |  |
| SICAAS2081 | F-CCTTGAAATGGAGGATTTCG | 55 | 202 | Chr.2(3,914.0) |
|  | R-CATGTCCCTGTTGGACTGGT |  |  |  |
| SICAAS2036 | F-TCGATTACGGTCCCGGTCCCTCTCCAT | 62 | 203 | Chr.2 (3,989.8) |
|  | R-TGTATGAACACGCCTGTGCGCCGAC |  |  |  |
| SICAAS2072 | F-CTAGTGTCCCATTTTAGAGCACAT | 58 | 225 | Chr.2(4,080.8) |
|  | R-ATTTCTTCTAGTGCCTGGTTTCAC |  |  |  |
| SICAAS2006 | F-AACCCCCTTCGTCCTCCTCACTCCACT | 62 | 240 | Chr.2 (4,743.5) |
|  | R-TGAACATCGCTGCCAGCCTGCCACT |  |  |  |
| SICAAS2037 | F-AGGTGCCGGCTGGTGTTTCTTGGCA | 62 | 156 | Chr.2 (4,895.9) |
|  | R-ACAAAGCGGGCGCAATCCTCCTGT |  |  |  |
| SICAAS2038 | F-ATGGAACACACTGCGCACACGCCA | 60 | 318 | Chr.2 (5,138.8) |
|  | R-AGTTTGCTCCTCGGCTCGGCTTCCA |  |  |  |
| SICAAS2007 | F-TGCCACCGAGAGCAACCTGCGAAGT | 62 | 175 | Chr.2 (5,378.3) |
|  | R-AGCTCCGTCGTCGTCGATTCCGACCAT |  |  |  |
| SICAAS2039 | F-GGTGGAACTGCACGGGGAAGGATTGGA | 61 | 242 | Chr.2 (5,494.6) |
|  | R-TGCTGCACGCGCTCTATCTCAGTTGGT |  |  |  |
| SICAAS2040 | F-ACGTTTCAGCTCTTGGCGACTTGGCGT | 61 | 185 | Chr.2 (5,678.2) |
|  | R-ACTCCGAGCTGCATGTCTTTGGCCGGT |  |  |  |
| SICAAS2083 | F-ATGTCATAGGGGAGCGAGGT | 51 | 178 | Chr.2(5,773.9) |
|  | R-ACAAGCAGAAGCAGGGAGAG |  |  |  |
| SICAAS2058 | F-TCCCACCTTGGCCCAGCAACAACCT | 62 | 213 | Chr.2 (6,268.9) |
|  | R-AATGCGGAAGCGGTTGCGTGGCAGACT |  |  |  |
| SICAAS2059 | F-ACGACACACACAAGCGGGCACACAA | 60 | 193 | Chr.2 (7,128.2) |
|  | R-TGCGCCAAATCCCCTCCAATGAACCCC |  |  |  |
| SICAAS2074 | F-ATGGTGTGCATTGGTCTGAATA | 53 | 202 | Chr.2(7,267.4) |
|  | R-CTTGTTTTGGGAAACTTGTGCT |  |  |  |
| SICAAS2077 | F-GACAAAACAGAGAAAGCTGTGC | 58 | 151 | Chr.2(8,034.5) |
|  | R-GCCTAGCACGAAACAGGAAATA |  |  |  |
| SICAAS2078 | F-AGAAGAGCTATCCCGCCACT | 58 | 159 | Chr.2(8,034.8) |
|  | R-TTTCTCTTCATCCTCCTGCTG |  |  |  |
| SICAAS2041 | F-ACCACCCTCCCCACTCACTCGCAAT | 60 | 279 | Chr.2 (8,102.5) |
|  | R-ACGCGTTGGCCACGTTTGAGCGAT |  |  |  |
| SICAAS2047 | F-TTGGGGGTGTAGCCAGGATCAGCCA | 62 | 240 | Chr.2 (8,539.1) |
|  | R-AGGCCGGCATGCGATGTGATTCGGT |  |  |  |
| SICAAS2009 | F-TTGGGGGTGTAGCCAGGGATCAGCCAT | 63 | 243 | Chr.2 (8,546.4) |
|  | R-ATGCGATGTGATTCGCTTCACGGGGCG |  |  |  |
| SICAAS2060 | F-ACCCCGCCACGTATATAGCACCAGCA | 61 | 244 | Chr.2 (8,717.4) |
|  | R-AGTAGTAGAACTGCTGCACGACGGGGC |  |  |  |
| SICAAS2079 | F-GGCCTACAAGCAGAGAGACG | 59 | 265 | Chr.2(8,794.1) |
|  | R-GCAAGGAGGAGGAAAGGTG |  |  |  |
| SICAAS2010 | F-GGCGTCACGCAACCGAACACACACA | 62 | 120 | Chr.2 (10,073.8) |
|  | R-TTGACCCACCGCACCCTCTACACCT |  |  |  |
| SICAAS2086 | F-ACTGCTTCGTCTTTCGCCTA | 57 | 230 | Chr.2(10,794.5) |
|  | R-AGCCACCTGTCACCATTTTC |  |  |  |
| SICAAS2048 | F-TCAGGGCAGTGAGTTGCTGCCAATGT | 60 | 199 | Chr.2 (11,688.3) |
|  | R-AGCAGCCCACCAGATGGACACGTGGAT |  |  |  |
| SICAAS2061 | F-GCGTGAGGACGAAGAGCTTGCACGAGT | 63 | 215 | Chr.2 (13,679.7) |
|  | R-TGGAAGGTACGCATGCATTGCTCCGGC |  |  |  |
| SICAAS2062 | F-AGCGCGGCACTGCACGTTGGATAGCAT | 61 | 243 | Chr.2 (15,020.7) |
|  | R-TCGCAATGTGGTGGGATCGAGCTGGT |  |  |  |
| SICAAS2011 | F-CCGCGAGCTCCAGAAGGTCTTGCTCTT | 63 | 242 | Chr.2 (15,129.8) |
|  | R-TGCCCTCCTTTCTGCCTGCTCTTGTGC |  |  |  |
| SICAAS2063 | F-AAGAAGAAATTCGCCGACCGGAGCCC | 61 | 237 | Chr.2 (16,389.7) |
|  | R-ACCGTCTTATCTGCACGCCTCCCTCT |  |  |  |
| SICAAS2064 | F-TCGCTTCCGTCCTTTGTTCCCGCACT | 61 | 119 | Chr.2 (21,103.9) |
|  | R-TCGGGTGCACCATTTGATCCGGCCA |  |  |  |
| SICAAS2049 | F-GGCGCAAGCTCACCAAGGACTGTCT | 62 | 260 | Chr.2 (23,882.9) |
|  | R-CGGTCGTTGCGCGTCCAAGAAAAACCG |  |  |  |
| SICAAS2084 | F-TGTGGCCTAGATCCTGAATATCAT | 58 | 172 | Chr.2(24,188.0) |
|  | R-GTCCCTGGCCTTGAAGTAAATAG |  |  |  |
| SICAAS2065 | F-TGCCAGCAGAATGGCAGATGCCAAAGA | 60 | 202 | Chr.2 (25,296.7) |
|  | R-AAGGGAGGTGGGGAAGAGGGGAAAAGG |  |  |  |
| SICAAS2066 | F-ATCATCGAGATCAACGGGCCGTGCTGC | 62 | 195 | Chr.2 (27,513.7) |
|  | R-ATGCATGCCTCCGGGGGTCACATCT |  |  |  |
| SICAAS2050 | F-ATCAGGCTAGCCCGCAACCAACCGA | 62 | 210 | Chr.2 (28,406.5) |
|  | R-ACGGTTCACGCGCACGAGGAAGAAG |  |  |  |
| SICAAS2042 | F-AGGCGCGCATTGGCATTGTCATCCCGT | 63 | 155 | Chr.2 (30,532.0) |
|  | R-TGCGGTCGTTGTTGTACAGGGGCGCAT |  |  |  |
| SICAAS2030 | F-AGCTAGCTATTGGTACCGCACTCCCCA | 61 | 269 | Chr.2 (31,530.9) |
|  | R-ACGATGCGCATTGGTAGCGACGGATCT |  |  |  |
| SICAAS2043 | F-ACTGTTGCGGCTCAGGCGATGGCACTA | 62 | 306 | Chr.2 (32,764.7) |
|  | R-ATGCGCGCGTCCGCATCTGTTTCCA |  |  |  |
| SICAAS2015 | F-TCCATCACCGCCGGACCATGAACCA | 62 | 217 | Chr.2 (33,576.5) |
|  | R-TCCCCCTCCACTCCATTTGCTGCCCTA |  |  |  |
| SICAAS2051 | F-TCCACGACTCCAAGTCACCTCCTCCCT | 63 | 195 | Chr.2 (34,649.9) |
|  | R-GGCTTGCTTCTTTTTCCTCCCGCCGGT |  |  |  |
| SICAAS2067 | F-TCGTCCGAGCATCCGCAAAGGGAGA | 62 | 232 | Chr.2 (35,068.8) |
|  | R-TCCATCAGCAGGCCGTAACAGTGGC |  |  |  |
| SICAAS2031 | F-AGCAGCCACAGGTAAGGCGTGCTTG | 62 | 303 | Chr.2 (35,638.3) |
|  | R-AGCTGCATGTGCCCGCGGCAGAATAGT |  |  |  |
| SICAAS2044 | F-AGGACGAAATCGCAGCGGATGGGGA | 62 | 217 | Chr.2 (36,136.4) |
|  | R-CGCGGCGTGAACTTGGCATGAACGA |  |  |  |
| SICAAS2032 | F-TCCTGAGCTCGATCATCTGCCGTCCCT | 62 | 186 | Chr.2 (36,366.7) |
|  | R-AGCTGCCTGCGCAGTCTGGATGTCT |  |  |  |
| SICAAS2068 | F-TGATTCAGCAGCAGCACGCCACTGT | 60 | 161 | Chr.2 (36,780.2) |
|  | R-TCGGACCGATCCAGCTACCACACCA |  |  |  |
| SICAAS2012 | F-TTGTGTATGGCGGCGGCGTGTTTGC | 62 | 163 | Chr.2 (36,917.8) |
|  | R-AGCGACATTGCCGTTCACCTTCCGCT |  |  |  |
| SICAAS2013 | F-TTAACGGCGGGTGTGGGCGTGAGATGT | 63 | 135 | Chr.2 (38,090.6) |
|  | R-TGCTGGAGCCCGCCACCAACTGTAGTA |  |  |  |
| SICAAS2052 | F-ATGCCCTCTCCTCTCCTCCCCACTCTA | 62 | 221 | Chr.2 (38,184.2) |
|  | R-GGCATGGTGTGCGGCACTGTTTCCA |  |  |  |
| SICAAS2045 | F-ACCGGGTGCTCACAGGAAAATGGCCGA | 63 | 174 | Chr.2 (38,195.3) |
|  | R-TTCGCGGCACGGCGCAACAGGGTTTTA |  |  |  |
| SICAAS2014 | F-CGGTTGGTGCGCCTCATTAACCCCCTT | 63 | 149 | Chr.2 (39,121.6) |
|  | R-TTGCAGCAGTAAGCCAGGAACGGCCAC |  |  |  |
| SICAAS2046 | F-GCCACGCACACCAACTTGCTTTTCCCT | 61 | 228 | Chr.2 (39,143.3) |
|  | R-AGAACACGCACACCCGCCTGAACCT |  |  |  |
| SICAAS2080 | F-AAATTGCACCCATGCTGGTA | 53 | 188 | Chr.2(39,508.9) |
|  | R-TGCAAAGGAATGGAAACACA |  |  |  |
| SICAAS2071 | F-TGAGGGAAGTTGGGATTCTG | 55 | 160 | Chr.2(39,890.1) |
|  | R-TTGGTATGGGAGCCACAAAT |  |  |  |
| SICAAS2016 | F-TTGCGCTGTTGCGATCTGCCGCTCT | 62 | 248 | Chr.2 (41,256.3) |
|  | R-ATGGCGTCCATTCCTCTCCGCTGGT |  |  |  |
| SICAAS2017 | F-GGCGCCATGGGTGGATGCGGAATTGTA | 63 | 136 | Chr.2 (41,333.1) |
|  | R-TCAGTTGTCTCCCTCGCCGCTTCAGGA |  |  |  |
| SICAAS2018 | F-ACGAGCACGGGTTTACGAGACCCGACA | 62 | 141 | Chr.2 (41,432.2) |
|  | R-AGCTAGCTGCCACTGGGTGGGTAGT |  |  |  |
| SICAAS2019 | F-AGGGAAGTGACCGCCACCAACAGTGA | 62 | 261 | Chr.2 (41,437.1) |
|  | R-GCTTGTTGTGCAGCGGTGCCAGCAGTA |  |  |  |
| SICAAS2033 | F-AAGCTGGCAACAAGTACGCCGGCCACA | 63 | 198 | Chr.2 (41,537.6) |
|  | R-ATAACTTTCCGCGGATGCAGGCGGCAC |  |  |  |
| SICAAS2053 | F-ACAAGGGTTGTCGGCCGGTAAGGGT | 61 | 244 | Chr.2 (42,098.3) |
|  | R-TTCCGATCAACGCCGTGCAACGCCTA |  |  |  |
| SICAAS2069 | F-TGCTTCTGTGGCCGCCGATACAGGT | 62 | 292 | Chr.2 (44,845.8) |
|  | R-GGCACTCCTACAACACCACGTGCTCCA |  |  |  |
| SICAAS2020 | F-TCGTAGCACCCACTGCACGCTCCTA | 62 | 320 | Chr.2 (45,565.5) |
|  | R-CTCACCTGTTCCCGAATCAACCTGCCG |  |  |  |
| SICAAS2021 | F-TGTTGCCTCGCTCTCGTGACACCCA | 61 | 272 | Chr.2 (46,087.6) |
|  | R-GGCCTAACCTGGTCTTCGTTTTGGCCT |  |  |  |
| SICAAS2022 | F-TGTGTACGGAGTATGTGCAGGCTGGCG | 63 | 152 | Chr.2 (46,198.9) |
|  | R-TGGGCTATCGACGACACCGAAACGGGA |  |  |  |
| SICAAS2023 | F-CGGCCGCGGCAGGCATGATTGATTGAT | 62 | 168 | Chr.2 (46,207.3) |
|  | R-AGGCCCTGCATCCAGCTCAGCACAT |  |  |  |
| SICAAS2054 | F-AGCCAGCACAGTAGAGAGGAGGGCA | 62 | 226 | Chr.2 (46,349.7) |
|  | R-GCCTGCGGTTTGGTGCGCCACATTGAT |  |  |  |
| SICAAS2055 | F-AAAAGGCCCCCGGCTGTTTCGATTCCG | 62 | 198 | Chr.2 (46,590.4) |
|  | R-TCCAGGATGGCCCGCGTCGATCAAT |  |  |  |
| SICAAS2024 | F-CGAGTCAGAGAATGCCAGCTGACCCCT | 62 | 280 | Chr.2 (46,592.9) |
|  | R-TCAGCCAGGTACAGCGACACGACCA |  |  |  |
| SICAAS2025 | F-TCCATGTTCATGTGCTGCTCGAGCCCT | 61 | 179 | Chr.2 (46,635.1) |
|  | R-GTGTAGATGCTTGGTGCCATGCCGACT |  |  |  |
| SICAAS2056 | F-TTCCTGGCAGACGGGACAAGCCTGA | 62 | 151 | Chr.2 (47,222.4) |
|  | R-AGGCCGCCGAGGCAACAGCTTTACA |  |  |  |
| SICAAS2070 | F-TGGTCGCTTCGCTTCAGTGGACACGCT | 62 | 257 | Chr.2 (48,613.2) |
|  | R-AAAGCAATGGCGGCCGCACCAGAGA |  |  |  |
|  |  |  |  |  |
| SICAAS3054 | F-TGGGTAGAGCTGGCCTCCTGGTTCT | 60 | 191 | Chr.3 (84.9) |
|  | R-TGCATGTCCACCTGTCCAACGAGC |  |  |  |
| SICAAS3069 | F-CTGCAACCTGTCTTGAGAGC | 59 | 171 | Chr.3(137.8) |
|  | R-GCGCGATCCTACGTCTACA |  |  |  |
| SICAAS3001 | F-AGCTCGCTGGAGAAGAGGATCGGAC | 60 | 148 | Chr.3 (170.5) |
|  | R-CGTACCAGTACGGCACAGAGCTGA |  |  |  |
| SICAAS3082 | F-TGAGTTTTGAGTTGGGGTCAC | 56 | 226 | Chr.3(347.4) |
|  | R-ATAACATAATCCAAGCCGCAGA |  |  |  |
| SICAAS3002 | F-TGTGGTGTGCGGCTCATATCCGT | 59 | 288 | Chr.3 (358.8) |
|  | R-CTGCCGCCTAATTGTCGTGTCGTCT |  |  |  |
| SICAAS3055 | F-ACGTTGCGCATCTCTCCCTCCA | 59 | 134 | Chr.3 (370.9) |
|  | R-TGTGCGCCTGTGGAAGACACTCA |  |  |  |
| SICAAS3003 | F-CCGGTAAGAGGGAAAGTGACGGGGA | 59 | 156 | Chr.3 (507.6) |
|  | R-AGCTGCACACACAGGCGTATCGT |  |  |  |
| SICAAS3056 | F-TGCGCAGGCAGCTTGTTGTTGT | 57 | 136 | Chr.3 (518.2) |
|  | R-AATCGGATTTCCCTCCGCCCGT |  |  |  |
| SICAAS3083 | F-CGTGATCGGAGAGAAACAGAG | 56 | 232 | Chr.3(529.7) |
|  | R-ATGTTCATGTTGGGCATGTTG |  |  |  |
| SICAAS3084 | F-GGAGCAACAACTATGGTGATAAAAG | 58 | 146 | Chr.3(900.6) |
|  | R-TAGCAATGGTCTAAAGAGGAAGGA |  |  |  |
| SICAAS3004 | F-ACTGCGAAAGGGGATGCAGCAGA | 59 | 124 | Chr.3 (1,001.1) |
|  | R-TCTGTGGGCCGTTGCTCTCACT |  |  |  |
| SICAAS3072 | F-AGCATTAGCTGAGACCCGATAC | 60 | 221 | Chr.3(1,056.6) |
|  | R-GATTGCAGATAGTCCCAGCTTC |  |  |  |
| SICAAS3088 | F-GCATACCACCAACCTCCAAC | 59 | 201 | Chr.3(2,132.4) |
|  | R-CTGCGTGAATGAGTGGACTG |  |  |  |
| SICAAS3005 | F-TGCCTCACAAACGCGAACGGCT | 59 | 192 | Chr.3 (2,612.8) |
|  | R-GCCTGCAGTTATGCGCCTCCATAC |  |  |  |
| SICAAS3057 | F-ACACACATGCCCACCACCACCA | 59 | 196 | Chr.3 (2,659.5) |
|  | R-GCCATTGCCACATCTCTGTGCTCTC |  |  |  |
| SICAAS3006 | F-CCGCCGATCTCACATGCGGAACAT | 59 | 291 | Chr.3 (3,308.4) |
|  | R-TGGGACGTCGCTGTTATGACCGT |  |  |  |
| SICAAS3007 | F-TTTTTGTGGCTTGTGGCCGTACTAGC | 59 | 230 | Chr.3 (3,479.2) |
|  | R-ATTGGTCCAACTCCTCCTGCCGTC |  |  |  |
| SICAAS3008 | F-ATAGGCCCACCCCTCCCTAATCCCT | 59 | 213 | Chr.3 (3,580.1) |
|  | R-TCATCACCAGCCCAGGCCACAT |  |  |  |
| SICAAS3009 | F-TTGTGCCTTTGTGCCCTGCCCT | 59 | 132 | Chr.3 (3,580.4) |
|  | R-GGATTGGGTTGGCGATTGGACGGT |  |  |  |
| SICAAS3087 | F-GAAGAGAGGGAGTGGTTCCAT | 58 | 192 | Chr.3(3,924.7) |
|  | R-CTAGCATTTAGCAATCTGCCATC |  |  |  |
| SICAAS3010 | F-TGCGTGTGGCGATGTGAGATGGA | 59 | 124 | Chr.3 (4,137.9) |
|  | R-ACCATCGGTTTCGTGGCTTGGC |  |  |  |
| SICAAS3058 | F-ACCACCACTCGCAGGGACAACAGT | 60 | 158 | Chr.3 (4,621.8) |
|  | R-TCCCCATGTGCCCTCATCATCAGC |  |  |  |
| SICAAS3078 | F-CCATCAACAAGCAGTCAAAGG | 56 | 248 | Chr.3(4,726.1) |
|  | R-TTCTGATTCGGTTCCTTCTCA |  |  |  |
| SICAAS3011 | F-CGCTGTTCGGAGAGGAATCTGGGCT | 62 | 166 | Chr.3 (5,119.5) |
|  | R-TGAGAGGAGAGGGGGAAGGAAAGGGT |  |  |  |
| SICAAS3079 | F-TGTGCAGGGCTAAAGAGACC | 56 | 243 | Chr.3(5,128.0) |
|  | R-TGGTTGAAAAAGAGGGTCCTA |  |  |  |
| SICAAS3059 | F-AACTCGAGGTGATCCCTCCCTCCCT | 60 | 135 | Chr.3 (5,743.8) |
|  | R-GCAATCCATTGGTCACCACCTGCG |  |  |  |
| SICAAS3012 | F-GGCCATGGCTAAGAAGGACTCCAAGC | 60 | 225 | Chr.3 (5,776.8) |
|  | R-CCGATCCCATAAGCTCCAGCACCA |  |  |  |
| SICAAS3013 | F-GATCGATGGTCCCACCTTGATGCCA | 59 | 198 | Chr.3 (5,777.6) |
|  | R-ACGACTCATGCCGACGTCGTTACA |  |  |  |
| SICAAS3060 | F-GTGGTTCAACTGAGCTTCCTTCCCCA | 60 | 177 | Chr.3 (5,801.3) |
|  | R-GCACGTCAAGCCTTGTCCACCACA |  |  |  |
| SICAAS3014 | F-TGGTTTGAGGTGGATGGGCCGGTA | 57 | 169 | Chr.3 (5,802.5) |
|  | R-TGTTGCCGCAAGCAAGCAAGGA |  |  |  |
| SICAAS3085 | F-GAACAAGCCCAAGCTCAAAC | 57 | 247 | Chr.3(6,266.0) |
|  | R-ACCGTGGGGACATCTTCAT |  |  |  |
| SICAAS3015 | F-AGAAAGCCGGTGGTTCGTTGCG | 59 | 263 | Chr.3 (6,284.9) |
|  | R-GCCTGTGTGGTTGGTTCGTGCT |  |  |  |
| SICAAS3016 | F-GCAGTGGCTGCCCAGTTCATTCCA | 59 | 196 | Chr.3 (7,329.8) |
|  | R-TGTTGCTTCCTACCAGGACAAGCCA |  |  |  |
| SICAAS3077 | F-CCTGAACGAATAACGTGCAA | 55 | 250 | Chr.3(7,364.3) |
|  | R-GCCAACAAAAATGACGGAAC |  |  |  |
| SICAAS3017 | F-ACGTCCCGCACCTCACCGACTATCT | 62 | 163 | Chr.3 (7,414.9) |
|  | R-TTGCAGGGGATTCGGGTAGCTCTGC |  |  |  |
| SICAAS3070 | F-AAAGGCGTGGAATCACAAGT | 55 | 183 | Chr.3(7,659.4) |
|  | R-GAACTACCCCGCAAGACATC |  |  |  |
| SICAAS3086 | F-CGAGCAAACAATCTCAGCAG | 57 | 230 | Chr.3(10,457.3) |
|  | R-AGTACGGGTACGGCACGTT |  |  |  |
| SICAAS3073 | F-AAATCTTGGGGTGTGAAGTTGT | 56 | 189 | Chr.3(10,623.7) |
|  | R-TGATGACTTATGCCTAGCCTGA |  |  |  |
| SICAAS3061 | F-TGCGAATCCCACGCTGCAACTCTG | 60 | 194 | Chr.3 (11,137.4) |
|  | R-AGAGTCCTTCGGCCCATCATGGCT |  |  |  |
| SICAAS3020 | F-CGGCCGATCCAACCTCAGGTACTGT | 59 | 127 | Chr.3 (11,466.4) |
|  | R-TCTGCGACCGTTGACAAAGCCCT |  |  |  |
| SICAAS3068 | F-ACTTACCTTTTCTCTCGCATCG | 58 | 117 | Chr.3(13,148.2) |
|  | R-CTAGTGCTCCCTGTGCCTGT |  |  |  |
| SICAAS3062 | F-TGGACGTGCGTGCGAACTCTTG | 59 | 126 | Chr.3 (14,737.2) |
|  | R-AGACGTACCCTTTCCTCCCTTCGGT |  |  |  |
| SICAAS3074 | F-AAGTCGAGTCCAGCAAATCAG | 56 | 205 | Chr.3(14,789.9) |
|  | R-CATCATTCTCCCCACCTAAAAT |  |  |  |
| SICAAS3021 | F-GCACTCAGACCATGGGCATGTGAGGA | 60 | 145 | Chr.3 (15,472.2) |
|  | R-CGGCAACATTTGACCCTCCCCCAAAT |  |  |  |
| SICAAS3022 | F-GCTGCAGGCCCAGTTTAAATGCAAGC | 60 | 141 | Chr.3 (18,054.9) |
|  | R-AAAGCATGAGCAGCAGACCCGGAG |  |  |  |
| SICAAS3089 | F-CTACTCCCGTAAAAGGCCACT | 60 | 206 | Chr.3(18,107.7) |
|  | R-GCGTTATGCACTGTCACTGAG |  |  |  |
| SICAAS3023 | F-TGCAAACACACGCAGCGCCT | 57 | 121 | Chr.3 (18,850.6) |
|  | R-CGACAGATGAAGCAGTGCCCCGAAC |  |  |  |
| SICAAS3024 | F-CTAGGACCTCTCAGGTTGGTGGCGA | 60 | 132 | Chr.3 (18,874.1) |
|  | R-ACCAGCTGGATCAGGATAACCTTGCTG |  |  |  |
| SICAAS3025 | F-CCTGATGGCCTCGATCAAACTGCCT | 60 | 104 | Chr.3 (19,085.7) |
|  | R-GCAACCACATGCATCTCGTAGTAGCCT |  |  |  |
| SICAAS3026 | F-AATCGCGAGGGGATTGGCAACG | 59 | 122 | Chr.3 (19,692.7) |
|  | R-CGGCTCTTCAGTTCAGTCTCGGCT |  |  |  |
| SICAAS3027 | F-CGCATGGCCGGATTCGTTTGGA | 59 | 217 | Chr.3 (20,738.9) |
|  | R-CCACGGCACTGTTTGGAAGAGAGGA |  |  |  |
| SICAAS3028 | F-AGTCTGATGACGACGGGTCCATGCG | 62 | 196 | Chr.3 (21,172.3) |
|  | R-TGGGCATTTACCTCCCTCTCGGCCT |  |  |  |
| SICAAS3080 | F-CGGTAACTCGATAAGCTTGGA | 55 | 227 | Chr.3(22,698.5) |
|  | R-AGGGCGACCCAAATTGTTAT |  |  |  |
| SICAAS3067 | F-GACGAAGGCTATCAGAGTGGAG | 56 | 204 | Chr.3(24,603.2) |
|  | R-GATCCTTCAATTCCGTGGTTTA |  |  |  |
| SICAAS3029 | F-GGTAAGGCTTGCCATTGCCGCAT | 59 | 190 | Chr.3 (25,070.3) |
|  | R-TGGGACACCAGCAGGTTTAATGCCAG |  |  |  |
| SICAAS3030 | F-GCGTGTTGACCAAGTTAAGCGGAGGT | 60 | 187 | Chr.3 (26,858.5) |
|  | R-CGTGTAGAGCACAGTACGTGGATCGCT |  |  |  |
| SICAAS3031 | F-TCAACCCTAATGGTCCACTCAGCCAC | 60 | 195 | Chr.3 (34,293.8) |
|  | R-TGCTCCGTACCTTGGACGGCTACT |  |  |  |
| SICAAS3032 | F-ACCTACGGCTGTTCGAGAAGGATGAAC | 60 | 207 | Chr.3 (39,735.9) |
|  | R-CCCGGCAAAAACTTTGTCACACCTGC |  |  |  |
| SICAAS3090 | F-CACATGACATGAGGTAGGACTTG | 60 | 215 | Chr.3(40,543.4) |
|  | R-CTGTTGCTGAAGAGTGAGAGGA |  |  |  |
| SICAAS3033 | F-AAAAGACGGGGCGAAAGGGGGT | 59 | 176 | Chr.3 (41,073.6) |
|  | R-AACTGTTGGCCTGTTGCCGCTG |  |  |  |
| SICAAS3034 | F-GGTGCCAGACCTTGATCAACCAACCA | 60 | 193 | Chr.3 (42,252.8) |
|  | R-GAGATCCACCAAAAGTCCAACACACGC |  |  |  |
| SICAAS3035 | F-ACCGTGGAGCACCTTCGCCAACAT | 60 | 178 | Chr.3 (42,473.0) |
|  | R-AGTCGATAAGCCCTCCCTCCCTCGT |  |  |  |
| SICAAS3036 | F-AGCGACAAGGTACGCCTCCACCAT | 59 | 137 | Chr.3 (43,253.0) |
|  | R-ACTGTTGCGACATGTGAGAACTGGGA |  |  |  |
| SICAAS3037 | F-CGCCGCCGGTAAGGCATCTTCTAT | 59 | 298 | Chr.3 (43,255.2) |
|  | R-ACGCCCACGGGTGTCTTGATTTTG |  |  |  |
| SICAAS3038 | F-TGGCTGTTGCTGAACCCGTCGT | 59 | 285 | Chr.3 (43,376.1) |
|  | R-GCAGATCAGCCACCACCCAAGAAGC |  |  |  |
| SICAAS3039 | F-AAGCACACAAGCCGTGTGCACTGA | 59 | 103 | Chr.3 (44,093.6) |
|  | R-TGGTGCTGTCTGTACCTGCTCGCT |  |  |  |
| SICAAS3040 | F-TCGTGGTGCTCCTGCACGTTTAGA | 59 | 105 | Chr.3 (44,179.5) |
|  | R-ACGGGTGCTCATGGATTTGCCCA |  |  |  |
| SICAAS3041 | F-TGCTCACTAGCTAGCTCCTACAAGGCA | 60 | 242 | Chr.3 (44,320.9) |
|  | R-AAGTCACGTACATGGGTGGGTGGC |  |  |  |
| SICAAS3042 | F-AGATATGCCCCTCGACATCCCTGCT | 60 | 135 | Chr.3 (45,033.6) |
|  | R-GCATACCATGCCTCGCTAAGGCACA |  |  |  |
| SICAAS3081 | F-TGTACCTATGACGAAGCTCACCT | 60 | 227 | Chr.3(46,208.8) |
|  | R-ATCGTCTGTGTCACCATACACTG |  |  |  |
| SICAAS3043 | F-AACGTCGGTCTTGTTGACGACGC | 59 | 214 | Chr.3 (47,022.2) |
|  | R-ACGCGCTCCGGAGTACGAAGTAGGA |  |  |  |
| SICAAS3045 | F-TCCTGCAATGAAGTCAGGGGAGGGC | 59 | 194 | Chr.3 (47,885.4) |
|  | R-TGGGCAACCCTGATGGCTCACAT |  |  |  |
| SICAAS3046 | F-CGACGTCGGGCACGAAAGAAAAGC | 59 | 196 | Chr.3 (47,998.4) |
|  | R-AGCAAGAAGCCAAGAACAGCAGGACA |  |  |  |
| SICAAS3047 | F-GCCGTGGCGGCAAATCTGGAAT | 59 | 181 | Chr.3 (48,474.4) |
|  | R-AAGGTATGCGATTCGGGGGAGGAGC |  |  |  |
| SICAAS3075 | F-CCACGCCTCATTATCCTCAC | 55 | 247 | Chr.3(48,932.5) |
|  | R-GCTGGGCATCTTTTATTTGC |  |  |  |
| SICAAS3064 | F-ACATGTATGGACAGTTGGACACCCCAC | 60 | 250 | Chr.3 (49,416.7) |
|  | R-TCGCGGTCCTTGTGTGATCAGGTC |  |  |  |
| SICAAS3048 | F-TGCAAACTCTTGTACAGGCGCAGACT | 59 | 273 | Chr.3 (49,612.8) |
|  | R-CAACAGAAGAGCAGGGGGCATCCGA |  |  |  |
| SICAAS3076 | F-GGCAGGAGGAGTGAGTGAAG | 55 | 236 | Chr.3(50,101.0) |
|  | R-TCCCATCTTTGTCCCCATT |  |  |  |
| SICAAS3049 | F-GTGAACGCTGGGACATTCTCTTTCGAC | 60 | 122 | Chr.3 (50,166.7) |
|  | R-CCTGCTGAGAGCGATGGAGCAACA |  |  |  |
| SICAAS3065 | F-GCATGCAAGCTGCAAGGATTGTTCGC | 60 | 215 | Chr.3 (50,192.3) |
|  | R-GAGGACCCACTGCATATGCTGCCT |  |  |  |
| SICAAS3066 | F-AGCAGAATCTGATGCTCCCAGTGGAC | 60 | 131 | Chr.3 (50,192.5) |
|  | R-CCGCGGATCCAATCCAAAGAGAAAGGA |  |  |  |
| SICAAS3050 | F-GGCTGACTGCAGATAGGTACAGGGGT | 59 | 198 | Chr.3 (50,350.2) |
|  | R-GCAAAGCTTACCTTGCTGTGGTGGT |  |  |  |
| SICAAS3051 | F-ACCACCACGTACGTTCGTTCGTTTCA | 59 | 177 | Chr.3 (50,461.3) |
|  | R-ACACACACACACACACACACACACAC |  |  |  |
| SICAAS3052 | F-TGGAGGACTTGGACACCTCTCTCTCTC | 60 | 247 | Chr.3 (50,461.4) |
|  | R-GCAAACCTGCTTTCAGCTATCGACCCA |  |  |  |
| SICAAS3053 | F-ACAGCGGTTGCCGTGGCAAGTA | 59 | 147 | Chr.3 (50,591.7) |
|  | R-TGTGTGTGTGTGTGTGCGCGTG |  |  |  |
|  |  |  |  |  |
| SICAAS4015 | F-AGGAATGGGCAGGATGAGGGTGGGA | 62 | 275 | Chr.4 (273.6) |
|  | R-ACTCTCAACTGAACTCCCTGCGGCGT |  |  |  |
| SICAAS4020 | F-GCCGTGCAGCTCAATGCATTTCCCCCA | 60 | 270 | Chr.4 (690.7) |
|  | R-AGGCAAAGTCGCAGCAGCAGCAGT |  |  |  |
| SICAAS4021 | F-TGGAAGGGAAACGGCGAACGGGAGT | 62 | 205 | Chr.4 (694.3) |
|  | R-TCCGGATGCCCTCGCTGCATGGAAA |  |  |  |
| SICAAS4022 | F-TCCGGAGCTCATGCGCACACAGTCA | 60 | 137 | Chr.4 (694.5) |
|  | R-TGAAAGCGTCCGTGCCGTGCATCT |  |  |  |
| SICAAS4011 | F-TCGCTGCAACCAAAAGCTACCTGCGCC | 63 | 187 | Chr.4 (865.0) |
|  | R-TGGGGCGGAGCCACTGGGAGTCAATAA |  |  |  |
| SICAAS4016 | F-ACACCCCGCCGAAACCCTTGTCTCT | 62 | 164 | Chr.4 (1,221.5) |
|  | R-CGCATCAACCTCGCCACCAGCAACA |  |  |  |
| SICAAS4017 | F-TGGGGGCGATTCAGAGCTAGGCCAT | 62 | 267 | Chr.4 (1,300.7) |
|  | R-TCCAGCCATGCACCACAGAGGGACCAT |  |  |  |
| SICAAS4023 | F-GGCGCCATGGTGATACTCTCTCCCCTT | 62 | 161 | Chr.4 (1,353.8) |
|  | R-CGCACTTGATGTCGCCCAGGTTCGT |  |  |  |
| SICAAS4024 | F-TATGGGCCTCGCTTATGGGCCCAACCA | 62 | 151 | Chr.4 (1,918.4) |
|  | R-ATACTGGGCCTCCGGCAGCATCGAA |  |  |  |
| SICAAS4025 | F-TACAGGCCGGTCACCAGCACAGCAGAT | 63 | 185 | Chr.4 (2,087.3) |
|  | R-CGGAACTGCCTGACCGGAAAAGTCGCA |  |  |  |
| SICAAS4052 | F-ACAGCCCTGAAGCTGAAGCCCTGAAGA | 62 | 215 | Chr.4 (2,401.7) |
|  | R-GGCCAACACACGCAAACGTGGCTGGAT |  |  |  |
| SICAAS4026 | F-TGTTAACCCTTCCCGGACGAGCGGT | 61 | 168 | Chr4 (3,769.3) |
|  | R-TGCCAGCGTGTAGCGTACAGTGTGGTT |  |  |  |
| SICAAS4053 | F-TAGGGCGCTGCAGGCAAGAGGCTAA | 62 | 119 | Chr.4 (4,707.9) |
|  | R-TCCAGGCCCAAAGAAGACCTGCTGC |  |  |  |
| SICAAS4028 | F-AACCCTAGATCGCGGCGGGGTTTTG | 62 | 237 | Chr.4 (6,025.1) |
|  | R-GGTCGCCCGAACAAGAGGAAGGAACCA |  |  |  |
| SICAAS4001 | F-TGGGACACCCTTGAAGACGACGAAGGA | 60 | 122 | Chr.4 (6,237.9) |
|  | R-TCGCAGCGCACCGCTAGAATCCAAA |  |  |  |
| SICAAS4018 | F-ACCCTGTCCCTCCCAGGTGGCCTTATT | 62 | 144 | Chr.4 (7,159.6) |
|  | R-TGGGAACCCTGCTGTGGGCTTGGAT |  |  |  |
| SICAAS4064 | F-TACAACCCCCGAACTAACCA | 55 | 169 | Chr.4(7,507.2) |
|  | R-AATGGCACGCTTAGTCCAAT |  |  |  |
| SICAAS4065 | F-TGGCTCCTATTTTCTCCTCAAC | 56 | 170 | Chr.4(7,759.8) |
|  | R-TGTCTTCACTGTTCCAAATGCT |  |  |  |
| SICAAS4029 | F-TCGCTCGGCTCGTTAACCTCCCTACCT | 62 | 208 | Chr.4 (8,774.4) |
|  | R-ACAGATGCATCCCCCGGAACCCACT |  |  |  |
| SICAAS4019 | F-ACTTCCGGGTTGCCGGTGATGTTCCT | 62 | 268 | Chr.4 (9,999.1) |
|  | R-TCCACTTTCCCCATCCTTCTCCGCCGT |  |  |  |
| SICAAS4058 | F-TGCATCGCTTTCTACCTCCT | 55 | 134 | Chr.4(12,832.3) |
|  | R-TTGACATTAAGCGGGGAAAC |  |  |  |
| SICAAS4031 | F-CCCTTTCCATTTTTCCGTGGCCCCCT | 62 | 191 | Chr.4 (12,947.1) |
|  | R-AGATGGGCCTGCATCCCGACAGCAA |  |  |  |
| SICAAS4054 | F-GCGGCTGACGACATCGCACCAAAAGGA | 63 | 196 | Chr.4 (15,511.5) |
|  | R-TCTTGTGCCCCCGACCAGACCTTCA |  |  |  |
| SICAAS4032 | F-TGCTGGGCTGGCAATGCACCAGTGA | 60 | 276 | Chr.4 (17,353.9) |
|  | R-AGGGCCAAGCAAGCCATCCTTGAAGT |  |  |  |
| SICAAS4060 | F-CTGCTGAACCCACTTTTGTG | 57 | 238 | Chr.4(17,354.1) |
|  | R-TAGTGCTGGATAGGGCCAAG |  |  |  |
| SICAAS4033 | F-TCAGCTCACCCCTCTCAACCCGACT | 62 | 162 | Chr.4 (23,843.3) |
|  | R-AGGGGCTAGCTTCAATGGTGGGGGA |  |  |  |
| SICAAS4055 | F-TGACGAGATCTGACGGGATCCGTTGGC | 63 | 204 | Chr.4 (28,574.8) |
|  | R-TGGTCGTCGGATAAAGGTCGGCGGT |  |  |  |
| SICAAS4034 | F-CGGTGGGGTACATCGATCCTGCGACAT | 63 | 209 | Chr.4 (30,398.6) |
|  | R-TTTTCCCTTGAATGGCACGCTGCCGGG |  |  |  |
| SICAAS4035 | F-CCCAAATCAGTTACCATGCCGGCCGAT | 61 | 266 | Chr.4 (31,122.7) |
|  | R-AGTCGCACGGCGCAGATGCTCTCAT |  |  |  |
| SICAAS4036 | F-TGGTGGCGCTGAGACTCAAAACAGCCT | 61 | 153 | Chr.4 (32,750.6) |
|  | R-TCAGGGAGAGCAGCAGTGCAAGAGCA |  |  |  |
| SICAAS4059 | F-TACCCTTTGGGAACAGATGC | 56 | 235 | Chr.4(33,459.0) |
|  | R-AGGTTGGGAATAACCTTTTTGG |  |  |  |
| SICAAS4037 | F-TGATGGGCTTGATTGGGACGGCTCG | 60 | 176 | Chr.4 (33,729.8) |
|  | R-TCCCTTTTGTCGCACGCACGCTGA |  |  |  |
| SICAAS4038 | F-AACGAGACGCATGCTTGCCCGTGAC | 62 | 215 | Chr.4 (33,887.6) |
|  | R-TTCGGCGTCTGGAAACCTGCCCTGA |  |  |  |
| SICAAS4002 | F-TGCCCGGTGGCGCTGGCTTTTTATGT | 62 | 200 | Chr.4 (34,159.6) |
|  | R-ACATCCACCCCCGACTGGACTGAGCTA |  |  |  |
| SICAAS4003 | F-CCCGTCCCCATTTCCTTTTGCGCTGGT | 62 | 181 | Chr.4 (34,506.9) |
|  | R-ACCCCGTGCGTGACTGTGCGTGTAT |  |  |  |
| SICAAS4039 | F-TGTGGCAGCGTAGGTCAGCAACAGACA | 61 | 146 | Chr.4 (34,981.5) |
|  | R-CCGACACCGTGACCATCACGAAAACCA |  |  |  |
| SICAAS4004 | F-AAGAGGTCTGCCTTTGCTTGGCCGT | 60 | 183 | Chr.4 (35,653.2) |
|  | R-TGTGTTTGCACAGCAGCCGACGTGT |  |  |  |
| SICAAS4040 | F-GCAGCACCGTCAAGGTTCCTATCGCAT | 60 | 303 | Chr.4 (35,663.5) |
|  | R-TGGCAGCACAGTAGCGTCCTTTTGCTT |  |  |  |
| SICAAS4005 | F-TACCAAGCCGGGACACCGGACACAT | 62 | 199 | Chr.4 (36,114.8) |
|  | R-ACCCGAGGATTAGCAAGGACAGGCCGA |  |  |  |
| SICAAS4057 | F-TCCATAGGGGTGGTATCTGC | 59 | 108 | Chr.4(36,144.8) |
|  | R-GCAGGAGGATATGGTGGCTA |  |  |  |
| SICAAS4061 | F-CTGTTATGTGATGCTGCCAAT | 56 | 211 | Chr.4(36,164.2) |
|  | R-AATGACTCCAAAATGCACAGC |  |  |  |
| SICAAS4041 | F-ACACGCACCGCACTTCACTTGCACT | 60 | 201 | Chr.4 (36,654.9) |
|  | R-TACCCGATTCGTGCTGGGGGAGCAGAT |  |  |  |
| SICAAS4062 | F-AAGCGAGACCGGAGGAGTA | 57 | 139 | Chr.4(36,930.4) |
|  | R-GGGATGGATGGACATTTCAC |  |  |  |
| SICAAS4013 | F-CCTTGCTTACCTGCACGGCTGCACA | 60 | 221 | Chr.4 (37,684.2) |
|  | R-TGCGAACGCTGTTGGGTTGCTCTCT |  |  |  |
| SICAAS4042 | F-ACAGACGCTGACACGCATGCACGA | 60 | 297 | Chr.4 (37,688.9) |
|  | R-TGCAAGTACCAGCCAGGACGGACAGCA |  |  |  |
| SICAAS4027 | F-ACAACGCCACAGCCAAAGCCTCCCTCT | 63 | 253 | Chr.4 (38,133.1) |
|  | R-TGTGCGAGCCAGGCCAAACACAAGGCT |  |  |  |
| SICAAS4063 | F-GAGAGACTCGTCCTGCAAGC | 59 | 210 | Chr.4(38,136.8) |
|  | R-GGAGTGGGCTTCTGCAATAC |  |  |  |
| SICAAS4043 | F-TCACAGCCGCCAGTTGCGACCGAAT | 62 | 181 | Chr.4 (38,258.5) |
|  | R-TCAGCCAGGCCTTCGTTGATGGCAC |  |  |  |
| SICAAS4044 | F-AGCAGCCGTGCAAATACTGCCTCCA | 60 | 291 | Chr.4 (38,351.1) |
|  | R-ATCCGTGGCGGTCACCGACGATTCT |  |  |  |
| SICAAS4045 | F-GCATCGAGCTCACGTATGGCACGCT | 62 | 109 | Chr.4 (38,739.4) |
|  | R-TCCCGCCGTCGAGGTGAGCAAATGA |  |  |  |
| SICAAS4014 | F-AGCAAGACAGCCAACCAACCGGCCA | 62 | 285 | Chr.4 (38,758.8) |
|  | R-GCCTCACCTGAAACACCGCGAAGGT |  |  |  |
| SICAAS4056 | F-TGTAACGCTCGTTTTGGATG | 55 | 277 | Chr.4(38,784.7) |
|  | R-CAGATCCCACTCCCCATTAG |  |  |  |
| SICAAS4007 | F-AGCGACGACCCCCAAACTCCAAAGGGA | 62 | 161 | Chr.4 (39,343.0) |
|  | R-ACACACACACCCAAGGGAGGAGGAGA |  |  |  |
| SICAAS4008 | F-AGCGAGCTGAGGCAAAGGCAAAGCA | 60 | 173 | Chr.4 (39,349.1) |
|  | R-TTGCGTGCTCCCTTCTCTGTCCACCCA |  |  |  |
| SICAAS4046 | F-TTGCACAAGGGCCCGATGGCTGCTA | 62 | 238 | Chr.4 (39,394.9) |
|  | R-TGGATGGCCAACCGAATCGCTGTGCT |  |  |  |
| SICAAS4047 | F-ACATGTGCCTGCTCCCCATCCCCAT | 63 | 138 | Chr.4 (39,410.7) |
|  | R-GCGAGCTCACCACACTCACCATCTCGT |  |  |  |
| SICAAS4048 | F-ACTGCGAGCTCACCACACTCACCATCT | 62 | 199 | Chr.4 (39,410.8) |
|  | R-TGGAACAGGACCTGGCCGGATGACA |  |  |  |
| SICAAS4009 | F-TGCCATTGCCTGCTGGGTTCATGTCA | 60 | 168 | Chr.4 (39,461.0) |
|  | R-TTCCTCAGCTAACACCTACGGGCGGCT |  |  |  |
| SICAAS4049 | F-CGGCGAAACCAGTCTCTGTCTCGACCT | 63 | 225 | Chr.4 (39,462.4) |
|  | R-GGGGAAGCGAGACCTGGTACTGCAACT |  |  |  |
| SICAAS4050 | F-TCAGAGGTGCGGAAGGGTGGAATGTCA | 62 | 305 | Chr.4 (39,965.9) |
|  | R-ATAGCAAGGGATCCAGCCCGAGCCT |  |  |  |
| SICAAS4051 | F-AGAAAGAGCGTGGAGCCCCGGAAACT | 62 | 217 | Chr.4 (39,983.3) |
|  | R-ATGCACACGTGGTCCTTCCCAGCGGTA |  |  |  |
| SICAAS4010 | F-ACTCCTCCAGCCAACCAACCAACCAAG | 61 | 153 | Chr.4 (40,053.8) |
|  | R-TCCCTCCCCCTGTTCTTCCTTTCAGCG |  |  |  |
|  |  |  |  |  |
| SICAAS5001 | F-GGACTGGGCCCCAAAAGGCTGAAT | 59 | 152 | Chr.5 (185.5) |
|  | R-TCGTCACGTGTTGCTGGACTGCT |  |  |  |
| SICAAS5002 | F-AGCTTCCTGACCTCCAAACCCTCG | 59 | 177 | Chr.5 (277.6) |
|  | R-AAGCTGATTCGGCTCGGCCCAT |  |  |  |
| SICAAS5003 | F-ACCCCATCCCCATGTGTGTTTTCCA | 59 | 176 | Chr.5 (296.9) |
|  | R-AGCAGGCCTCAACGGCCCAAAT |  |  |  |
| SICAAS5043 | F-TGGTCCAGTGTCCCAAAATGAGTGCAT | 59 | 229 | Chr.5 (326.1) |
|  | R-ACACAGTGCTCTCTCCCTTGGCCT |  |  |  |
| SICAAS5065 | F-CAAAAAGGGGTTGTCCTTCA | 55 | 227 | Chr.5(342.6) |
|  | R-CAAAAAGGGGTTGTCCTTCA |  |  |  |
| SICAAS5072 | F-TATGGTACACTACTCTGCCCCTCT | 60 | 232 | Chr.5(1,917.5) |
|  | R-TATGGTACACTACTCTGCCCCTCT |  |  |  |
| SICAAS5044 | F-CCTCCCCCACTCCTCTGTTTTACGCT | 60 | 142 | Chr.5 (2,039.2) |
|  | R-TATTCACGGCGTGTCAGCTTGTGGGA |  |  |  |
| SICAAS5045 | F-GCCAGGTCCAGCTGAACAGACACTAC | 57 | 200 | Chr.5 (2,059.4) |
|  | R-AGCGAAGCACAGCAACAGCAACA |  |  |  |
| SICAAS5082 | F-ATGGTTTGTGCCATTGTTGA | 53 | 154 | Chr.5(3,023.2) |
|  | R-TGCAGCCCAAGAAAAGTAGC |  |  |  |
| SICAAS5004 | F-GTTTGGTTCGCGCAGGGTGACA | 59 | 195 | Chr.5 (3,313.9) |
|  | R-AAGCTGTTGGGTCCGCCTTGCT |  |  |  |
| SICAAS5005 | F-TCTGCCATCAGGCCAAGTCACAGT | 59 | 235 | Chr.5 (3,492.1) |
|  | R-CGGCACCCCCATTTTCTTGAGCCA |  |  |  |
| SICAAS5046 | F-CGAGGAGAATCACCTGGACGTTGGC | 59 | 213 | Chr.5 (4,376.2) |
|  | R-ACTGGCTGATGGTTTGGTGGCGT |  |  |  |
| SICAAS5006 | F-CCCAATTCCGCAACATCCCATTCCCA | 60 | 128 | Chr.5 (4,656.8) |
|  | R-AAGCGGCGAGAGAGCAACCGAACT |  |  |  |
| SICAAS5047 | F-TTGCAGGATTGCTGGCGAGCTG | 59 | 200 | Chr.5 (4,976.2) |
|  | R-CCAGTCCTCGAACGGCTCTTTATCCG |  |  |  |
| SICAAS5048 | F-GGCTATGCTTCGGGATCTAACGGCA | 60 | 200 | Chr.5 (5,972.8) |
|  | R-CCCATTCCTCCTTTCAAGGCATCAGCA |  |  |  |
| SICAAS5007 | F-TCCAACTTTCCTGGGAGCTGCGT | 59 | 185 | Chr.5 (6,287.0) |
|  | R-AGGGCCAGCAAACTGAAGGGACA |  |  |  |
| SICAAS5073 | F-GTTTGTGGTCTCCAACTCAACA | 58 | 160 | Chr.5(6,971.2) |
|  | R-GTTTGTGGTCTCCAACTCAACA |  |  |  |
| SICAAS5008 | F-CCGTCCCAATCAACACACACGCTG | 59 | 178 | Chr.5 (7,959.3) |
|  | R-TTGTGGCCACCACGGTAACGGA |  |  |  |
| SICAAS5085 | F-TTTTGGGTGGGGGATTTTAT | 53 | 165 | Chr.5(8,116.9) |
|  | R-TTTTGGGTGGGGGATTTTAT |  |  |  |
| SICAAS5009 | F-GGATGCTTCCACCAGGCATTGCTGT | 60 | 134 | Chr.5 (8,391.3) |
|  | R-ACCTCACTGGAGCTTTCTTGCACCAC |  |  |  |
| SICAAS5067 | F-TCTGCATAGTCTCTCACCAAGG | 58 | 158 | Chr.5(9,040.2) |
|  | R-ATGTTGCCTTCAGTCCTGTTCT |  |  |  |
| SICAAS5074 | F-ACTACTGTTGCTGGCTTTCG | 57 | 285 | Chr.5(10,269.7) |
|  | R-ACTACTGTTGCTGGCTTTCG |  |  |  |
| SICAAS5010 | F-GCGAAGGCTGCAGGGCTTATGCTA | 59 | 150 | Chr.5 (10,446.1) |
|  | R-AAGCCATACCGGTTAGGCGCGA |  |  |  |
| SICAAS5011 | F-TTGGAGCGTAGCGCATCAGGCA | 59 | 146 | Chr.5 (10,565.0) |
|  | R-AGCCCCTCCGTCTCTCTCACCACAT |  |  |  |
| SICAAS5070 | F-TCCTAAAGCCTGAACTGCAACT | 56 | 198 | Chr.5(12,146.3) |
|  | R-GAAGGAGCAAAAGGAATCAATG |  |  |  |
| SICAAS5012 | F-ATCGAAGGCCGGGAAAAGGATGGC | 59 | 136 | Chr.5 (12,151.4) |
|  | R-TACCACCACGCCCACACGTACA |  |  |  |
| SICAAS5013 | F-GCTGGTGCAGCAGATGCTACGTTC | 60 | 123 | Chr.5 (12,297.9) |
|  | R-GGATTTCCTTTGTCACCCTGCCCGC |  |  |  |
| SICAAS5014 | F-GCTGCATGCTAGCCAAACACAACCG | 60 | 217 | Chr.5 (12,331.7) |
|  | R-TCAGTATCGTGAAGCCCGTTCTCTCG |  |  |  |
| SICAAS5015 | F-CGCAGCAAACACAGCTTATCCCCG | 60 | 197 | Chr.5 (12,561.9) |
|  | R-ACACGGCCGGCCAGGAGATTCATA |  |  |  |
| SICAAS5016 | F-CCCGCAAAAACAATGCACCCCCA | 60 | 256 | Chr.5 (17,505.1) |
|  | R-TCTGTGTGGGAAGGAAGGAGAGGGA |  |  |  |
| SICAAS5066 | F-TCGCTGTCATCTTCGTCATC | 50 | 232 | Chr.5(21,174.4) |
|  | R-CATCTGTCATCACAGCACCAC |  |  |  |
| SICAAS5049 | F-GGTTTCTGCTTCTCCTCCGCTCCA | 60 | 149 | Chr.5 (23,544.2) |
|  | R-AGGACACGGAGACCCTGCTGAACT |  |  |  |
| SICAAS5086 | F-AAGTTGGAGCTGTGCCAAAC | 57 | 232 | Chr.5(25,411.6) |
|  | R-AAGTTGGAGCTGTGCCAAAC |  |  |  |
| SICAAS5071 | F-ACGGCTGCTCTTGCTTCTAC | 55 | 196 | Chr.5(25,933.6) |
|  | R-CTTTGCTTTGCCCTTTCCT |  |  |  |
| SICAAS5018 | F-GCGCCACCAATTGGCTTCGGATG | 59 | 219 | Chr.5 (26,248.7) |
|  | R-ACGAGTGGCCAGAAGACGTGGT |  |  |  |
| SICAAS5054 | F-TTGTGTCCAGGGGGCCATTGCTTC | 60 | 278 | Chr.5 (26,853.8) |
|  | R-CGCCATGGCCTATCACACAAGTCACG |  |  |  |
| SICAAS5087 | F-AAGTTTGGCCGGAACCATA | 55 | 161 | Chr.5(26,897.0) |
|  | R-AAGTTTGGCCGGAACCATA |  |  |  |
| SICAAS5050 | F-GCTGATTTTCCGCGGCTGCTGT | 59 | 245 | Chr.5 (26,916.8) |
|  | R-CACCCAAGGAAGGTGAACTTGGTGC |  |  |  |
| SICAAS5051 | F-TGCAGATGGAGAGTTGGCCTGCAT | 59 | 100 | Chr.5 (26,987.5) |
|  | R-ACCGCAGATGTGTTGAGCAGAGAGT |  |  |  |
| SICAAS5052 | F-TCCCAGAGTTCCAAAGACTGTACGGCA | 59 | 254 | Chr.5 (27,934.9) |
|  | R-TGCGTTTTCCACCCAACCTGTCCA |  |  |  |
| SICAAS5063 | F-CAGAACCATTAAGAAGATGGGAAC | 58 | 182 | Chr.5(27,945.0) |
|  | R-CAGAACCATTAAGAAGATGGGAAC |  |  |  |
| SICAAS5084 | F-ATACCCTGTCCACCGAAACA | 57 | 269 | Chr.5(27,984.4) |
|  | R-CGCTTCTGTGAGTCCTGAGA |  |  |  |
| SICAAS5020 | F-CCAACAAAACAATGCCCTGGGTAAGCA | 59 | 194 | Chr.5 (28,017.3) |
|  | R-CGTGAGCGCCCTTTAGAGGAGGGTA |  |  |  |
| SICAAS5021 | F-TCAATCTGACAGCAGGGGAGCCTCG | 60 | 175 | Chr.5 (28,042.8) |
|  | R-CCTCCCGTACGTGGCATGCAAAATG |  |  |  |
| SICAAS5022 | F-CCAGGAGCAAGCGCGCAAAATCA | 59 | 194 | Chr.5 (29,161.9) |
|  | R-CCCGCTCCAACAAGCCTGTAAGCA |  |  |  |
| SICAAS5023 | F-TCCAACCCGAATCAGGCAGAGGCA | 60 | 167 | Chr.5 (30,351.0) |
|  | R-TTGTGAGCGAGGAAGCAGCAGCAG |  |  |  |
| SICAAS5079 | F-CATCTACATCGCGGAACAACT | 57 | 259 | Chr.5(30,474.9) |
|  | R-CATCTACATCGCGGAACAACT |  |  |  |
| SICAAS5024 | F-TCACCACACGCACGACCAGTCA | 59 | 179 | Chr.5 (30,545.8) |
|  | R-GCTTCTCAGGGGGAAACCAATCGCA |  |  |  |
| SICAAS5025 | F-AACCGCTGTCGCCCATGGCATATC | 59 | 198 | Chr.5 (30,928.9) |
|  | R-TCCGCGTGGGTGCATTTGCTGT |  |  |  |
| SICAAS5053 | F-AACCTGTGGTGTACGGTCTGGGGT | 60 | 228 | Chr.5 (31,068.9) |
|  | R-TGCCCTCCTTGTGAAACCTGGGAAG |  |  |  |
| SICAAS5068 | F-ATGCGTACGTGCTGCCTGT | 59 | 182 | Chr.5(31,398.6) |
|  | R-CGAAGAGAGAGAGAGAGCAAGC |  |  |  |
| SICAAS5026 | F-GTTAGGCACGGTCTTCTTGGCCCT | 59 | 151 | Chr.5 (31,516.1) |
|  | R-TGATCGCGTCGCCCTGGAACAT |  |  |  |
| SICAAS5055 | F-TGTTTTGGGGTCCCTGGACTTGGCT | 59 | 218 | Chr.5 (32,228.9) |
|  | R-TCTGCCTTCTGTCCACATCGCACAT |  |  |  |
| SICAAS5027 | F-TCACGACGACGGTGAGCATTTTGC | 59 | 235 | Chr.5 (32,297.3) |
|  | R-TGATGCGCCGCCTGGTTCTTCT |  |  |  |
| SICAAS5028 | F-GCTCTTCTGTTTGCATCCCCTAACGGT | 60 | 209 | Chr.5 (32,351.6) |
|  | R-TCCCTGTCATTGCCCTGTTCCCCA |  |  |  |
| SICAAS5056 | F-AGAAATCCAACGCCACGCCACG | 59 | 194 | Chr.5 (32,399.4) |
|  | RAGCGAGCGAGATAGAGATAGAGGCAGA |  |  |  |
| SICAAS5029 | F-ACCGGTTGCTACAGCTTCCCCCAT | 60 | 213 | Chr.5 (32,413.2) |
|  | R-ATTTGGGTACTCTCCCCTCCGCCT |  |  |  |
| SICAAS5030 | F-TGCAGAAATGGACAGCCTCCGTATGT | 60 | 221 | Chr.5 (33,265.7) |
|  | R-TTGTCATCTCCCTCGTGCGTGCGT |  |  |  |
| SICAAS5031 | F-AAGGAGCTCGCAGGCAGAAGTGA | 59 | 126 | Chr.5 (33,845.3) |
|  | R-TGCGAGCAGGGTTTGGATCGGA |  |  |  |
| SICAAS5032 | F-AGCAGGGGCAAAAGGAAAGGAGGGA | 60 | 247 | Chr.5 (34,340.6) |
|  | R-CAAGTCGGCAGAGGGTTGCTACACG |  |  |  |
| SICAAS5057 | F-GAAGTAGGAAAGGAACGCAGCGAGCG | 60 | 170 | Chr.5 (34,473.7) |
|  | R-GCACAGGCAGCACCGATTCATTCTCA |  |  |  |
| SICAAS5058 | F-AATGCGAGGAGGAGGAGCGGAAGA | 59 | 180 | Chr.5 (34,706.9) |
|  | R-AAATCGAATGGACGCGGACACCG |  |  |  |
| SICAAS5083 | F-GGCATGCTGCAGTCAGTGTA | 59 | 288 | Chr.5(34,838.9) |
|  | R-GGCCGTGAGTGGTGATGTA |  |  |  |
| SICAAS5059 | F-ACAAGTGTTTGCTCTGCTCTGCTTGTC | 59 | 213 | Chr.5 (35,945.1) |
|  | R-TGCAACGAGCACCTACACGGCT |  |  |  |
| SICAAS5060 | F-AGCAAACAGGAGCCAGCTTGAGGC | 59 | 229 | Chr.5 (36,198.4) |
|  | R-TACATGCTTCGACTTGCACTCATCGGT |  |  |  |
| SICAAS5075 | F-GCGAGGCATTTTCTGTATTCTGT | 58 | 196 | Chr.5(36,293.1) |
|  | R-GCAATGGTTATACATAGGGCAAAG |  |  |  |
| SICAAS5088 | F-AAGATCCAAGTAAGCATCCCATAC | 58 | 174 | Chr.5(36,325.6) |
|  | R-AAGATCCAAGTAAGCATCCCATAC |  |  |  |
| SICAAS5033 | F-TGTCTTCGCAGGACGTGTACCGAG | 60 | 139 | Chr.5 (37,140.0) |
|  | R-TGGCCCATGACAGGGCACGATAAG |  |  |  |
| SICAAS5034 | F-GTGTCCTCGCTCTCCACCAAGACTC | 59 | 150 | Chr.5 (37,301.8) |
|  | R-ACGAGACGTCAAATCCTGCAAATGGC |  |  |  |
| SICAAS5076 | F-AAAGTAAGTCCAGGCCACCA | 57 | 199 | Chr.5(37,612.3) |
|  | R-CCGAAGGCTAAGTTGTCTGC |  |  |  |
| SICAAS5077 | F-CAGGCACACCAGTAAATTGC | 57 | 131 | Chr.5(37,876.0) |
|  | R-CACAATGCAACTAGGATGAACG |  |  |  |
| SICAAS5064 | F-ACCTTGTGCCTCTCGAACC | 57 | 195 | Chr.5(39,761.5) |
|  | R-ACCTTGTGCCTCTCGAACC |  |  |  |
| SICAAS5089 | F-ATCCCCGTAAACGAATCACA | 55 | 203 | Chr.5(39,763.0) |
|  | R-ATCCCCGTAAACGAATCACA |  |  |  |
| SICAAS5061 | F-CCCTTACCGTACCCTATCCGTTGCCA | 59 | 300 | Chr.5 (40,098.7) |
|  | R-GGAAGCAGCCATCGTGCAATTGGT |  |  |  |
| SICAAS5080 | F-AGTGTCCGGGAAGAAAGATGAG | 60 | 133 | Chr.5(40,489.9) |
|  | R-AGTGTCCGGGAAGAAAGATGAG |  |  |  |
| SICAAS5035 | F-ACATCATGCCGACATCGGGCTACG | 60 | 299 | Chr.5 (40,923.8) |
|  | R-GCACAGGACAGGACCTCAAGTTGACA |  |  |  |
| SICAAS5036 | F-AAAGGTAGCCAGCTGCCCCGTGAT | 60 | 146 | Chr.5 (43,124.3) |
|  | R-TGGTTCCATGTGGCCAGAAAAGAAAGC |  |  |  |
| SICAAS5069 | F-GTGATCCATCCTCACTTCATTTG | 58 | 249 | Chr.5(43,141.4) |
|  | R-ATACATGGAGGTCGAGGAGATG |  |  |  |
| SICAAS5037 | F-GGAGAAGCGAAAAGCGAGGTGACCA | 60 | 198 | Chr.5 (43,358.7) |
|  | R-GCGATCATCCATGTAGGAGACTGGGAG |  |  |  |
| SICAAS5038 | F-AAGCTTCGTGTCAGGAGTACAGCCTC | 59 | 159 | Chr.5 (43,420.9) |
|  | R-TCCCGAAGTCGCTCAAGGTGCT |  |  |  |
| SICAAS5039 | F-TGCAGCACACGCAGCGGAATCA | 59 | 240 | Chr.5 (43,787.2) |
|  | R-TCGACAGATAGGGGCCATAGGGGCA |  |  |  |
| SICAAS5040 | F-TCATGCACGACAGCGACACGCA | 59 | 148 | Chr.5 (43,804.3) |
|  | R-ATGAGCTGACCAGGGCTTTGCCAC |  |  |  |
| SICAAS5041 | F-CCTCCTCTTCCTCGAGTTCCCAGCA | 59 | 146 | Chr.5 (44,022.8) |
|  | R-TGGGTTGGCCCACACGACATGA |  |  |  |
| SICAAS5062 | F-GGGGAGGGGAGGGGGTAAAGAGTTA | 62 | 149 | Chr.5 (44,390.7) |
|  | R-ACAGCCTGCTAGCAACCCACAGGTC |  |  |  |
| SICAAS5042 | F-CTCCATTCCGGACGGCCTGTCAAT | 59 | 149 | Chr.5 (44,639.2) |
|  | R-AGCCTGCCTGACCTTGTGCTGT |  |  |  |
| SICAAS5078 | F-CAAGTTACTGACTGGTTGGATACG | 60 | 158 | Chr.5(45,543.8) |
|  | R-CTCAACTAAACGACCACTGACCT |  |  |  |
| SICAAS5090 | F-TCAAGCCGACATAAATCAACTG | 56 | 230 | Chr.5(45,762.4) |
|  | R-TCAAGCCGACATAAATCAACTG |  |  |  |
| SICAAS5081 | F-ACCACAAATGCTGGCCTTAC | 57 | 165 | Chr.5(46,767.8) |
|  | R-GGTTGTACCGATCCGAGAGA |  |  |  |
|  |  |  |  |  |
| SICAAS6069 | F-CGCATTGAAAATCCTCCTTC | 51 | 261 | Chr.6 (7.8) |
|  | R-CCCAACCTAGCCAACCAGT |  |  |  |
| SICAAS6091 | F-CATAAAAATCTCAGCCCCAAAC | 53 | 128 | Chr.6 (54.0) |
|  | R-GTAGCATCTCATGGCTTTAGCA |  |  |  |
| SICAAS6019 | F-GATGGGGATTTCCCTGGTTA | 53 | 256 | Chr.6 (136.8) |
|  | R-GCATGAAGTTTCTCGTGGTG |  |  |  |
| SICAAS6103 | F-TTGATCGACATCCTGTATACCCTA | 58 | 153 | Chr.6(222.9) |
|  | R-ATATTCCTTCTTCCTCCTCCTCTG |  |  |  |
| SICAAS6120 | F-CATGGATAGGAAATGGTCAGC | 57 | 254 | Chr.6(256.0) |
|  | R-GATTCATCCTCCGCGTCA |  |  |  |
| SICAAS6105 | F-AAAGCGTCCAAGAAAGAAGATG | 56 | 188 | Chr.6(515.8) |
|  | R-CAAGAGGCTTAATTTGGTGGTC |  |  |  |
| SICAAS6122 | F-CAGGCACGTTTCTTCTCCTC | 57 | 160 | Chr.6(665.0) |
|  | R-GAATCCTCACTCGCACAACA |  |  |  |
| SICAAS6005 | F-TCACGTTTTTAATGGCTACGC | 53 | 217 | Chr.6 (1,455.7) |
|  | R-ATGCCGATTGTTGATTGGAT |  |  |  |
| SICAAS6123 | F-CGTCCATAACCTTTCCCTCA | 57 | 208 | Chr.6(1,464.4) |
|  | R-AGGGCACCAACCATCAGTTA |  |  |  |
| SICAAS6073 | F-CTTCCCACTACCGAACCAAGA | 53 | 157 | Chr.6 (1,479.6) |
|  | R-GTAGCCGAGGATCTGGAAGAC |  |  |  |
| SICAAS6095 | F-AGCTTCTGACAGGGGAAACA | 51 | 203 | Chr.6 (1,584.1) |
|  | R-GCCAACGGGCTAATTAGGTA |  |  |  |
| SICAAS6058 | F-GCAATATTACCAATGCAGCAAG | 53 | 235 | Chr.6 (1,670.9) |
|  | R-CTGGATTGCACAACAGAGGTTA |  |  |  |
| SICAAS6053 | F-CTCGTGGTCTACGTGGTCAAT | 53 | 199 | Chr.6 (1,844.2) |
|  | R-TTGATTTTCTACTCGCCCTTCT |  |  |  |
| SICAAS6048 | F-GCCATATTATCCTCCTCCTCCT | 51 | 245 | Chr.6 (2,040.8) |
|  | R-ACTTCCTAGTGCCACAAAGCTC |  |  |  |
| SICAAS6072 | F-ACAACAGGTGGATTGTGACGTA | 51 | 225 | Chr.6 (2,447.9) |
|  | R-CCCCAAATCACTAGATGCAAAG |  |  |  |
| SICAAS6079 | F-CAGCAGTCATGGATAGTCTCG | 51 | 110 | Chr.6 (2,476.8) |
|  | R-TACAGCATCCGGTTTCCAAT |  |  |  |
| SICAAS6101 | F-CATGGTGCCTTGCATTTAGA | 55 | 173 | Chr.6(3,493.8) |
|  | R-TGCAGTTCAGTGAGACATACAAAAC |  |  |  |
| SICAAS6114 | F-CCCTACCTTGTCTCCTCTCG | 57 | 141 | Chr.6(4,335.2) |
|  | R-GCCTCAAACGACATGAGGAT |  |  |  |
| SICAAS6096 | F-CATGTACTCGACTGTGGCTGTT | 56 | 297 | Chr.6(4,372.5) |
|  | R-AAAGGCCCTCCTCGATATTTTA |  |  |  |
| SICAAS6102 | F-TCAAAGTTCCTACAGGTGCGTA | 58 | 114 | Chr.6(4,561.8) |
|  | R-TACACTACTGCCGGTTTGTTTC |  |  |  |
| SICAAS6082 | F-ATTGGGCTAGGGCTGCCTAC | 51 | 144 | Chr.6 (5,736.9) |
|  | R-TTGGCTGAACCTTCTCACTACA |  |  |  |
| SICAAS6028 | F-AGGATCAACATGTCCGAAGC | 51 | 103 | Chr.6 (5,816.7) |
|  | R-CAAACCAATAGGGCAAACGA |  |  |  |
| SICAAS6022 | F-CCTTCCCCTTCCTCTCTCTTC | 55 | 176 | Chr.6 (5,868.2) |
|  | R-CCGCTAAACAGACCTCCAGT |  |  |  |
| SICAAS6088 | F-ATAGGAGTTGGCCCATTAAACA | 55 | 174 | Chr.6 (5,951.2) |
|  | R-CGGAAGGGGATAAGGTGAG |  |  |  |
| SICAAS6074 | F-AATGTTCGTTCTCGTTCTCCAT | 51 | 172 | Chr.6 (5,983.7) |
|  | R-GCAACAGATCAGCAATAACCAC |  |  |  |
| SICAAS6006 | F-GGGCGTAGGGCTATGTTTCT | 51 | 147 | Chr.6 (6,221.2) |
|  | R-CCCAGCTAACAATGCTGGAT |  |  |  |
| SICAAS6044 | F-TGGAATAATAACACGTGGCTGT | 51 | 125 | Chr.6 (7,137.8) |
|  | R-TTAGTTGAACGCCTGTCAGTTG |  |  |  |
| SICAAS6065 | F-AGGCCTACTAGTTCAGCTGTTCA | 53 | 272 | Chr.6 (7,585.2) |
|  | R-GATAGGATTGAGGGAAACACCAC |  |  |  |
| SICAAS6060 | F-CCTTCCATTTCCATGCATCT | 53 | 104 | Chr.6 (7,601.6) |
|  | R-CCATTGGAGCCTACGTGTACT |  |  |  |
| SICAAS6055 | F-GGCCCACCCAATATAACAAAC | 55 | 260 | Chr.6 (7,657.6) |
|  | R-ACAAAGTGATGTGCTCCATGA |  |  |  |
| SICAAS6094 | F-GATGTGATTCTACGGTTCGACA | 51 | 249 | Chr.6 (7,682.6) |
|  | R-CTCCCAGCCATTTTCTCTTTTA |  |  |  |
| SICAAS6036 | F-TGTACATGACCTTGCTCGTTG | 50 | 164 | Chr.6 (7,707.4) |
|  | R-TGCGTAACCTTGCTTGTTTCT |  |  |  |
| SICAAS6014 | F-TCTGTCGATATGTGCCGGTAT | 51 | 243 | Chr.6 (7,984.9) |
|  | R-CTGAGGTTTCGTTGGAGAGGT |  |  |  |
| SICAAS6029 | F-GACCATACAGGCACATGCAC | 55 | 197 | Chr.6 (8,388.9) |
|  | R-CGACATGCATACAATACGAGAGT |  |  |  |
| SICAAS6070 | F-GTCTTCCACACCCGAATGAT | 51 | 208 | Chr.6 (9,460.4) |
|  | R-TTCCTAAAACCCCTTTCCAC |  |  |  |
| SICAAS6089 | F-CAGTTCAGCAGCACCAAAAAT | 53 | 164 | Chr.6 (9,701.3) |
|  | R-GTGGAGTATGGCAGGAGCTAAG |  |  |  |
| SICAAS6054 | F-ATTCTTAATCGCAGCACAGACA | 51 | 196 | Chr.6 (9,771.7) |
|  | R-GGCTGCCAAACTAACCCTAATA |  |  |  |
| SICAAS6075 | F-AGTGGTGGTGCTATATCAATGC | 53 | 158 | Chr.6 (9,993.5) |
|  | R-AATGTAGAGTGCCACAGGTTGA |  |  |  |
| SICAAS6084 | F-TTCTCTTCTCCACTGCACCTG | 51 | 165 | Chr.6 (10,231.8) |
|  | R-ACAAAGTTACGTGCGTGTGTC |  |  |  |
| SICAAS6106 | F-TGCTTCCATCATCTCAGGTCTA | 55 | 189 | Chr.6(10,371.2) |
|  | R-GCTGCCGATGTTTTTCAATC |  |  |  |
| SICAAS6083 | F-GCCGCGTTCTAGCTAGTTGT | 53 | 164 | Chr.6 (10,476.7) |
|  | R-CTTCCATCTCCTCGTGCTCT |  |  |  |
| SICAAS6092 | F-GTATCTTGCTTACGGGGTTTTG | 51 | 164 | Chr.6 (12,761.5) |
|  | R-GGGGAGGAAGATAGAGGGGTA |  |  |  |
| SICAAS6097 | F-CCCACAAATCATCAGAAATCCT | 56 | 153 | Chr.6(14,036.8) |
|  | R-TGGAGGCAGATGGTCAATACTA |  |  |  |
| SICAAS6021 | F-GGCAGGATTGGTAAAGCACT | 55 | 249 | Chr.6 (15,114.7) |
|  | R-CCATTGAGAACATGCCAAGA |  |  |  |
| SICAAS6107 | F-TTTCCCCAAAATATGCCACT | 53 | 156 | Chr.6(16,260.4) |
|  | R-AGAAAAGTCCCCAGGCTCA |  |  |  |
| SICAAS6031 | F-TTTGACGTGGAGATGATGACA | 51 | 158 | Chr.6 (16,943.0) |
|  | R-AGCTGCAGCACACTGCTACTA |  |  |  |
| SICAAS6008 | F-AATTGAGCGTAGTGCCACCT | 53 | 280 | Chr.6 (17,955.7) |
|  | R-GTCGACTTAGCGTCCCTCAC |  |  |  |
| SICAAS6023 | F-CCCCTCTCCTTTCTCTCCCTA | 51 | 146 | Chr.6 (18,173.8) |
|  | R-GTAGAGCCAGCTGATGGACTG |  |  |  |
| SICAAS6116 | F-TGATGAATCTCGTGAAAGCAAC | 56 | 158 | Chr.6(18,937.3) |
|  | R-CATTGTCGCTATGACCATCACT |  |  |  |
| SICAAS6024 | F-ACACCAACCAACCAACCAAC | 53 | 174 | Chr.6 (19,220.0) |
|  | R-GGGCCCATGTCAAAAGCTA |  |  |  |
| SICAAS6042 | F-TTCTCTCCATTTTTCCTCCTTG | 51 | 152 | Chr.6 (19,892.2) |
|  | R-TTGACCTAGAGCTACCGCCTAT |  |  |  |
| SICAAS6117 | F-AGAATGTTACAGCAACGGGAGT | 58 | 250 | Chr.6(21,288.6) |
|  | R-GAGCCCGATTGAGTTCTTTTC |  |  |  |
| SICAAS6112 | F-AGGATGGAAGTAACGAATGAGATG | 58 | 244 | Chr.6(21,300.1) |
|  | R-CTTGCCCGAATTGATAGTATTAGG |  |  |  |
| SICAAS6049 | F-GCCCATAGTACCCTCCCTAGAC | 55 | 173 | Chr.6 (21,952.3) |
|  | R-TGTCCTACTGACCTTCCATCCT |  |  |  |
| SICAAS6025 | F-ACAGCGACGAGCTTCTTGAG | 52 | 210 | Chr.6 (22,687.9) |
|  | R-ACCCGAGCCATCCCTAAT |  |  |  |
| SICAAS6071 | F-TATTAAACTCCCGAGACGTGGA | 51 | 223 | Chr.6 (23,692.9) |
|  | R-TGATGAGAACATGCCAACCTTA |  |  |  |
| SICAAS6026 | F-CCAATTCATAGGATCCAAGCAG | 51 | 161 | Chr.6 (23,743.9) |
|  | R-TCAAGTGCGGGTCAGTTATTTA |  |  |  |
| SICAAS6050 | F-GGAATCATTAGCAGGGGAGAC | 51 | 147 | Chr.6 (24,699.8) |
|  | R-TTCTCTCTTTTGGGCTTGGA |  |  |  |
| SICAAS6032 | F-GAAATGGTAGGGAAAGCTCCT | 53 | 115 | Chr.6 (25,322.9) |
|  | R-TCCCTCTCCTCTCTTCCTTTG |  |  |  |
| SICAAS6068 | F-CGTTGGATAGGCTGCCTTAG | 50 | 296 | Chr.6 (25,394.6) |
|  | R-CGGCTGAAACTTGGAAATG |  |  |  |
| SICAAS6003 | F-TTTATGGCAACATGAGATCG | 49 | 124 | Chr.6 (26,914.7) |
|  | R-TGCTTGATGAGGTCTAGGACTG |  |  |  |
| SICAAS6078 | F-CCCACGAAAGAAAGGAAGAAG | 53 | 137 | Chr.6 (27,542.8) |
|  | R-GCCGAGACAGACAGAGAATGA |  |  |  |
| SICAAS6100 | F-CATTAGCTGTTGCCTTTTCTAGC | 58 | 177 | Chr.6(27,722.6) |
|  | R-GGAAAAACTGGATCACTAGGAACA |  |  |  |
| SICAAS6009 | F-GTGAGACCTGACTACACCTTGC | 53 | 240 | Chr.6 (27,752.4) |
|  | R-GTTTTTCCCTGCTTGTCCTAGA |  |  |  |
| SICAAS6011 | F-ATGAGTCTATTTCACCGCCAGT | 51 | 187 | Chr.6 (27,800.6) |
|  | R-ATGGTTCATTGTGGACTCTTGT |  |  |  |
| SICAAS6010 | F-TGCGCAGGTTTGAAAGTGTA | 51 | 119 | Chr.6 (27,855.6) |
|  | R-CTTTCACCTGAATGTGATCGAG |  |  |  |
| SICAAS6061 | F-AGAGAGGGAGCACAAACAAACA | 53 | 174 | Chr.6 (28,124.7) |
|  | R-CAGTGAGGCTTTGGAGATGAC |  |  |  |
| SICAAS6033 | F-TCACCGAGTTTAACAAAGCAAC | 51 | 169 | Chr.6 (28,127.3) |
|  | R-GAGGGAGAGAGAGAAACGATCA |  |  |  |
| SICAAS6013 | F-TGGCTATGCGTCGATAAAGA | 51 | 224 | Chr.6 (28,164.8) |
|  | R-CTGTCCGGGGAACAAGTGTA |  |  |  |
| SICAAS6051 | F-GTGTCGTCACTTCCTTTCGAC | 51 | 116 | Chr.6 (28,307.2) |
|  | R-TTCAGAGATTTCATCGGGTTCT |  |  |  |
| SICAAS6017 | F-GTACTGATCAACCAACGACAGC | 51 | 137 | Chr.6 (28,481.9) |
|  | R-TTCGCTTTTGAGCAGGTATGTA |  |  |  |
| SICAAS6066 | F-CAGCACAGAGACACACACACAG | 51 | 199 | Chr.6 (28,483.9) |
|  | R-CATGCCGACCAAATTTTATAGC |  |  |  |
| SICAAS6027 | F-TGGTTAATGCTTGTCCGTGTTA | 51 | 190 | Chr.6 (28,491.4) |
|  | R-AAGTATCGAGGCAAAAAGATGC |  |  |  |
| SICAAS6059 | F-TTGATTATGGAGGTGCATGGT | 51 | 122 | Chr.6 (28,865.5) |
|  | R-GGCCTCCTTAATTGGTGTTTC |  |  |  |
| SICAAS6012 | F-TCAAGCCTCGTCTCTCAGGT | 55 | 205 | Chr.6 (28,884.2) |
|  | R-GGACACCATTCCTCTTCTGC |  |  |  |
| SICAAS6020 | F-GACAACCGTCTCTCCTCGTC | 55 | 118 | Chr.6 (28,938.5) |
|  | R-GGAGTTTGGAGAGAAACCCATC |  |  |  |
| SICAAS6045 | F-GATCCATTTAGCTCAACAAGCA | 51 | 179 | Chr.6 (28,962.5) |
|  | R-GAGTTTGACCCGAAAATGAAAG |  |  |  |
| SICAAS6076 | F-AGAGGTTGGAGACGGATACTCA | 55 | 187 | Chr.6 (29,148.7) |
|  | R-ACTCTCAACTCTCAAGCACACG |  |  |  |
| SICAAS6046 | F-ATGTTTGTCAGCATCAGCAATC | 51 | 170 | Chr.6 (29,371.1) |
|  | R-CCATGTACCACCACACAAGAAC |  |  |  |
| SICAAS6067 | F-AGTAACAAAGCTCATGGCTCCT | 51 | 189 | Chr.6 (29,485.7) |
|  | R-ACTTCCTTTTTGTTTGGACTCG |  |  |  |
| SICAAS6062 | F-TTGGATGTGGGGGTAAGGT | 53 | 198 | Chr.6 (29,613.9) |
|  | R-GTCCATCCATGTCAGCACAG |  |  |  |
| SICAAS6108 | F-GGCGTGTGCTAAGAAACCAT | 57 | 212 | Chr.6(30,027.6) |
|  | R-CCATCTACCTTTGGCTCGTC |  |  |  |
| SICAAS6109 | F-TACATGGTTCACGCACAGGT | 55 | 161 | Chr.6(30,205.5) |
|  | R-TGCCATGCTGAAGATTGAAG |  |  |  |
| SICAAS6038 | F-GGGCTGAGTTGTATGGATAGGA | 51 | 131 | Chr.6 (30,232.5) |
|  | R-TAACAGAACGGCAAGAGATTTG |  |  |  |
| SICAAS6056 | F-AAGATAACCTTTAGTCCCCGTTG | 53 | 196 | Chr.6 (31,899.8) |
|  | R-CGCTGAGGATGTATATGTGTAGGT |  |  |  |
| SICAAS6081 | F-CGTGACTTAGACTCCACCCTTC | 53 | 291 | Chr.6 (32,335.5) |
|  | R-GCCTGGATTAATAAGCTGGATG |  |  |  |
| SICAAS6080 | F-AACAGCCAATACCACAAGCAT | 56 | 188 | Chr.6 (32,571.8) |
|  | R-GATTGGGTGATGCTACGACTC |  |  |  |
| SICAAS6087 | F-AGCCCTGCAAACAATACTGC | 53 | 210 | Chr.6 (32,625.9) |
|  | R-AAGGAGAGGGACAGGGAGAC |  |  |  |
| SICAAS6002 | F-GACAACAACGATCACGGATG | 53 | 235 | Chr.6 (32,892.1) |
|  | R-TAGGTCCATGCGACTCACAG |  |  |  |
| SICAAS6090 | F-GCCACTACTCGATCCCTCTG | 53 | 266 | Chr.6 (32,899.3) |
|  | R-CGTGCATGTGCTTCACACTA |  |  |  |
| SICAAS6030 | F-GGCTCTCGTTGGATCTGTTC | 56 | 248 | Chr.6 (32,931.1) |
|  | R-CTGCGCCAATATGGTTTCAT |  |  |  |
| SICAAS6047 | F-AACATGAACAAGCAGTGGAAGA | 51 | 246 | Chr.6 (33,041.9) |
|  | R-TTTCGCATAGCATCTGACTGAC |  |  |  |
| SICAAS6018 | F-AAGTATGCTCGTGATCCTTGC | 53 | 224 | Chr.6 (33,067.8) |
|  | R-TACACACCTCCAGCTCCAGA |  |  |  |
| SICAAS6104 | F-CGTAGGGATTTCCTTCCACA | 57 | 224 | Chr.6(33,071.9) |
|  | R-GAGTTATTTGCACGCCCTTC |  |  |  |
| SICAAS6118 | F-GGCGATCTCGTGATGACA | 57 | 281 | Chr.6(33,130.1) |
|  | R-GGTATGTCGAAGGCGTCGT |  |  |  |
| SICAAS6110 | F-GGCTTACACCTTATTAAACACTGGA | 58 | 233 | Chr.6(33,138.3) |
|  | R-CATCTAGTATTCCACACTCCAAAGC |  |  |  |
| SICAAS6111 | F-CACCAGCATCTTGGCTACAG | 59 | 154 | Chr.6(33,357.1) |
|  | R-ACTGCTGCCTTGTCTCCCTA |  |  |  |
| SICAAS6064 | F-GACAGGAGATGAGGTGAGCAG | 51 | 210 | Chr.6 (33,487.9) |
|  | R-CATGCAATGTGGATTCAGACA |  |  |  |
| SICAAS6077 | F-CGGCCATGTGATTCGTATTTA | 51 | 285 | Chr.6 (33,875.9) |
|  | R-TACATTGCTCCTCCTCCCATC |  |  |  |
| SICAAS6004 | F-CCAGAAATAAAGTCCCCCTGT | 53 | 145 | Chr.6 (33,993.8) |
|  | R-CAGTCTCACTCCTCTCGCTGT |  |  |  |
| SICAAS6052 | F-CACCACAGTAGCATCTGTCCTC | 57 | 144 | Chr.6 (34,426.2) |
|  | R-CTCTCTCTCTCTCCCCTCTGCT |  |  |  |
| SICAAS6115 | F-AAAACTCACGTCCGGTCTCA | 57 | 108 | Chr.6(34,850.5) |
|  | R-CCAGGGCACGAGATACAAAT |  |  |  |
| SICAAS6007 | F-TCTACCCTAAGGATTCCCATCA | 53 | 248 | Chr.6 (34,904.9) |
|  | R-CAGTGCACCGTCTCATCTTTAC |  |  |  |
| SICAAS6121 | F-TATACGCCACAGTTCGTCGT | 57 | 248 | Chr.6(35,030.5) |
|  | R-GGTGAGCAACTCGAACACCT |  |  |  |
| SICAAS6113 | F-GGATCTCATCGTAATCGTAAACCT | 58 | 164 | Chr.6(35,037.3) |
|  | R-TGAGTGATTTGTCTCGTAGTAGCA |  |  |  |
| SICAAS6034 | F-ACGATGGATGGGTCTGTGTAAT | 51 | 233 | Chr.6 (35,579.9) |
|  | R-CGCAAGAAATAATAGTGGGAAC |  |  |  |
| SICAAS6098 | F-GCTAATCGCTAATCTCCTCCACTC | 61 | 129 | Chr.6(35,758.0) |
|  | R-GGGGAGTAAGCTGTTGAGAGAC |  |  |  |
| SICAAS6099 | F-ATGCCCCACAGGAGCTT | 57 | 206 | Chr.6(35,813.5) |
|  | R-CAATAAATGCAGCACCAGCAG |  |  |  |
| SICAAS6119 | F-CTTGCCTCCCATCTCAAGAA | 57 | 178 | Chr.6(35,871.3) |
|  | R-CCAATGGGCCTAGATAAACG |  |  |  |
| SICAAS6035 | F-AATACCACACAAGCATCAGGAG | 51 | 165 | Chr.6 (35,880.9) |
|  | R-GGCGATGGAGTGCATTTTATTA |  |  |  |
|  |  |  |  |  |
| SICAAS7090 | F-TAGCGAAATGACGTGAACAAG | 56 | 159 | Chr.7(2,185.8) |
|  | R-ACTTTACCAGCCCAACTCCAT |  |  |  |
| SICAAS7051 | F-GCCCCGGACATAGTTGTTACTG | 58 | 160 | Chr.7 (2,308.9) |
|  | R-TTGTCGCGTTAGCTTAGGATGA |  |  |  |
| SICAAS7001 | F-TCAACCTAACATGGGTAAATGGAAG | 59 | 100 | Chr.7 (3,589.1) |
|  | R-CATGTACACAAAAACCATGGCAGTA |  |  |  |
| SICAAS7077 | F-ACTCTAAGCAAGAGAGCGAGACAT | 58 | 160 | Chr.7(4,377.4) |
|  | R-TGTAGGTTAAGCAGCACGAATTAC |  |  |  |
| SICAAS7002 | F-TAGCACCCCCAAACATACATAC | 56 | 135 | Chr.7 (9,408.8) |
|  | R-TGGTTGAAAGGCTAGATCAACA |  |  |  |
| SICAAS7003 | F-CATCATTGCACGCATGTTC | 55 | 138 | Chr.7 (12,644.5) |
|  | R-TCAACTTTGTCCTAAGAGAGACG |  |  |  |
| SICAAS7057 | F-CTGCCCACAATAATCCAAGAAC | 58 | 177 | Chr.7 (13,017.8) |
|  | R-TTGCAGTTAGTGCCATGAACCT |  |  |  |
| SICAAS7078 | F-GGCCATTTTAAGTCAAGCTGTC | 58 | 190 | Chr.7(13,554.6) |
|  | R-AGTGAGTGGTTCGTGGAGAAAT |  |  |  |
| SICAAS7004 | F-CGCACCACCAAACTAGCAAT | 58 | 199 | Chr.7 (14,093.5) |
|  | R-GAATCACTGGAATGGATCTCTCT |  |  |  |
| SICAAS7005 | F-GCGTGGAGTCCTCTACCTTTTAT | 56 | 180 | Chr.7 (14,650.1) |
|  | R-TCCTCTTTTCTCCTTTTGTGCT |  |  |  |
| SICAAS7007 | F-CTGAAAACTGGACTAGAAGAAGCA | 58 | 230 | Chr.7 (15,818.9) |
|  | R-CTTAATTAGAGCCCACGTCAAAC |  |  |  |
| SICAAS7008 | F-TGGCATCCAGTCATACACTTTG | 58 | 288 | Chr.7 (17,095.8) |
|  | R-GAAGCTGCACTTGGACGCTAT |  |  |  |
| SICAAS7009 | F-GGTCACAGCCAAGTGTTTTGTC | 58 | 270 | Chr.7 (17,637.1) |
|  | R-TCAACTACAGGAATCCCAACCA |  |  |  |
| SICAAS7052 | F-ACAGCCCCATCCTTGATAACAG | 58 | 169 | Chr.7 (17,690.8) |
|  | R-TATCCTTAGTTGGCGTGGCAAT |  |  |  |
| SICAAS7010 | F-TTTTGAAACGGAGGGAGGAGTA | 58 | 203 | Chr.7 (17,839.5) |
|  | R-AGCTCAAATTGGCAGAACCAAG |  |  |  |
| SICAAS7012 | F-GGATGCGAGGAGTGAGGAGAT | 58 | 128 | Chr.7 (18,236.4) |
|  | R-CCATTATTAGCCGGTTCACCA |  |  |  |
| SICAAS7053 | F-CGTCCATTCCGTCTCCTCTACT | 58 | 143 | Chr.7 (18,238.2) |
|  | R-TTATTACTTGGGCGCAGCTTG |  |  |  |
| SICAAS7054 | F-CTGTTTGACGAGGAGCAGGAG | 60 | 107 | Chr.7 (18,243.2) |
|  | R-CCAACGACCATTCCTTACCAG |  |  |  |
| SICAAS7055 | F-GGATGGACATGGATCGTTCG | 60 | 127 | Chr.7 (18,277.6) |
|  | R-ATGTGACGTGACCAGATGACCT |  |  |  |
| SICAAS7013 | F-CCACACGAGGGTCCAAAAGTA | 60 | 255 | Chr.7 (18,292.8) |
|  | R-GGAGATGAGATGGGAGGAGAGA |  |  |  |
| SICAAS7014 | F-GGCTCTCGAGTGTCCTCTCTCT | 58 | 135 | Chr.7 (18,296.2) |
|  | R-AGCAGCAAGAAATGGTGTGTGT |  |  |  |
| SICAAS7015 | F-GTCACCTGGACGAGACAGGATA | 60 | 152 | Chr.7 (18,306.1) |
|  | R-TTTAGCCAGAGCACCAGAACAG |  |  |  |
| SICAAS7016 | F-CTCTGCAACCTTTGCTGCAT | 58 | 190 | Chr.7 (18,434.4) |
|  | R-CATACACTTGGCCACGTTCG |  |  |  |
| SICAAS7017 | F-TGGCCTCCTAAGAGTTGAAGGA | 60 | 252 | Chr.7 (18,436.0) |
|  | R-TAAGGATAGGGCCGTAGGGGTA |  |  |  |
| SICAAS7018 | F-CGTAGCTACTTGTTCCACGAGCA | 60 | 222 | Chr.7 (18,478.9) |
|  | R-CCGCGACTTCATCAGTGAATATC |  |  |  |
| SICAAS7019 | F-ACCACCTCTGCTCTGACCTCTC | 58 | 149 | Chr.7 (18,506.3) |
|  | R-ACGACGAGAAATTCATGCTGTC |  |  |  |
| SICAAS7065 | F-GTAAGGATACGGGCATATCAGC | 60 | 209 | Chr.7(18,536.5) |
|  | R-GAAGGTGGTGTACGAGAAGGAC |  |  |  |
| SICAAS7066 | F-GTAGTCCCGGTAGTGGTAGTGG | 56 | 298 | Chr.7(18,567.7) |
|  | R-ATGCAACAAGATGATCCCTTTC |  |  |  |
| SICAAS7072 | F-GCCGCTCTAGCAACTCCTTA | 57 | 152 | Chr.7(19,706.9) |
|  | R-GATGAATGCAACCCACTCCT |  |  |  |
| SICAAS7021 | F-GGACCTTAGCTTGAGTCCCACA | 58 | 208 | Chr.7 (19,975.7) |
|  | R-GGGTTATGAGTTTCCCCTTTGA |  |  |  |
| SICAAS7023 | F-TCCCCTCACCTGACCTATCTTC | 58 | 176 | Chr.7 (20,184.5) |
|  | R-CGATCATTAGAGGCCAATCACA |  |  |  |
| SICAAS7024 | F-TGGTCGACGTTCTCCACTCTTA | 58 | 242 | Chr.7 (20,228.2) |
|  | R-CCTTGTGCGATTTTATCCCTTC |  |  |  |
| SICAAS7073 | F-AGCTGTTGTTTCATGCTATTCG | 63 | 157 | Chr.7(20,257.4) |
|  | R-GTTGAGAGTGCTGACATTGGAG |  |  |  |
| SICAAS7092 | F-ATAAGGTGGGAGGGAGGGTA | 55 | 279 | Chr.7(20,653.4) |
|  | R-GATTATTAAAGGCGCGGTGA |  |  |  |
| SICAAS7025 | F-GTAACCTGTTTCGGCCTACTGC | 62 | 144 | Chr.7 (20,865.0) |
|  | R-AGCCCTCTCACTGTTCTCTCGT |  |  |  |
| SICAAS7067 | F-CATTGCAGAGCGACACATCT | 57 | 194 | Chr.7(21,357.4) |
|  | R-AGCACGGCGTTCTTGGT |  |  |  |
| SICAAS7026 | F-GAAGTGGCCTAATAAGCATTCG | 58 | 128 | Chr.7 (21,676.5) |
|  | R-ACCCAGATTTCTGGTCTGGTTG |  |  |  |
| SICAAS7056 | F-GTACAGCATAGAGTGCCGCGTA | 60 | 152 | Chr.7 (21,682.5) |
|  | R-AGAGACGAACGAGAATGCGACT |  |  |  |
| SICAAS7027 | F-GGTGAGCCTTCTACGGTCCTAGT | 60 | 229 | Chr.7 (21,687.9) |
|  | R-CGACGGAAGCTCCTTTCCA |  |  |  |
| SICAAS7079 | F-GCCGAACTGGTACAGGAGAGT | 57 | 187 | Chr.7(22,614.9) |
|  | R-CCACGTCCGCCTTCATTA |  |  |  |
| SICAAS7080 | F-TATACCAACCAGGCAACCAAAC | 56 | 148 | Chr.7(22,621.0) |
|  | R-GAATCATCCACTTTCCAAAAGC |  |  |  |
| SICAAS7028 | F-ACAATTGGATGTGGGAGGATG | 58 | 174 | Chr.7 (23,265.4) |
|  | R-CTCTTTGCTACTTTTGGGGACCT |  |  |  |
| SICAAS7081 | F-GGCAAGTAGGGAAACAGCTAAC | 58 | 295 | Chr.7(23,274.9) |
|  | R-CAGAATAGGCACCAAACTGTCA |  |  |  |
| SICAAS7093 | F-CTATGACCGACCGTAGAGCA | 57 | 262 | Chr.7(23,279.6) |
|  | R-TGTGATGATCCGTGGTCTTG |  |  |  |
| SICAAS7029 | F-TGTGGTCGTAGCGGAGTTCTAC | 58 | 160 | Chr.7 (23,315.6) |
|  | R-CCTCCTTTGTGCTTTTCGTTTC |  |  |  |
| SICAAS7030 | F-CCTTATCTCTGTCACCCCATGC | 60 | 307 | Chr.7 (23,387.2) |
|  | R-ATTCCGTGAGAGGTGAAGACTG |  |  |  |
| SICAAS7071 | F-TCGGAGTGGTTCGAGGATAC | 53 | 238 | Chr.7(23,521.5) |
|  | R-TCCAACAACGCAGAAAAAGA |  |  |  |
| SICAAS7031 | F-GCAACATGAACGTGAACCCTAT | 58 | 255 | Chr.7 (23,798.7) |
|  | R-GCTGGGTGGGCTACTGATTAAG |  |  |  |
| SICAAS7059 | F-TATGGTCGCCGATGAGGTAAAT | 58 | 192 | Chr.7 (23,809.2) |
|  | R-AGTCGAACACAAACGCAAGAAG |  |  |  |
| SICAAS7082 | F-CTGCCACGACTACATTTGAGAC | 58 | 281 | Chr.7(24,346.3) |
|  | R-TATGCAAGCAGTCATCACACAG |  |  |  |
| SICAAS7032 | F-ACTTTTGAGTTCCATGCCCTTC | 58 | 175 | Chr.7 (24,380.8) |
|  | R-GCTAAAATCTGAACCCCAACCA |  |  |  |
| SICAAS7060 | F-GCACAGTGCAGAGTTTCTTGGT | 58 | 179 | Chr.7 (24,577.9) |
|  | R-CCCTTGTGCAACTTAGCTTTGA |  |  |  |
| SICAAS7033 | F-CGCTCTCTTCCTCTTCCTCTTC | 60 | 134 | Chr.7 (25,097.4) |
|  | R-AATGTTCCTCTGCTCCCTGTTC |  |  |  |
| SICAAS7020 | F-AAAAGAGAGCCCAATTCGACAG | 58 | 199 | Chr.7 (25,171.2) |
|  | R-CTCGTACGTGACGTGATGGTAG |  |  |  |
| SICAAS7083 | F-TGCCGGTAGAGTTCATATTTCA | 56 | 270 | Chr.7(26,946.4) |
|  | R-CGTCCTTCGCTTTTAACTTTTC |  |  |  |
| SICAAS7034 | F-AAGCCTAGCGATTAGGTTCACG | 58 | 203 | Chr.7 (26,984.7) |
|  | R-CATGTCTTCTTTTCCACCACGA |  |  |  |
| SICAAS7035 | F-AACCACTGCCTTGCTATCCTACA | 60 | 356 | Chr.7 (27,313.9) |
|  | R-CGATCAGTCCTTCCAGCATTAGT |  |  |  |
| SICAAS7084 | F-CTGCTATCCTTGCTGGAGAATC | 56 | 229 | Chr.7(27,367.4) |
|  | R-AAAAATTCCGAGGTATGGGTGT |  |  |  |
| SICAAS7061 | F-TCTTTGTTCTCTCTTGGCCGTA | 58 | 177 | Chr.7 (27,375.9) |
|  | R-CACGCAAACCACAAGACAGTG |  |  |  |
| SICAAS7062 | F-ACCAGTTGACATGCTCGGTTC | 58 | 193 | Chr.7 (28,378.2) |
|  | R-CACGGCAGAGATTCTGAAAACA |  |  |  |
| SICAAS7074 | F-ATCATCATCCCCTGCTTCAC | 57 | 182 | Chr.7(28,669.6) |
|  | R-TGCTTGCTTGCACTCCTGTA |  |  |  |
| SICAAS7038 | F-GCGTTAATGGGCTGGAATTATG | 58 | 344 | Chr.7 (29,176.6) |
|  | R-TCATGTACGTCGCTAATCGTGA |  |  |  |
| SICAAS7091 | F-CTCGGGGAAAGAAGATGATG | 57 | 213 | Chr.7(29,829.5) |
|  | R-GAGAAGATAAAGCGGCCACA |  |  |  |
| SICAAS7039 | F-TCAACGTCTTTTGCCACTGAAG | 58 | 246 | Chr.7 (29,932.0) |
|  | R-CCAGTTAAGACTGTGGGGGAGT |  |  |  |
| SICAAS7040 | F-CCGTCCTCTTGTGTTTCTTCCT | 60 | 202 | Chr.7 (30,575.3) |
|  | R-ATGGCCTCTGAATGTGGGTACT |  |  |  |
| SICAAS7086 | F-TACCAAAGCAGAACATCAGAGAAG | 58 | 102 | Chr.7(30,587.3) |
|  | R-AGTGCCTGTATGAATGAAGTAGCA |  |  |  |
| SICAAS7070 | F-CCTTGGACCTCTTCTTGTGC | 55 | 218 | Chr.7(30,609.8) |
|  | R-TGGTGATTGCATCCACTTGT |  |  |  |
| SICAAS7069 | F-CTTGTAATCGTTCTTGCTCTTGC | 58 | 189 | Chr.7(30,669.4) |
|  | R-GTGCTGTCGGCTATCAACAAC |  |  |  |
| SICAAS7085 | F-CTGACAGACGGGATGAACG | 57 | 134 | Chr.7(30,728.8) |
|  | R-AGGGCACAAGTCAAACAACG |  |  |  |
| SICAAS7075 | F-CCTGTTTCTGTCGCACTTCA | 57 | 190 | Chr.7(31,067.8) |
|  | R-CTGAACCACCGCAGGTCTAT |  |  |  |
| SICAAS7041 | F-TACTGCCGCCTGTGAGAAAGTA | 60 | 214 | Chr.7 (31,215.3) |
|  | R-GAGAACTGGAGGGAGAGTGGAG |  |  |  |
| SICAAS7068 | F-GAAACGAAACCAAGGACAGATAAC | 58 | 159 | Chr.7(31,601.0) |
|  | R-TCTTTGTGCAGAGGGTTAATACAC |  |  |  |
| SICAAS7042 | F-GGTGGGTAGGTGTTGGGTGTAG | 60 | 199 | Chr.7 (31,999.4) |
|  | R-CCTGCTGTTTCTCTCCAGATCA |  |  |  |
| SICAAS7063 | F-AGGGGTCAGCATTTCAATAGGA | 58 | 147 | Chr.7 (32,169.8) |
|  | R-ATGTGTGTAGTGTAGGGGCCTTG |  |  |  |
| SICAAS7076 | F-TCACAAGAGCAGCCACAACT | 57 | 159 | Chr.7(32,705.0) |
|  | R-TGCCTTCCTCGCCATATAAC |  |  |  |
| SICAAS7043 | F-GCTCCTACCAAGCAGCAACTC | 58 | 135 | Chr.7 (32,828.0) |
|  | R-TTGAAGTGTCATCACCCAAAGC |  |  |  |
| SICAAS7044 | F-GGCTTTCAATTCGGTGTCGT | 58 | 202 | Chr.7 (33,005.2) |
|  | R-ATAATGCACGCTCCATCATCAG |  |  |  |
| SICAAS7045 | F-CGTCTCCTGAACCAAGCCTATC | 60 | 197 | Chr.7 (33,221.5) |
|  | R-TCTCCTCCTCATCTTGGGTTTC |  |  |  |
| SICAAS7087 | F-GCATATTGCACTGACAACAATCAG | 58 | 228 | Chr.7(33,285.7) |
|  | R-GGATTCTAAGTCTCTCGCTGCTC |  |  |  |
| SICAAS7046 | F-CTCCAGCTAGCCATCAGTTGAG | 62 | 200 | Chr.7 (33,866.9) |
|  | R-GCACGGTGGAGTCACATACATAC |  |  |  |
| SICAAS7064 | F-GCTATGTGTGTAGCCCACCATTC | 60 | 207 | Chr.7 (33,940.0) |
|  | R-AGCATGTAGATGGTCCTGTTGC |  |  |  |
| SICAAS7048 | F-CAAGATAGCTCCACAACTGGTCA | 60 | 177 | Chr.7 (34,030.2) |
|  | R-CAACTTAGTGCATCAACCACCTC |  |  |  |
| SICAAS7049 | F-GCAGATCTAGCGTCATCGTTTC | 60 | 164 | Chr.7 (34,204.1) |
|  | R-AGTCAAGCCTCCTGTGTGTGTC |  |  |  |
| SICAAS7094 | F-CGATTGCTTGGTGGTCTAGC | 59 | 300 | Chr.7(34,509.6) |
|  | R-GGATCTCTAGCCTGCCTGTG |  |  |  |
| SICAAS7089 | F-TGAGTTATGGAGTCACAGGGAGTA | 58 | 239 | Chr.7(34,509.9) |
|  | R-TGAAGCAGCCCTATATGAGTATGA |  |  |  |
| SICAAS7050 | F-GAATTTCCCACTCCACTCCACT | 60 | 207 | Chr.7 (35,806.5) |
|  | R-GGGGTGCTGTCTTGTTTCTTTC |  |  |  |
|  |  |  |  |  |
| SICAAS8030 | F-GAGCAGAGTGCAATACAACCAC | 56 | 213 | Chr.8(144.1) |
|  | R-AAGCAAGGTCAACTCCATTCAT |  |  |  |
| SICAAS8010 | F-AGCTTGTGCCTGTAGAGAGCCAGCGA | 60 | 199 | Chr.8 (2,244.5) |
|  | R-AGCAACCATGCAGGAGCAGCAGGA |  |  |  |
| SICAAS8011 | F-TCCCTGCTTCCTCTCTCACCTCCTCCA | 62 | 193 | Chr.8 (2,966.1) |
|  | R-TGGGTATCCCTCCTCCACTGCCCTT |  |  |  |
| SICAAS8017 | F-GAAGCGTAGAGCGGACAATC | 59 | 248 | Chr.8(4,206.8) |
|  | R-TCCTTACCCCGTCGAGCAT |  |  |  |
| SICAAS8001 | F-ACCAGCACAAGCCACACGCA | 55 | 134 | Chr.8 (7,443.5) |
|  | R-TGGCAACAACCAAATTAAGGGCCA |  |  |  |
| SICAAS8033 | F-TGCTGTGTTATCGAGGTTGG | 57 | 169 | Chr.8(7,849.8) |
|  | R-CTTTCCCTGCAAGTGGGTTA |  |  |  |
| SICAAS8022 | F-CACTTCCGGGTGTCAGATGT | 55 | 200 | Chr.8(8,247.4) |
|  | R-TCCTCTGGTTAGATGCCAAT |  |  |  |
| SICAAS8002 | F-GCTCGGGCAAAGGCGATGATGT | 57 | 127 | Chr.8 (9,139.7) |
|  | R-ATTGCAGCGCCCGTTAGTCG |  |  |  |
| SICAAS8018 | F-CTACAAAGTCCGCGTGAACA | 55 | 130 | Chr.8(10,531.8) |
|  | R-CTTTTCCGGTTCAATTCCAG |  |  |  |
| SICAAS8003 | F-TTATGCCAATGTCCCCGGCGGT | 59 | 138 | Chr.8 (10,551.5) |
|  | R-CGCCGTTTCCGATCCAAGGCAT |  |  |  |
| SICAAS8005 | F-CCTTTTCTCTTTGGGATTCAGGCCA | 57 | 120 | Chr.8 (13,178.3) |
|  | R-TGAAGCGAACCGACAAGGGAGG |  |  |  |
| SICAAS8029 | F-AAGCAACAACTTCAACCCTGA | 56 | 265 | Chr.8(14,280.6) |
|  | R-CTCCACTGTCTTCAGCCAATC |  |  |  |
| SICAAS8032 | F-CACGTTCATCTAGTCCGAGTCA | 58 | 146 | Chr.8(15,529.3) |
|  | R-CTCTTGTTGCCGATTTACCTGT |  |  |  |
| SICAAS8024 | F-ATAGGCACGATACCTGTTACCAAT | 58 | 205 | Chr.8(19,621.0) |
|  | R-CAGTGGTGGTTTCTATTTCTACCAT |  |  |  |
| SICAAS8006 | F-CGTTGCGCCTGTCACTATGCCT | 57 | 122 | Chr.8 (21,581.9) |
|  | R-TCGTTCATGTCATCTCTTCTATGTCGC |  |  |  |
| SICAAS8031 | F-AGATCGAAGATAAAACCGCATC | 56 | 232 | Chr.8(22,776.4) |
|  | R-AGACACACCATCAACCAAGAAGT |  |  |  |
| SICAAS8007 | F-ACCTGATAGTTGGAAGAAACTACCGCA | 55 | 144 | Chr.8 (23,075.7) |
|  | R-AAGGGTTTGAACAATTCAGTACGTGGT |  |  |  |
| SICAAS8015 | F-AGCCCGACCTAAGCATCTCCCTGCT | 62 | 233 | Chr.8 (23,361.7) |
|  | R-TTGGGGAAGCTCCAAGCTCCAAGCCCA |  |  |  |
| SICAAS8012 | F-TCCCGCACGAACGATCCCGAACAGA | 62 | 157 | Chr.8 (26,283.1) |
|  | R-AGGTGTGCCGTCCAATGTTCCCCGA |  |  |  |
| SICAAS8021 | F-CAGGAGAGCCACAAGAGTGTC | 60 | 188 | Chr.8(26,264.7) |
|  | R-GTCGCTTGTCCTACCACCTAAT |  |  |  |
| SICAAS8013 | F-TCTGGGTGGCTTAGCTTCGGATGAGGA | 62 | 171 | Chr.8 (27,544.0) |
|  | R-AAGCGAACAAGGAAGCGGCGGTGGA |  |  |  |
| SICAAS8025 | F-ACAGCGTTTATTACTTGGAGGTTC | 58 | 228 | Chr.8(28,414.7) |
|  | R-CTGGAGAATGTTCGCTACAAAAG |  |  |  |
| SICAAS8023 | F-ACAGCGAGCAGCAGTGAGAT | 55 | 242 | Chr.8(28,929.1) |
|  | R-CGTCGGTAACATGAAAATGC |  |  |  |
| SICAAS8035 | F-TCCTGCACCAACAACTTGAC | 57 | 177 | Chr.8(29,479.7) |
|  | R-TGATGAAGAAGGTGCTGTCG |  |  |  |
| SICAAS8026 | F-GGAATACTCTTTCGCATGACAATC | 58 | 277 | Chr.8(30,749.4) |
|  | R-GCTGCTTGTAATAAGGACACAGAC |  |  |  |
| SICAAS8016 | F-GGCCAAGAAGCAGAAGCAG | 59 | 227 | Chr.8(31,318.3) |
|  | R-CGTCAAGCACTCCCATAACTTAG |  |  |  |
| SICAAS8027 | F-GCAAGATAATGGACGAAACCTTAC | 58 | 247 | Chr.8(31,915.1) |
|  | R-ACTCCCTAACAAACTCATCAGCAT |  |  |  |
| SICAAS8020 | F-CAGAGACATGTGCGGAAGGT | 58 | 263 | Chr.8(32,458.5) |
|  | R-TATGAGGAAGCTCACGTCGATT |  |  |  |
| SICAAS8008 | F-TGTAACATGCGTGCGTGCGTGCGT | 60 | 143 | Chr.8 (32,849.5) |
|  | R-TTGGTGAAGTCCAGCGAGTAGTCCGGC |  |  |  |
| SICAAS8009 | F-TTGAACGAACAGACGCGCAGGGCA | 60 | 148 | Chr.8 (34,170.3) |
|  | R-TACCTGAAGCTGCTCGATGGGGAAGGG |  |  |  |
| SICAAS8028 | F-GTTGCAGTTTCCATGCAGTTAG | 58 | 178 | Chr.8(34,290.9) |
|  | R-TCGAGAACATCTTGTGAGCATC |  |  |  |
| SICAAS8014 | F-TCCGACTGTGCATGTGCTGTGGTCG | 62 | 201 | Chr.8 (37,645.1) |
|  | R-ACGAACGCCCTGTCGCTCTCTCCAT |  |  |  |
| SICAAS8019 | F-GACCAGGAGGCTGACAGAAC | 59 | 202 | Chr.8(40,386.3) |
|  | R-AGCAATGGGGAGGTACAGC |  |  |  |
| SICAAS8034 | F-GCTCTCTGTCGCAGTAGCTGTA | 58 | 186 | Chr.8(40,390.1) |
|  | R-AACCTAGGACTCCAATTAACAACG |  |  |  |
|  |  |  |  |  |
| SICAAS9001 | F-ACCACTAACCTGCTCGATCATCTTCT | 57 | 171 | Chr.9 (206.6) |
|  | R-ATGGTAGTACTGACGACGACGGAT |  |  |  |
| SICAAS9002 | F-TGTGCTAGCTACCGCAAAAGCCA | 59 | 279 | Chr.9 (208.9) |
|  | R-CTCCAAGCCCCTGCGCTGAAATC |  |  |  |
| SICAAS9003 | F-CTTGTTGCGAGCCATGGCGAAG | 59 | 130 | Chr.9 (298.9) |
|  | R-GGGCGGGTTTTCATGGAGGCAG |  |  |  |
| SICAAS9004 | F-GGGACAGGGACATGGTGGCA | 57 | 122 | Chr.9 (327.2) |
|  | R-GGGGGATGGAAGAGAGGGACCG |  |  |  |
| SICAAS9005 | F-TTGGGTGCACCGGGATTTGCAG | 55 | 108 | Chr.9 (350.1) |
|  | R-CAAGCTCTATCCCCTGGCGTCG |  |  |  |
| SICAAS9069 | F-GCTTCTTCCTCCCGTGGGTCGT | 55 | 136 | Chr.9 (406.7) |
|  | R-GCCGCTTCGGAGGAACTGCATC |  |  |  |
| SICAAS9115 | F-CTCCCCCTTCTAAAGCCCTA | 59 | 164 | Chr.9(477.3) |
|  | R-GAGGGTGGGGTAGGAGAAGA |  |  |  |
| SICAAS9107 | F-CAGGGATGGAATTGGAAATG | 55 | 199 | Chr.9(506.0) |
|  | R-GATGGATTGGATGGATGAGC |  |  |  |
| SICAAS9006 | F-CTGCTGCAAGCAGCGACTCCG | 55 | 144 | Chr.9 (612.6) |
|  | R-CGAGGTTAGAGGCGATCCCCCA |  |  |  |
| SICAAS9007 | F-TGGCACCAATCGCTCTCGCA | 59 | 135 | Chr.9 (684.5) |
|  | R-GCTCGTTCGTTCACGCAGGAAT |  |  |  |
| SICAAS9116 | F-CAATCATGCCACGGTGTG | 57 | 289 | Chr.9(1,215.5) |
|  | R-AGAGAGGGGAGACCAGACAGA |  |  |  |
| SICAAS9008 | F-ACAAGGAAAATGACTTTGCCCAAGACT | 57 | 200 | Chr.9 (1,835.9) |
|  | R-CCTGGGGGCCCACTGTACG |  |  |  |
| SICAAS9009 | F-GGACCAAATACGATTTTTGCCTCATGC | 57 | 198 | Chr.9 (2,022.4) |
|  | R-AGCACAACACTTGTTTAGGCCAACT |  |  |  |
| SICAAS9010 | F-AGCGTGCAAGCTACCAGCAG | 59 | 178 | Chr.9 (2,054.5) |
|  | R-AGAGACAAGGGCACTCGCCA |  |  |  |
| SICAAS9011 | F-TGGCGGTACACTACTCGCTGCT | 57 | 127 | Chr.9 (2,141.9) |
|  | R-ACTGCCGGACGGGGGATATGAC |  |  |  |
| SICAAS9012 | F-GGCTACAGCACACCTGACCTGACT | 55 | 144 | Chr.9 (2,496.3) |
|  | R-CCCGATCGAGCAGCTGAGCAAC |  |  |  |
| SICAAS9097 | F-AATGGACGAGCAACCAAATC | 55 | 124 | Chr.9(2,716.5) |
|  | R-CAGCTCCTGCACATGACAAC |  |  |  |
| SICAAS9013 | F-CCTGAAATTTGTGTTGCTTGGCATGA | 55 | 126 | Chr.9 (2,876.0) |
|  | R-TTCAGCAACGGATTACAGTCTCAAACA |  |  |  |
| SICAAS9095 | F-GGCCCCTGGTAGTAACCTTTTA | 58 | 202 | Chr.9(2,878.5) |
|  | R-AAGATGAACGATCCCAAGTACG |  |  |  |
| SICAAS9014 | F-AAGGTGGTGGCGCGATGACG | 59 | 222 | Chr.9 (2,878.7) |
|  | R-CCGCGGGCATTCCAGATGCT |  |  |  |
| SICAAS9015 | F-CCGACGCTGCAAAGGTCGCT | 59 | 145 | Chr.9 (3,118.9) |
|  | R-GGGCGCTGTGTGATGCGGA |  |  |  |
| SICAAS9016 | F-AGCAACACTTCTGCACCAACAGA | 61 | 150 | Chr.9 (3,305.3) |
|  | R-TTGCCAGTTGCTGTCATGCCT |  |  |  |
| SICAAS9017 | F-TGGGGTAGCAAAGTGAGACAACGC | 59 | 290 | Chr.9 (3,834.6) |
|  | R-CACATGTGAGCAGTCAATTAAGCCCA |  |  |  |
| SICAAS9018 | F-TGCTTTGCATTGGACGACCAGAGA | 57 | 131 | Chr.9 (3,852.3) |
|  | R-GCCGGAGGCTTCTTCCGACAG |  |  |  |
| SICAAS9019 | F-GGCCCAGACTAAACAAACAAACACACA | 59 | 105 | Chr.9 (3,899.9) |
|  | R-GCTGGCTCTACTGCGGATGGC |  |  |  |
| SICAAS9020 | F-CCCACAGACATGCGGAAAGGCA | 57 | 140 | Chr.9 (4,400.6) |
|  | R-AGGGGACGACGGACTGGGAAAC |  |  |  |
| SICAAS9071 | F-GGAGAGGCAGCATCCGGAAAGA | 59 | 196 | Chr.9 (4,446.9) |
|  | R-GCCCAGCTATGCTCGAAGCCAG |  |  |  |
| SICAAS9072 | F-TCTGCAACCAGCTGACCAATGGC | 59 | 133 | Chr.9 (4,726.6) |
|  | R-GTAAGATGGGTGCGGGGTGAGGT |  |  |  |
| SICAAS9022 | F-AGCTGCGGTACCCATCTTGCG | 55 | 160 | Chr.9 (4,895.7) |
|  | R-GCGCCCTCAACATCTCCGCTC |  |  |  |
| SICAAS9023 | F-ACAGATGGCAATCCAAAGAGTCGTTTC | 59 | 172 | Chr.9 (4,931.3) |
|  | R-CCATGGCAGCTCTTTTGCCAGGT |  |  |  |
| SICAAS9024 | F-CTCGGGTCGTGTGGACACTGTA | 57 | 288 | Chr.9 (5,231.7) |
|  | R-TGTTGTTTAAGAGGCTTAAGGAGGTCG |  |  |  |
| SICAAS9025 | F-ACGCGTGCGTTACGAAAAAGC | 59 | 234 | Chr.9 (5,378.4) |
|  | R-ACCGGCGTCAAGGACCAAGGA |  |  |  |
| SICAAS9026 | F-TTGGGGAAATGAACTGTCAGAAAAGGT | 57 | 165 | Chr.9 (5,705.9) |
|  | R-ACCACAATCATGTCCCCTAACAAGGT |  |  |  |
| SICAAS9074 | F-GCTTTGCCCCCGCCAAATAGC | 57 | 246 | Chr.9 (6,020.9) |
|  | R-CGCGCTAGCTGTAGCAGCCTTC |  |  |  |
| SICAAS9027 | F-AACTCACGGGGAGCTCGGAGAC | 59 | 111 | Chr.9 (6,228.7) |
|  | R-TCCTGCGCTCTCTTCCTCGACA |  |  |  |
| SICAAS9075 | F-TGCTACGCCACAAGCCCACAAAC | 61 | 199 | Chr.9 (6,290.9) |
|  | R-CACACGCACCATGGATCCCACAT |  |  |  |
| SICAAS9028 | F-ATGCATCACCAGCTCCAGGCAC | 61 | 189 | Chr.9 (6,345.4) |
|  | R-GCATGCGCGGATCATGCATTTG |  |  |  |
| SICAAS9030 | F-GCAAGCAACAGTCCATGGCCGA | 59 | 161 | Chr.9 (6,681.4) |
|  | R-GCTGCGAGCTTGCGTGACAGA |  |  |  |
| SICAAS9031 | F-GGCCCTCCTCTCTTCCTCGCAT | 57 | 108 | Chr.9 (7,330.4) |
|  | R-CGCGGTTGGCAACACCCTGAC |  |  |  |
| SICAAS9032 | F-GCAACCTCGGCATGAAGCTCGT | 60 | 135 | Chr.9 (7,501.1) |
|  | R-GCAGTTCACTCCAGGGCAAGCA |  |  |  |
| SICAAS9033 | F-GTGAACCTGCGAATCTCGGGGC | 55 | 156 | Chr.9 (7,824.4) |
|  | R-ACGAACGAACGCGAACACACAC |  |  |  |
| SICAAS9086 | F-AAGGAGATCCCCACTTGGAC | 59 | 153 | Chr.9(8,098.8) |
|  | R-CTGCTCTTGTTCAGCGTGAG |  |  |  |
| SICAAS9099 | F-ACAATTCGGCAAAAAGCAAG | 53 | 200 | Chr.9(8,140.3) |
|  | R-GGACGTGGCTTCTGACTGAT |  |  |  |
| SICAAS9029 | F-CCCCCTCTGGTTACTGCGCTC | 61 | 102 | Chr.9 (8,204.7) |
|  | R-GCATGCGCGGATCATGCATTTG |  |  |  |
| SICAAS9130 | F-CACCAGACATCCCACTATCG | 55 | 298 | Chr.9(8,322.9) |
|  | R-TTAGGCTATTTCTGCCAACG |  |  |  |
| SICAAS9034 | F-TGGATTGATGTGGGCAAACAAGGT | 59 | 200 | Chr.9 (8,772.6) |
|  | R-CGCATCCATACCACTTTGGGACTG |  |  |  |
| SICAAS9132 | F-ACCAATACGACAGCCTCTGC | 59 | 270 | Chr.9(8,861.0) |
|  | R-GGAGGCAGAAAGATCAGTCG |  |  |  |
| SICAAS9036 | F-CGCCGCTCATCCTCTTCCACAC | 59 | 114 | Chr.9 (9,696.0) |
|  | R-GTGCCCATGAACGGATCGCACT |  |  |  |
| SICAAS9037 | F-CAGCTCTGTGCCCACACTACCA | 59 | 136 | Chr.9 (10,217.3) |
|  | R-TCACATCTCAGCGCCAGGAGGC |  |  |  |
| SICAAS9041 | F-AAAACATCAGCCCAGCCATGTGA | 55 | 242 | Chr.9 (10,667.5) |
|  | R-CCCAGCTTAATTTGTCGCCATCGGT |  |  |  |
| SICAAS9042 | F-ATGCCAGGCGTGCCTTCAGC | 59 | 265 | Chr.9 (10,772.8) |
|  | R-GGGTCGTCAGAGAGCGTGCAT |  |  |  |
| SICAAS9043 | F-CCTACCGGATTGGCCTCATCAGC | 57 | 176 | Chr.9 (10,773.8) |
|  | R-TGGCACGCACATCTTTGCCAC |  |  |  |
| SICAAS9044 | F-CGAGCAATTCTCTAAACGTGCAGC | 57 | 200 | Chr.9 (11,000.6) |
|  | R-CGACATGCTCCAGACCGCAGA |  |  |  |
| SICAAS9045 | F-ACAGCAATAATGGAGGAATGACGGC | 57 | 180 | Chr.9 (11,000.7) |
|  | R-AGCTCCATGGGGCACCATAACGA |  |  |  |
| SICAAS9046 | F-AGGGAGGTTGGCCGATGATGGT | 59 | 116 | Chr.9 (11,197.4) |
|  | R-CGCTACACCCCTTCAAAACGGCA |  |  |  |
| SICAAS9108 | F-GACATAGGGAGGGAGATTATTTTG | 58 | 164 | Chr.9(11,244.1) |
|  | R-GCAGGGAGAATATCAGTCAGTCTT |  |  |  |
| SICAAS9076 | F-TGAACCGTGATGGAATGATACGGTGA | 57 | 192 | Chr.9 (11,253.2) |
|  | R-CGCTGCTACAGTACCCGACGC |  |  |  |
| SICAAS9100 | F-TACGGGTGCTCCTTCAATTC | 57 | 206 | Chr.9(11,390.9) |
|  | R-TTGGGTACAAGGTTGGTGCT |  |  |  |
| SICAAS9087 | F-CAAGAATGCCAGATTATCCACTG | 58 | 150 | Chr.9(12,497.9) |
|  | R-GAGAGGTGGTGATGGGAGTAAG |  |  |  |
| SICAAS9117 | F-AGTTGGCTGCTGGATTTATACTG | 56 | 285 | Chr.9(13,144.3) |
|  | R-TGTGATCCCCAATTAAAAGGAC |  |  |  |
| SICAAS9049 | F-GAGGAAGACGTCCAATTTCTAGGGC | 59 | 189 | Chr.9 (13,669.5) |
|  | R-GCGCCACACTGGTTGGGTCA |  |  |  |
| SICAAS9050 | F-GGGCTTCGCCCTCCCTCTTCT | 61 | 120 | Chr.9 (14,021.6) |
|  | R-CTCCCGGCCGTTGTTGCTGAC |  |  |  |
| SICAAS9079 | F-AAGTGCGCGAAGCCCAGTCG | 59 | 134 | Chr.9 (14,116.9) |
|  | R-GCCGTGGCGGCATGTTCTCA |  |  |  |
| SICAAS9109 | F-AATGGGACAACAACATGCAG | 59 | 191 | Chr.9(14,461.1) |
|  | R-GTGAAAGAAGCCAAGCCATC |  |  |  |
| SICAAS9118 | F-ACCTGCGGTCAGGAATCA | 57 | 258 | Chr.9(14,963.3) |
|  | R-CTGATGGTGCTGCTTCTCG |  |  |  |
| SICAAS9110 | F-TTGACACGGTCCCTTCCTAC | 55 | 257 | Chr.9(15,489.8) |
|  | R-CTTGGAGGTGTCGCTTCTTG |  |  |  |
| SICAAS9119 | F-CCTGTCACGCCTCCTACAGT | 60 | 173 | Chr.9(15,562.4) |
|  | R-CCACGATTTAGGGTGAGATGAG |  |  |  |
| SICAAS9112 | F-CTAGCAACCCAAAACCTTAACCA | 58 | 117 | Chr.9(16,213.3) |
|  | R-GATGAGTAGTCGAGGACGAAGG |  |  |  |
| SICAAS9051 | F-GCCACTGTTTGGGTAGCTGGACA | 59 | 147 | Chr.9 (16,751.5) |
|  | R-TGAGACTACTTCTGGATCCACTGAGC |  |  |  |
| SICAAS9088 | F-GACTACAACTCCGACGACGAC | 61 | 170 | Chr.9(17,189.9) |
|  | R-GTGGAGACACCCTCTTCTTCCT |  |  |  |
| SICAAS9052 | F-CCTTCTTCCACGAGGACCTTACCA | 59 | 126 | Chr.9 (17,440.4) |
|  | R-TGTGCCGGCTGTCGTGTATGC |  |  |  |
| SICAAS9113 | F-AGCACTGTTTCTCCTTAAACGAAG | 58 | 146 | Chr.9(18,066.6) |
|  | R-AGCTGCGTATGTAGTCGTCGTC |  |  |  |
| SICAAS9080 | F-ACCATTTGGCAATCAAAGGTGTTGAAG | 55 | 181 | Chr.9 (20,103.5) |
|  | R-TGCAGGTGGCACCAACAAAAATCA |  |  |  |
| SICAAS9120 | F-ATCAAAATTACCCACTGCCACT | 56 | 215 | Chr.9(20,517.7) |
|  | R-AATCACCAGGCGGAGGTAG |  |  |  |
| SICAAS9081 | F-GCCAAAACTTTCGGGGCTTGTTG | 57 | 180 | Chr.9 (21,103.1) |
|  | R-TGTGTTCCTAACTTTGGTGCCCCT |  |  |  |
| SICAAS9121 | F-GAGTTGTTGAAGGAGGACCAGT | 58 | 115 | Chr.9(21,129.7) |
|  | R-GTATGTTGTCCATTGTGGCTGT |  |  |  |
| SICAAS9053 | F-GGGCACAAACACAAATGCCCCA | 57 | 270 | Chr.9 (22,181.6) |
|  | R-CACGCAGTTTGTGTCACAGGCA |  |  |  |
| SICAAS9054 | F-GGGATTTGGGTGGGGACGGC | 59 | 168 | Chr.9 (23,440.3) |
|  | R-CGCGCCACGCCTCTGTAACT |  |  |  |
| SICAAS9055 | F-GCCGTTGCATGCGAAGCAATCA | 57 | 268 | Chr.9 (25,084.4) |
|  | R-TCAGCTCGTATCACTTTCACAACCAC |  |  |  |
| SICAAS9056 | F-TGGACAGTGCTTGAGCAGTTGAG | 57 | 196 | Chr.9 (28,878.5) |
|  | R-ACAAGGAGTCCTGAATACAAGGGCA |  |  |  |
| SICAAS9057 | F-GCCCGAAGAAGCGGTGTCTGA | 59 | 197 | Chr.9 (33,425.2) |
|  | R-TGCTGGAGCTTGTGTCACTTCCA |  |  |  |
| SICAAS9114 | F-CTAGCAAAAGAAAACCGCATTG | 56 | 269 | Chr.9(34,973.8) |
|  | R-CTAGACACACCATCGACCAAGA |  |  |  |
| SICAAS9089 | F-CTCATCTCGCTCGTCTCGTCT | 61 | 154 | Chr.9(35,492.2) |
|  | R-GATTACACTGGTCAGAGGTCAGAG |  |  |  |
| SICAAS9101 | F-TTCAGAGACACCTGTGGGAGTA | 58 | 160 | Chr.9(36,106.5) |
|  | R-TGTTCTATTGGTGAACACACCTG |  |  |  |
| SICAAS9102 | F-TTCAGAGACACCTGTGGGAGTA | 58 | 160 | Chr.9(37,193.4) |
|  | R-TGTTCTATTGGTGAACACACCTG |  |  |  |
| SICAAS9082 | F-AGAGGAGCCGCCAAGAGAGAGC | 61 | 158 | Chr.9 (37,297.8) |
|  | R-CGCCGACGCCGACATCCTAC |  |  |  |
| SICAAS9111 | F-AGAAATCCATGCCAAAGAGC | 55 | 228 | Chr.9(39,088.5) |
|  | R-CAAGTGCAAGTGTGGGGAAT |  |  |  |
| SICAAS9122 | F-GTCAATCTAGGGAGTGGAGGTG | 58 | 227 | Chr.9(39,164.5) |
|  | R-AACAGTAAACGAGGGCAGAAAG |  |  |  |
| SICAAS9131 | F-TCATAGGATGTTGGCGTGTG | 57 | 221 | Chr.9(41,032.4) |
|  | R-GCCTGAATTACCAGGTGAACA |  |  |  |
| SICAAS9123 | F-AGGTGACTGTTGGAGTTGAAGA | 58 | 196 | Chr.9(42,456.3) |
|  | R-CACCAAATCTACCCTTTCCTCT |  |  |  |
| SICAAS9103 | F-CATGGCTTTTTGACCCAACTAC | 58 | 178 | Chr.9(43,770.7) |
|  | R-AGGAGGGAGAAAGAAGAAAGCA |  |  |  |
| SICAAS9058 | F-ACACGGTCAGTCACGCAGGTCT | 59 | 115 | Chr.9 (44,003.2) |
|  | R-CAGCTGCAGTGGTGGAGGAACG |  |  |  |
| SICAAS9059 | F-TTGTTCGTTCCTCCACCACTGC | 57 | 124 | Chr.9 (44,003.3) |
|  | R-GGCCCGAGCCTTTACCATCCAC |  |  |  |
| SICAAS9124 | F-CTTGTGTGAACTGCTGAATTAGG | 57 | 271 | Chr.9(45,500.0) |
|  | R-GCTTGTTGATCTCAGGTGGA |  |  |  |
| SICAAS9104 | F-GCCCTGATTGGTATTCATCTTTTG | 58 | 172 | Chr.9(45,879.8) |
|  | R-TGTGGCAGAATGGTTAGAGTTAGA |  |  |  |
| SICAAS9061 | F-TCGAAGGCAAGCCGCAACGA | 57 | 114 | Chr.9 (46,205.3) |
|  | R-CCCGCCGCCAACCTCAAGAC |  |  |  |
| SICAAS9125 | F-GGCTGGTATGTCCTCTCACC | 57 | 204 | Chr.9(48,032.4) |
|  | R-GCGGCTGAAGATGGATAAGA |  |  |  |
| SICAAS9126 | F-CTGAAGTTCGTACGTAGTCTGTTAGT | 58 | 263 | Chr.9(48,504.7) |
|  | R-GCATCGCCACTACATTCATAGA |  |  |  |
| SICAAS9105 | F-TGCCGTCTCCTCTCTAGCAC | 59 | 136 | Chr.9(48,541.6) |
|  | R-CCGAAAGCCACCTACTACCA |  |  |  |
| SICAAS9127 | F-GAAGGCGACGAAGAGGAA | 57 | 251 | Chr.9(49,535.2) |
|  | R-TTCGAGTACAAGAGGTGAAGCA |  |  |  |
| SICAAS9090 | F-GTTCCTTCGTGGCTTTCTTG | 57 | 194 | Chr.9(51,618.8) |
|  | R-GGTGATGTGCACCCTCTTG |  |  |  |
| SICAAS9096 | F-GGCGCTCGTATATATGGGTCT | 60 | 179 | Chr.9(51,725.3) |
|  | R-GTACATGCGACCCTCTTATGGT |  |  |  |
| SICAAS9091 | F-CTCCAGGTTTTGGTTTCAGAGAT | 58 | 247 | Chr.9(52,011.1) |
|  | R-ACGATAAGAAACAACTGCACTGTC |  |  |  |
| SICAAS9062 | F-GGTTGGCATTGAACTGGGAGCA | 57 | 123 | Chr.9 (52,248.5) |
|  | R-CGGACACCCACACACTGCAACA |  |  |  |
| SICAAS9092 | F-CTCCTAGCCCTAAACCCTTTTC | 58 | 149 | Chr.9(52,878.7) |
|  | R-GAGATGGGGAACATTATTGACATAC |  |  |  |
| SICAAS9093 | F-CAACAACTTCATCGGAACTGTAAG | 58 | 138 | Chr.9(53,464.4) |
|  | R-CACTGGTGAAGGGAAACACTTTA |  |  |  |
| SICAAS9106 | F-CCACAAAAACAAGTTCGCAAG | 56 | 169 | Chr.9(54,936.6) |
|  | R-CTGTTCTGATGCAGTGACGAC |  |  |  |
| SICAAS9128 | F-CCCGTGCAATCAGGATACAT | 55 | 146 | Chr.9(54,994.7) |
|  | R-AAAGCAGGGGAAAGGGATT |  |  |  |
| SICAAS9063 | F-AGGACGCAAGGACGCAAGAAC | 57 | 111 | Chr.9 (55,288.1) |
|  | R-TGCCCGCGGCATGGTTTGTA |  |  |  |
| SICAAS9084 | F-TCGTTCATTGGGCTGGCTCTCTC | 59 | 138 | Chr.9 (55,370.6) |
|  | R-ACCCGGCATGTTCTTCGCCAAC |  |  |  |
| SICAAS9064 | F-TGGTCCCAATCCAGACTTACCCTGT | 57 | 191 | Chr.9 (55,450.7) |
|  | R-AACTTGACGGCTCTGTGGAGTTC |  |  |  |
| SICAAS9066 | F-GCGAGAAAGATCGGCGAGAGCA | 57 | 181 | Chr.9 (55,493.1) |
|  | R-GACCTTGCCCGGGCGATAACA |  |  |  |
| SICAAS9129 | F-CCAATTTTCCGTCTCACTGC | 55 | 270 | Chr.9(56,447.8) |
|  | R-AAACGGTGGTTTGAGTTTCG |  |  |  |
| SICAAS9094 | F-ATGCTAGGTGGGATGTTTGTTC | 58 | 212 | Chr.9(57,015.3) |
|  | R-CACGACACTGGTTATTTCTCCA |  |  |  |
| SICAAS9085 | F-TGCACGGGATTATTCGGTACCACG | 59 | 169 | Chr.9 (57,721.4) |
|  | R-GGGGGCTCGGGTATATTCAGGT |  |  |  |
| SICAAS9067 | F-GCTAGGCGGCTTCCCAAGGC | 61 | 164 | Chr.9 (58,046.7) |
|  | R-GCCGCCATCTCCTCCCCGTA |  |  |  |
